# Contrasting plant trait strategies underpin ecosystem stability in temperate and alpine grasslands

**DOI:** 10.1038/s41467-026-78537-2

**Published:** 2026-10-08

**Authors:** Pu Yan, Nianpeng He, Changjin Cheng, Yonghui Wang, Enrique Valencia, Congcong Liu, Lawren Sack, Lin Jiang

**Affiliations:** 1 School of Biological Sciences, Georgia Institute of Technology, Atlanta, GA USA; 2 Key Laboratory of Boreal Forest Ecosystem Conservation and Restoration, National Forestry and Grassland Administration, Northeast Forestry University, Harbin, China; 3 South China National Botanical Garden, Guangzhou, China; 4 Ministry of Education Key Laboratory of Ecology and Resource Use of the Mongolian Plateau and Inner Mongolia Key Laboratory of Grassland Ecology, School of Ecology and Environment, Inner Mongolia University, Hohhot, China; 5 Department of Biodiversity, Ecology and Evolution, Faculty of Biological Science, Complutense University of Madrid, Madrid, Spain; 6 College of Life and Environmental Sciences, Minzu University of China, Beijing, China; 7 Department of Ecology and Evolutionary Biology, University of California, Los Angeles, CA USA

**Keywords:** Community ecology, Ecosystem ecology, Grassland ecology

## Abstract

Plant functional traits are key drivers of ecosystem stability, yet the extent to which trait-stability relationships vary among different ecosystems remains poorly understood. Here, we provide a comparative analysis of trait-stability relationships across temperate and alpine grasslands. By integrating in situ measurements of plant functional traits to satellite-derived indices of ecosystem temporal stability, resistance, and resilience for the two types of grasslands in China, we find strikingly biome-dependent patterns. In temperate grasslands, where fast-growing, resource-acquisitive annuals are more prevalent, acquisitive strategies enhance ecosystem temporal stability and resistance. In contrast, in alpine grasslands where annuals are less common, these same strategies reduce ecosystem temporal stability and resistance, and perennial species with slow-growing, conservative traits play the stabilizing role. Ecosystem resilience, however, shows consistent positive responses to acquisitive traits in both grassland types. These patterns persist after accounting for the effects of plant diversity, climate and soil properties. Our findings challenge the conventional view that only conservative plant growth strategies promote ecosystem resistance and temporal stability, and instead highlight distinct pathways to stability across ecosystems—much like the many roads that once led to Rome.

## Abstract (web summary)

Stable grasslands are vital for sustaining ecosystem functions under environmental change. The study shows that plant functional traits support stability through contrasting pathways in temperate and alpine grasslands.

## Introduction

Understanding the determinants of ecosystem stability, which encompasses multiple dimensions, such as temporal stability (the extent to which a system remains invariant over time), resistance (the capacity of a system to withstand perturbations), and resilience (the ability of a system to return to its original state following perturbations), has long intrigued ecologists^1–4^. These stability properties characterize the ability of ecosystems to maintain their functions and services in the face of environmental change and variability, are thus of fundamental importance to humanity. This inquiry has gained urgency in the face of escalating global environmental changes, which are increasingly influencing natural and managed ecosystems^5^. Substantial research has shown that ecosystem stability is affected by both abiotic (e.g., climate) and biotic (e.g., species diversity) factors^6–12^. Variation in abiotic conditions may alter plant physiology, phenology, and growth, thereby driving temporal changes in ecosystem functioning (e.g., productivity)^7,13^. Biotic factors may further influence stability by modifying how species respond to environmental change^14^. However, it is increasingly evident that the effects of these factors are expressed through the functional traits of the co-occurring species^4,6^. Indeed, functional traits define species niches^15^ by regulating how plants perform under various abiotic and biotic conditions, thereby determining where they can persist and how they interact with co-occurring species. Functional traits also regulate species’ sensitivities to environmental change and subsequent recovery^14,16^, which, in turn, determine resistance, resilience and temporal stability of ecosystems. Consequently, trait-based approaches have emerged as a promising framework for gaining further insight into the mechanisms underpinning ecosystem stability^4^. However, despite much progress^6,10,17–21^, our understanding of trait-stability relationships remains fragmented and incomplete, as studies often examine isolated traits or single dimensions of stability within limited ecological contexts, limiting synthesis across systems.

The prevailing hypothesis linking plant functional traits to ecosystem stability posits that ecosystems dominated by slow-growing, resource-conservative species—characterized by traits such as high leaf dry matter content (LDMC) and low specific leaf area (SLA)—tend to be more resistant to disturbance and exhibit greater temporal stability^4,6,22^. This hypothesis is grounded in the stress tolerance of resource-conservative (slow) species, which, due to their low metabolic rate and high tissue density, can better endure physiological stress and survive adverse environmental conditions. Consistent with this hypothesis, several studies have reported positive relationships between conservative plant strategies and ecosystem temporal stability^6,7,23,24^. However, emerging evidence suggests that plants with resource-acquisitive (fast) strategies, known for rapid growth and fast tissue turnover^25^, may also provide advantages under adverse environmental conditions. For example, extreme droughts in North American grasslands have favored acquisitive plants, driving community functional shifts toward acquisitive strategies^26^. A recent global study on plant traits in drylands found that high-aridity environments support a mix of contrasting functional strategies, with co-occurring conservative and acquisitive species^27^. These findings align with the fact that species with acquisitive strategies can rapidly use available resources to complete their life histories before the onset of stressful conditions (i.e., stress escape), and minimize the loss of limiting resources (e.g., water conservation via reduced transpiration) in stressful environments (i.e., stress avoidance)^28,29^. Consequently, acquisitive strategies may also have the potential to contribute to ecosystem stability^30^. The stabilizing effects of traits likely depend on ecological context—for example, the community’s trait composition, resource availability, and disturbance regime—as these factors shape how individual-level responses scale up to ecosystem-level dynamics^31,32^. Thus, the relative importance of conservative versus acquisitive strategies in promoting ecosystem stability could vary across ecosystems. However, little is known about the circumstances under which conservative versus acquisitive strategies play the greater role in promoting ecosystem stability, which represents an important knowledge gap in trait-stability relationships.

To address this gap, we examined how conservative and acquisitive plant strategies relate to multiple dimensions of ecosystem stability (temporal stability, resistance, and resilience) under contrasting environmental conditions represented by China’s temperate and alpine grasslands (Fig. S1). Temperate and alpine grasslands account for approximately 40% and 10% of the global grassland area, respectively^33,34^, yet they differ markedly in both abiotic and biotic conditions. Alpine grasslands are characterized by cold, short growing seasons and strong thermal stress, whereas temperate grasslands experience comparatively warmer and longer growing seasons. In our study regions, these climate contrasts are mirrored by differences in species compositions: temperate grasslands contain a higher proportion of fast-growing annual plants, while alpine grasslands are dominated by slow-growing perennial species adapted to harsh environments^35^. Together, these consistent climate and biotic contrasts between the two grasslands types provide a valuable opportunity for testing whether trait-stability relationships depend on ecosystem context.

In this work, we compile standardized survey data collected from 160 plots across 20 grassland sites (10 sites per grassland type; Fig. S1), including plant community structure and 15 leaf, stem, and root traits (Table S1) measured in situ for 343 species. We further use 23 years (2000–2022) of Standardized Precipitation–Evapotranspiration Index (SPEI) and Enhanced Vegetation Index (EVI) data to quantify multiple ecosystem stability dimensions, including temporal stability, resistance, and resilience. We test whether the relative importance of conservative versus acquisitive plant strategies for ecosystem stability differs between temperate and alpine grasslands. We show that acquisitive strategies are associated with greater temporal stability and resistance in temperate grasslands but lower temporal stability and resistance in alpine grasslands, where conservative strategies instead play the stabilizing role. In contrast, acquisitive strategies are associated with greater resilience in both grassland types. Together, our results reveal biome-dependent pathways to ecosystem stability and demonstrate that the stabilizing role of plant functional strategies depends on both environmental context and the dimension of stability considered.

## Results

### Plant functional composition differs between temperate and alpine grasslands

**Fig. 1:**
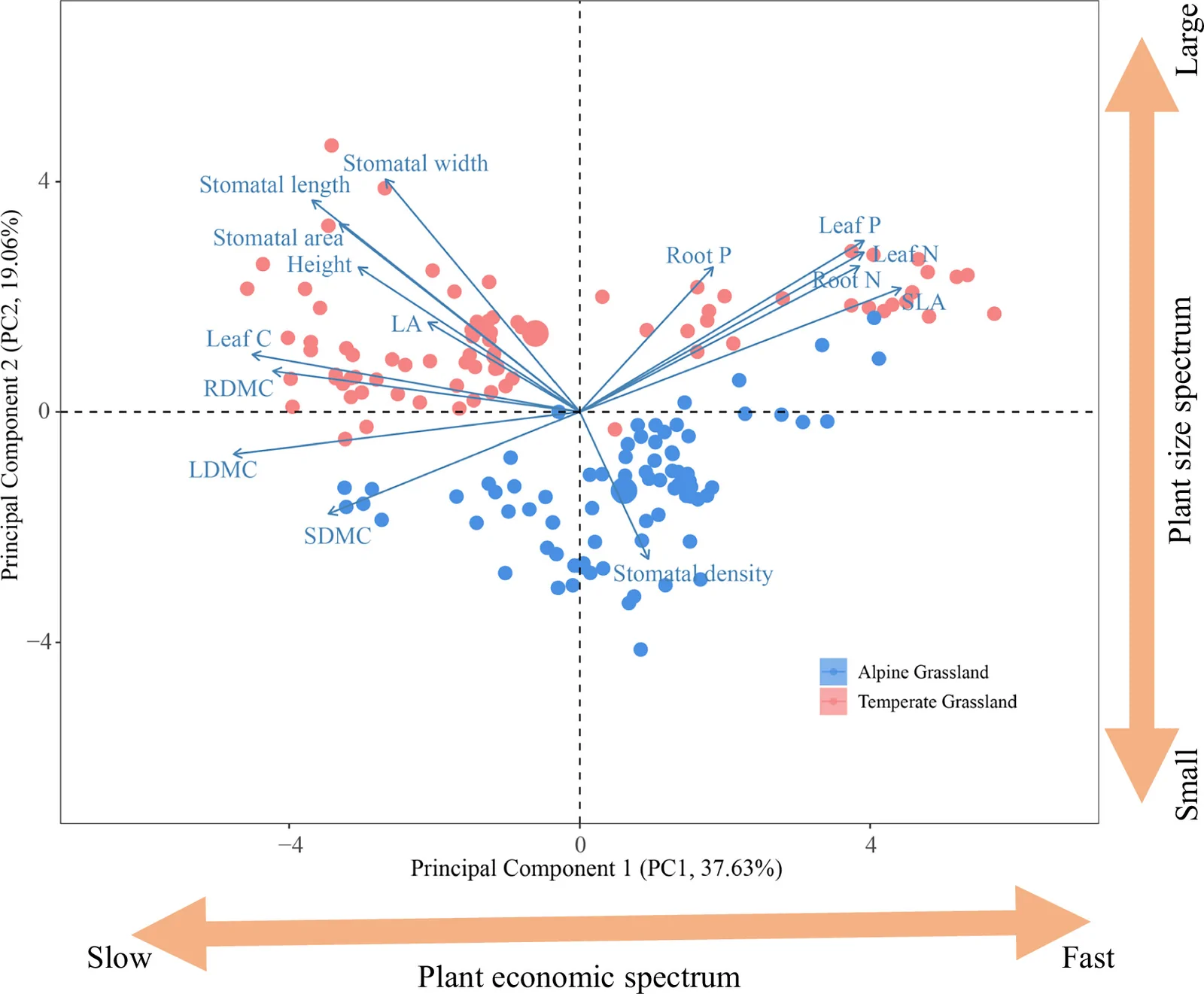
The biplot illustrating the key dimensions of multivariate trait space. Principal component analysis (PCA) was conducted on 16 plant traits across 160 plots to summarize the key dimensions of trait variation. These traits included leaf carbon (Leaf C, %) and nitrogen (Leaf N, %) concentrations, leaf phosphorus concentration (Leaf P, mg g^−1^), leaf area (LA, cm²), leaf dry mass content (LDMC, %), plant height (Height, cm), specific leaf area (SLA, cm² g^−1^), stem dry mass content (SDMC, %), root nitrogen concentration (Root N, %), root phosphorus concentration (Root P, mg g^−1^), and root dry mass content (RDMC, %). All traits were standardized prior to analysis. The first principal component axis represents the economic spectrum of plants, while the second principal component axis reflects the size spectrum. Arrow lengths indicate the relative loading of each trait onto the respective axes. Source data are provided as a Source Data file.

As expected, growing season temperature was significantly higher in temperate than alpine grasslands (Fig. S2 and Table S2). However, the two grassland types did not differ in precipitation or soil properties (Fig. S2 and Table S2). Temperate and alpine grasslands also did not differ in plant species richness (Table S2). On the other hand, plant functional composition differed significantly between temperate and alpine grasslands at both plot and site levels (Figs. 1 and S3, Table S3), with plants in temperate grasslands being significantly taller and possessing greater stomatal areas compared to those in alpine grasslands (Fig. S4 and Table S2). In addition, the proportion of annual plants was significantly greater in temperate grasslands than in alpine grasslands, accounting for 27% vs. 9% of species richness, and 32% vs. 7% of aboveground biomass, respectively (Fig. S4, Tables S2 and S4). Temperate grasslands also tended to comprise significantly greater proportions of grasses than forbs compared to alpine grasslands (Fig. S5). Importantly, principal component analysis (PCA) revealed that plant communities in both grasslands were distinctly distributed along the economic spectrum, with the first principal component (PC1) separating resource-conservative strategies (characterized by high LDMC, leaf carbon concentration [Leaf C], stem dry mass content [SDMC], and root dry mass content [RDMC]) from resource-acquisitive strategies (characterized by high SLA, leaf nitrogen concentration [Leaf N], leaf phosphorus concentration [Leaf P], root nitrogen concentration [Root N], and root phosphorus concentration [Root P]), with consistent patterns observed at both plot and site levels (Figs. 1 and S3).

### Trait-stability relationships differ between temperate and alpine grasslands

**Fig. 2:**
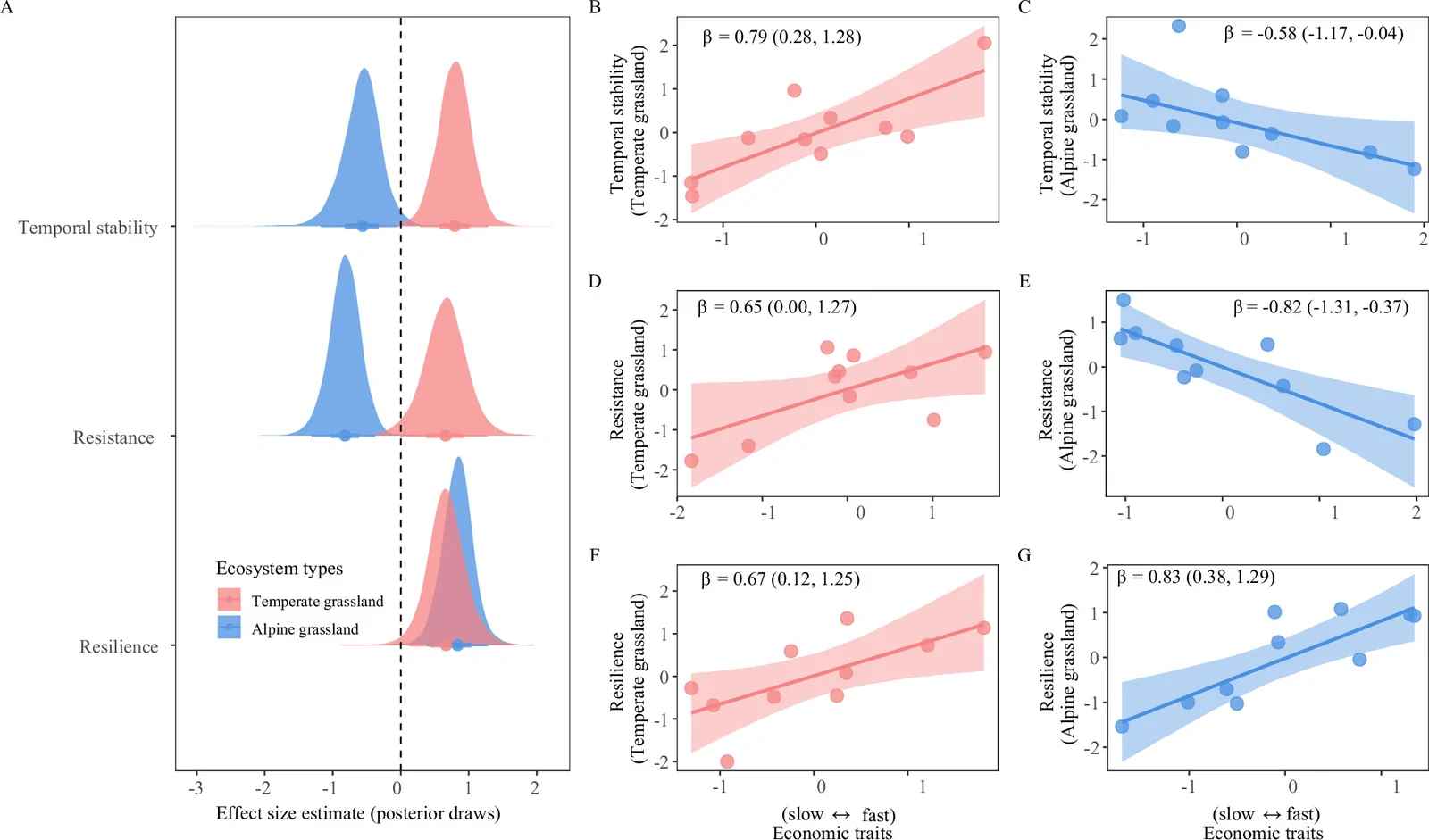
Effects of economic traits on ecosystem temporal stability, resistance, and resilience at the site level. **A** Shows posterior distributions of standardized effect sizes; circles indicate posterior means, with 80% (thick lines) and 95% (thin lines) credible intervals from multivariate Bayesian models. **B**–**G** Show relationships between economic traits and temporal stability, resistance, and resilience. Each point represents a site, and each panel reports the standardized slope (*β*) with its 95% credible interval from the corresponding Bayesian model. Solid lines and shaded bands indicate the posterior mean prediction and its 95% credible interval, respectively. Economic traits are summarized as PC1 from a principal component analysis, where higher values indicate more acquisitive strategies and lower values indicate more conservative strategies. Source data are provided as a Source Data file.

**Fig. 3:**
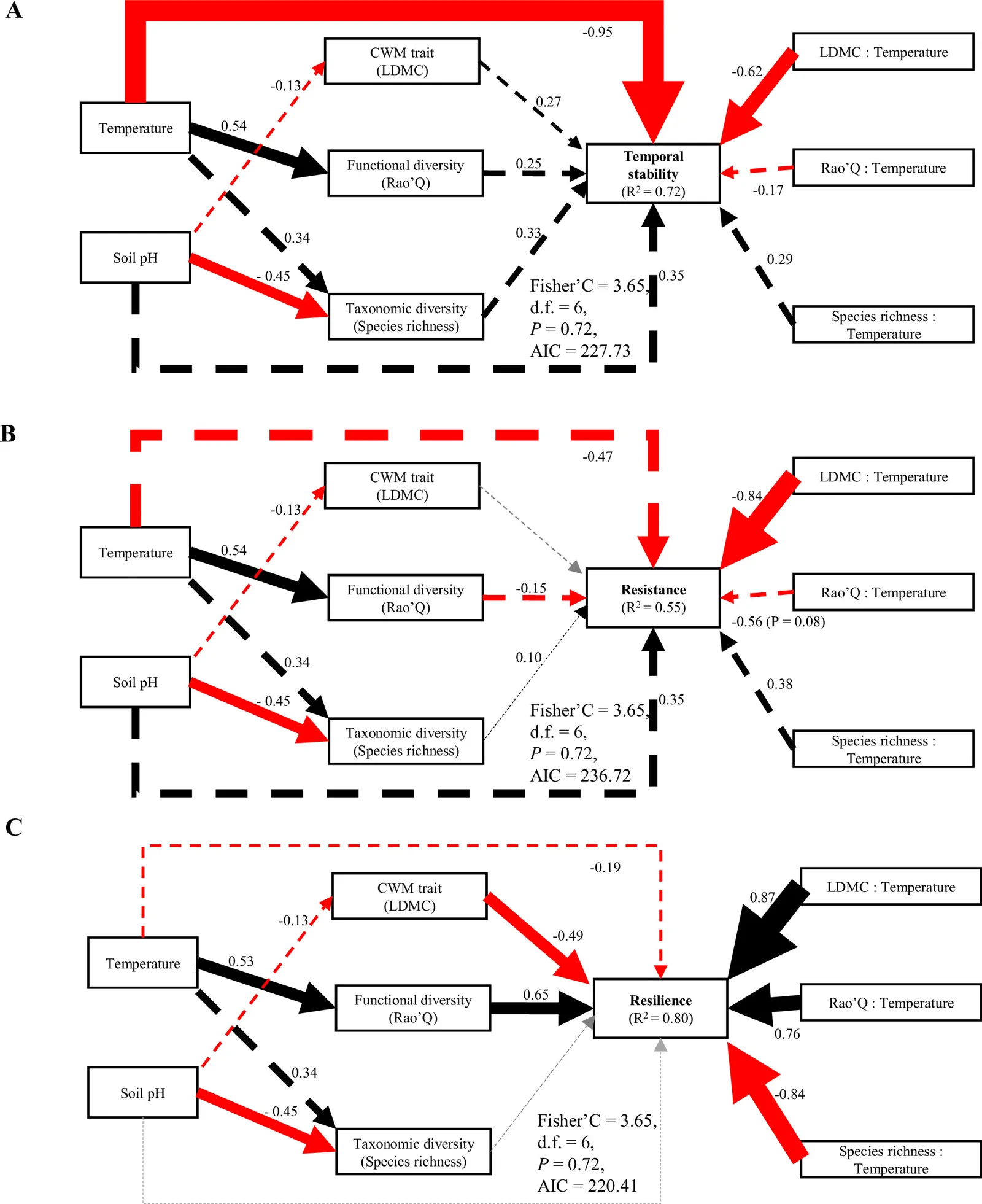
Direct and indirect effects of economic traits, plant diversity, and environmental conditions on grassland stability. Results of Bayesian piecewise structural equation models fitted at the site level using pooled data from temperate and alpine grasslands, showing the direct and indirect effects of mean annual temperature, soil pH, taxonomic diversity (species richness), functional diversity (Rao’s Q), and leaf dry matter content (LDMC), including the LDMC × temperature interaction, on temporal stability (**A**), resistance (**B**), and resilience (**C**). LDMC was used as a parsimonious proxy for conservative–acquisitive plant strategies in these site-level models, and temperature was included as the focal climatic driver because it was the measured environmental variable that most clearly differentiated the two grassland types. Arrow widths correspond to the magnitude of standardized path coefficients. Black and red arrows indicate positive and negative relationships, respectively; solid lines denote effects whose two-sided 95% Bayesian credible intervals excluded zero, whereas dotted lines denote effects whose credible intervals included zero. Fisher’s C and d-separation statistics are shown as complementary diagnostics of model fit. Source data are provided as a Source Data file.

At the site level, community-level economic traits were strongly associated with ecosystem temporal stability, resistance, and resilience, but the direction of these relationships differed between temperate and alpine grasslands. In temperate grasslands, a shift toward more acquisitive strategies was associated with greater temporal stability and resistance, whereas in alpine grasslands the same trait shift was associated with reduced temporal stability and resistance (Figs. 2, S6, Tables S5 and S6). By contrast, resilience showed consistently positive relationships with economic traits in both grassland types (Figs. 2, S6, Tables S5 and S6). Across the three stability components, community-weighted economic traits, including LDMC, leaf P, and SLA, had effects comparable to or exceeding those of abiotic factors, and substantially stronger than those of taxonomic and functional diversity (Figs. S7 and S8). Bayesian structural equation models, which accounted for direct and indirect effects of climate and soil properties, further supported these site-level patterns (Fig. 3), with model-predicted LDMC × temperature relationships shown in Fig. S9. Supplementary plot-level analyses yielded qualitatively similar results (Figs. S10 and S11, Table S7).

### Independent datasets support the biome-dependent patterns

To further assess the robustness of our findings, we analyzed two additional, independent transect datasets consisting of a larger number of study sites (temperate, *n* = 35; alpine, *n* = 33) spanning the same regions and vegetation types (Fig. S12). Species composition from published sources was matched to our standardized, field-measured trait database, and community-weighted traits and stability metrics were derived using the identical workflow as in the main survey. These additional analyses confirmed biome-dependent relationships between community economic traits and both temporal stability and resistance (Text S1, Figs. S13 and S14).

## Discussion

Our study provides novel insights into how plant traits influence ecosystem stability—particularly temporal stability and resistance—in a biome-dependent manner across temperate and alpine grasslands. While alpine grasslands with increasingly conservative strategies exhibited higher temporal stability and resistance, temperate grasslands with increasingly acquisitive strategies demonstrated greater ecosystem temporal stability and resistance. The observed trait effects were comparable to those of abiotic variables and consistently exceeded the influence of plant diversity, which has often been emphasized as a key driver of ecosystem stability^36,37^. While previous studies have reported that diversity–stability relationships can be modulated by abiotic conditions, such as aridity^12,38^, our study provided empirical evidence of opposing trait-stability relationships across ecosystems. Unlike previous large-scale studies that typically rely on trait data from online databases, our analyses were grounded in trait measurements collected in situ, allowing for a more precise representation of plant functional strategies within their native environments^39^. Most importantly, our findings challenge the conventional paradigm that conservative, but not acquisitive, strategies foster ecosystem stability^4,6,22,40^ and underscore the need for future studies to examine trait-stability relationships across various ecosystems.

The opposing trait-stability relationships observed in our study highlight shifts in the dominant strategies conferring ecosystem stability in different environments: conservative strategies in alpine grasslands and acquisitive strategies in temperate grasslands. Resource-conservative, stress-tolerant species are traditionally considered less vulnerable to climate extremes and disturbances, such that communities dominated by these species are expected to exhibit greater temporal stability and resistance^4,6^. Our findings from alpine grasslands support this idea, adding to the growing number of studies reporting positive relationships between conservative strategies and ecosystem temporal stability^6,7,23,24^. However, our results from temperate grasslands suggest that resource-acquisitive strategies may offer an alternative pathway to higher ecosystem temporal stability and resistance. While the shorter growing season and lower temperatures in alpine grasslands favor stress-tolerant, resource-conservative perennial species^41^, temperate grasslands, with longer growing season and milder climate, support a higher proportion of annual plants. Our data indicate that annuals tend to exhibit faster trait syndromes (e.g., large SLA; see Fig. S15)^42,43^, consistent with rapid growth and turnover as part of a fast life-history strategy, which is classified as acquisitive in the context of the plant economics spectrum. Such fast strategies may also reflect phenological “escape” from peak competition, whereas investments linked to resource capture under crowding can be more pronounced in perennials. Annuals therefore contributed significantly to increased community-level acquisitive issue economics in our study, particularly in temperate grasslands (see Figs. S16 and S17). These traits enable annuals to rapidly complete their life cycles and avoid prolonged stress (i.e., stress escape). In addition, we found that annual plants in temperate grasslands tended to have smaller stomata (see Fig. S15), a trait associated with more rapid stomatal responses in the face of environmental stress (i.e., stress avoidance)^44^, which could also contribute to greater ecosystem temporal stability. By contrast, stomatal traits did not significantly influence ecosystem stability in alpine grasslands (see Fig. S7). Note unlike the observed positive relationships between the abundance of annual species and ecosystem stability in temperate grasslands, we did not detect any significant relationship between the grass/forb ratio and ecosystem stability in either grassland type (Fig. S17). Overall, our findings suggest that conservative and acquisitive strategies, each reflecting a different approach to coping with environmental stress, represent distinct pathways to stability across ecosystems. More broadly, our analyses suggest that the strategy most likely to promote stability depends on the dominant environmental constraint. Where stress is persistent, and resource supply is chronically constrained, conservative traits may enhance resistance and temporal stability by maximizing stress tolerance. Where resources are less chronically limiting but fluctuate through time, acquisitive traits may enhance resistance and temporal stability by enabling strategies for stress escape and avoidance. The generality of these findings, however, remains to be tested across a broader range of ecological systems and environmental contexts.

In contrast to the opposing trait-stability relationships observed for temporal stability and resistance, grasslands dominated by more acquisitive plants exhibited greater resilience in both temperate and alpine systems. Resource-acquisitive plants are known for their fast resource use, rapid growth, and short reproduction cycles^25^. These traits allow them to effectively exploit temporarily increased resources that often follow climatic perturbations (e.g., precipitation anomalies)^45^, enabling ecosystems dominated by these plants to restore functions more quickly after climate extremes^46^. This positive link between resource-acquisitive traits and ecosystem resilience, observed in our study and several other studies of grasslands^17,47–49^, may represent a broader pattern that should be tested across a wider range of ecosystems. Note that although resistance and resilience reflect different components of stability, our supplementary site-level analysis suggests that resistance was more closely associated with temporal stability than resilience (Figs. S18 and S19). Thus, the opposing trait associations with resistance and resilience in alpine grasslands likely translate into overall stability mainly through the resistance pathway. This pattern is consistent with emerging theoretical and empirical evidence that temporal stability can often be predicted more strongly from resistance than from post-disturbance resilience^37,50^.

Besides plant traits, climatic variables—most notably temperature—imposed strong yet opposing effects on ecosystem temporal stability in the two grassland types we studied. In alpine grasslands, warmer growing seasons were associated with lower temporal stability, echoing alpine warming experiments showing that elevated temperatures tended to destabilize grassland biomass production^8,10^. In temperate grasslands, however, higher temperatures were associated with greater temporal stability. This likely reflects regional climate dynamics, where higher temperature often coincides with reduced annual precipitation, intensifying aridity and favoring the proliferation of fast-growing annuals^51^. These acquisitive species can then buffer inter-annual fluctuations in productivity through stress escape and avoidance strategies^30^, thereby bolstering ecosystem stability. Our results also showed that soil properties influenced temporal stability in an ecosystem-dependent manner, with higher soil pH associated with lower stability in temperate grasslands but greater stability in alpine grasslands (Figs. 3 and S11). As soil pH governs nutrient availability—acidic soils reduce the availability of phosphorus, calcium, and potassium, whereas alkaline soils reduce the availability of iron and manganese^52^—these contrasts suggest that different nutrient limitations may underlie stability in each ecosystem^53,54^. In temperate grasslands, elevated pH may alleviate phosphorus limitation, enabling plant communities to respond more dynamically to fluctuations in other limiting resources (e.g., water), thereby reducing ecosystem temporal stability^55^. In contrast, in alpine grasslands—where micronutrient availability is already low—higher pH may further restrict micronutrient access, potentially constraining plant responsiveness to other environmental variability and thus enhancing stability^54,56,57^. Long-term pH manipulation or nutrient addition experiments, coupled with repeated monitoring of plant traits and community composition, will be essential to clarify the causal links between soil chemistry and grassland stability^53^.

In this study, we compared temperate and alpine grasslands to represent two broadly contrasting ecosystem types with distinct climatic and biological characteristics. We acknowledge that differences in species composition are not unique to this contrast, as grasslands within the same biome but in different regions can also differ substantially in floristic composition; thus, our comparison should be interpreted as capturing broad environmental and life-history differences rather than isolating biome identity per se. Furthermore, because our temperate and alpine grasslands are located in different geographic regions, the grassland-type contrast may also reflect spatially structured context that covaries with location (e.g., latitude gradients and regional productivity regimes). However, models controlling for latitude and aboveground biomass (as a proxy of productivity) yielded qualitatively similar trait-stability relationships (Fig. S20), suggesting that our conclusions are not driven solely by broad geographic gradients. More broadly, we recognize that the same functional traits may reflect different ecological strategies across ecosystem types, such that traits associated with stress tolerance in grasslands may arise for different reasons in other systems. For example, conservative traits, such as low SLA, may also occur in relatively low-stress environments, including rainforests, where long tissue lifespan and slow turnover are favored. We therefore interpret conservative and acquisitive trait syndromes here within the more restricted context of grasslands along environmental gradients, where ecosystems are broadly comparable in growth form and where trait differences are more likely to reflect contrasts in abiotic stress, resource limitation, and life-history composition. We further acknowledge that biotic interactions (e.g., herbivory, pathogen pressure, and competitive intensity) may also vary among sites and have the potential to influence trait-stability relationships. Here, we treat grassland type as an integrated ecosystem context and encourage future studies to more explicitly consider the roles of biotic interactions in shaping trait-stability relationships.

Another caveat, shared with many large‑scale observational studies^6,12,21^, is that our plant trait data were collected in a single year, whereas the stability metrics reflect ecosystem dynamics spanning multiple decades. This mismatch precludes assessment of the roles of temporal shifts in trait expression for ecosystem stability. Future research should incorporate repeated trait measurements to partition the contributions of temporal and intraspecific trait variation to ecosystem stability. Moreover, while our study focused on examining how plant traits influence ecosystem stability, ecosystem stability may also play a role in determining which traits persist and dominate, potentially creating a feedback loop between traits and stability. However, the observational nature of our study limits our ability to assess the relative importance of this bi-directional relationship. Experimental approaches will therefore be essential to disentangle these feedbacks. Finally, although our study sites were selected to minimize human disturbance, historical or low-level grazing may have occurred and could contribute to unexplained variation in vegetation dynamics. Because spatially explicit, time-resolved grazing data were not available for all sites, we could not directly account for grazing effects. Future studies combining long-term vegetation monitoring with direct measures of grazing intensity (e.g., stocking rates, dung surveys, exclosure experiments, or land-use datasets) will be essential to disentangle grazing effects from climatic drivers and plant functional strategies.

By linking plant economic traits to multiple dimensions of ecosystem stability across temperate and alpine grasslands, our study provides a more comprehensive and mechanistic understanding of trait-stability relationships. We show that resource-conservative and resource-acquisitive strategies offer distinct pathways to temporal stability and resistance in the two grassland types—much like the many roads that once led to Rome, each shaped by different starting points and conditions. These biome-dependent relationships emphasize the need to study trait-stability relationships across diverse ecosystems, and to develop management strategies tailored to ecosystems with distinct trait-stability patterns.

## Methods

### Study area and field survey

Our study focused on temperate grasslands of the Mongolian Plateau and alpine grasslands of the Tibetan Plateau, both representative of their distributions in China (Fig. S1). The Mongolian Plateau (40°N–50°N, 106°E–121°E) has elevations of 1000–1200 m, and experiences a temperate continental monsoon climate. We selected ten study sites evenly distributed across the Plateau, with an average growing season temperature of 14 °C and precipitation of 293 mm (Table S8). The Tibetan Plateau (25°N–40°N, 74°E–104°E) has an average elevation exceeding 4000 m, and is characterized by low temperatures and widespread permafrost. We also established ten study sites evenly across the Tibetan Plateau, with an average growing season temperature of 5 °C and precipitation of 296 mm (Fig. S1 and Table S8). Sites were selected to minimize recent human disturbance based on prior reconnaissance and local knowledge (including consultation with local herders and regional experts). Nevertheless, we cannot rule out historical grazing impacts at our study sites.

Field surveys were conducted in July and August 2018, coinciding with the peak growing season when most species are fully grown and functionally active. We surveyed a total of 160 plots across the 20 sites, located at least 80 km apart (Fig. S1). At each site, we established eight plots measuring 1 m × 1 m within a relatively uniform area of 1000 m × 1000 m. For each plot, we recorded plant species presence, species richness, plant height, and coverage, and harvested above-ground biomass for each species. Field sampling was authorized by the Institute of Geographic Sciences and Natural Resources Research, Chinese Academy of Sciences (CAS), and conformed to local governmental regulations.

### Plant traits

We measured plant functional traits in situ following standardized protocols (see Supplementary Text S2 for detailed sampling and measurement procedures)^58^. The resulting trait dataset includes measurements of 15 morphological and chemical traits that influence the economics of resource acquisition and retention for a total of 343 plant species. These traits include plant height (cm), leaf area (LA, cm²), which primarily reflect plant size and competitive ability for light; leaf carbon concentration (Leaf C, %); leaf nitrogen concentration (Leaf N, %); leaf phosphorus concentration (Leaf P, mg g⁻¹); root nitrogen concentration (Root N, %); root phosphorus concentration (Root P, mg g⁻¹), together with specific leaf area (SLA, cm² g⁻¹), which are commonly associated with resource-acquisition potential and fast tissue turnover; leaf, stem and root dry matter content (LDMC, SDMC, RDMC, %), which reflect tissue investment and are often linked to conservative strategies and stress tolerance; and stomatal length, stomatal width, stomatal area, and stomatal density, which indicate leaf gas-exchange capacity and potential sensitivity to water and temperature conditions (Table S2). All trait data were collected during our 2018 field survey, with replicate measurements per species within each site that were integrated to site-specific species-level trait values for community-level calculations, thereby retaining intraspecific variation across sites. While the broad spatial extent of our survey captures substantial regional variation in plant traits, we acknowledge that using data from a single year does not account for potential temporal variability in trait expression driven by interannual climatic fluctuations.

### Climatic factors and soil properties

We obtained precipitation and temperature data for the growing seasons (2000–2022) for each site from the CRU TS v4.09 gridded climate product (0.5° × 0.5°), extracting site-specific values at each site’s coordinates and aggregating monthly series to annual growing-season metrics. Because mean annual temperature and annual precipitation reflect long-term climatic baselines, their site-level estimates are expected to vary little with reasonable changes in the averaging window. We used 2000–2022 climate data to match the resistance/resilience calculations. Extending the time window to 2000–2022 increases the number of qualifying extreme climate events per site and therefore provides more robust and comparable site-level estimates—especially for resistance and resilience. We confirmed that climate estimates based on 2000–2018 were highly consistent with those based on 2000–2022 (Fig. S21). The length of the growing season at each site was determined based on accumulated site-specific temperature and precipitation (Text S3). We also quantified plot-level soil properties, including soil pH, bulk density, organic carbon, available nitrogen, and available phosphorus. Further details are provided in the Supplementary Materials (Text S4).

### Identifying climate extremes

We identified hydroclimatic extremes using the SPEI, which quantifies anomalies in growing-season water balance by jointly considering precipitation and atmospheric evaporative demand (i.e., potential evapotranspiration)^59^. Monthly SPEI data for each study site (2000–2022), based on geographical coordinates, were obtained from https://spei.csic.es/. Dry, normal, and wet conditions were defined as follows: normal (−0.67 < SPEI < 0.67), wet (SPEI > 0.67), and dry (SPEI < − 0.67)^37^. We used the SPEI to classify growing-season hydroclimatic extremes because it integrates precipitation anomalies with temperature-driven evaporative demand, thereby capturing water-balance stress that is mechanistically linked to grassland productivity. We adopted ±0.67 because it represents moderate moisture anomalies (approximately the lower/upper quartiles under a normal approximation), a threshold commonly used in grassland and dryland studies to balance ecological relevance with sufficient sample size of extreme events for robust estimation^9,37,60^. This choice is particularly appropriate for grasslands, where growing-season productivity and greenness are strongly constrained by water availability and drought–wetness conditions; SPEI captures this constraint while also incorporating temperature-driven evaporative demand, thereby providing a mechanistically relevant indicator of moisture stress.

To calculate resistance and resilience to extreme climate events, we first identified the climate status of each site for every year (e.g., dry, wet, or normal) as shown in Fig. S22. Based on these classifications, we selected extreme years using two criteria: (1) the year preceding an extreme event must be either of the same type or normal (i.e., no alternating extremes); and (2) the extreme year used for resilience calculations must be followed by a normal year. This approach, derived from Chen et al.^9^, aimed to minimize the confounding effects of consecutive or alternating wet and dry years on resistance and resilience calculations. Further details are provided in the Supplementary Materials (Text S5).

### Quantifying stability dimensions

We assessed three dimensions of ecosystem stability: temporal stability, resistance, and resilience. Temporal stability was calculated as the inverse of the coefficient of variation of EVI, a widely used metric of ecosystem productivity^61^. EVI data, obtained from the MOD13Q1 product of MODIS satellite imagery (250 m ×  250 m resolution, spanning 2000–2022), were extracted using site coordinates. To address potential spatial scale mismatches, we validated EVI against ground-based aboveground biomass data, confirming its reliability as a proxy for ecosystem productivity (Fig. S23). Long-term satellite-derived EVI provides a practical and standardized tool for capturing interannual vegetation dynamics across multiple sites, given the general lack of temporally replicated biomass measurements in the field. However, the biomass–EVI relationship is weaker in the alpine grassland, particularly at high biomass levels (Fig. S23). This limitation may introduce additional uncertainty and reduce predictive power in high-biomass alpine communities, and we cannot exclude the possibility that it influences estimated stability metrics. However, any such effects are more likely to increase noise rather than systematically bias stability patterns. Future work should directly quantify ecosystem stability using temporally repeated community and productivity surveys, which would allow more rigorous validation of remote-sensing proxies and strengthen inference on stability mechanisms.

Resistance and resilience were quantified for each site in response to hydrological extremes identified by SPEI (see “Identifying climate extremes”). For each qualifying extreme year *t*, we first defined a site-specific baseline productivity as the mean EVI across all normal growing seasons at that site (${\overline{{{{\rm{Productivity}}}}}}_{{{{\rm{normal}}}}}$). We then calculated resistance as the impact-normalized deviation of productivity during the extreme year from the baseline^60,62^:

1 $${\mbox{Resistance}}=1-\frac{2\,\times |{\overline{{{{\rm{Productivity}}}}}}_{{{{\rm{normal}}}}}-{{{{\rm{Productivity}}}}}_{{{{\rm{extreme}}}}}|}{{{{{\rm{Productivity}}}}}_{{{{\rm{normal}}}}}+|{{{{\rm{Productivity}}}}}_{{{{\rm{normal}}}}}-{{{{\rm{Productivity}}}}}_{{{{\rm{extreme}}}}}|}$$

and resilience as the degree of return toward baseline in the post-extreme year:

2 $$	{{\mbox{Resilience}}}\\=	\frac{2\,\times |{\overline{{{{\rm{Productivity}}}}}}_{{{{\rm{normal}}}}}-\,{{{{\rm{Productivity}}}}}_{{{{\rm{extreme}}}}}|}{\left(|{\overline{{{{\rm{Productivity}}}}}}_{{{{\rm{normal}}}}}\,-\,{{{{\rm{Productivity}}}}}_{{{{\rm{extreme}}}}}|+|{\overline{{{{\rm{Productivity}}}}}}_{{{{\rm{normal}}}}}\,-\,{{{{\rm{Productivity}}}}}_{{{{\rm{post}}}}-{{{\rm{extreme}}}}}|\right)}-1$$

where ${\overline{{{{\rm{Productivity}}}}}}_{{{{\rm{normal}}}}}$ is average ecosystem productivity across all normal growing seasons, ${{{{\rm{Productivity}}}}}_{{{{\rm{extreme}}}}}$ represents ecosystem productivity in climate-extreme years (drought or wet anomalies defined by extreme SPEI values) and ${{\mbox{Productivity}}}_{{{{\rm{post}}}}-{{{\rm{extreme}}}}}$ represents ecosystem productivity in the year following an climate-extreme year. These indices are dimensionless, bounded (−1 to 1), and scale-invariant, enabling direct comparisons of stability components across sites that differ in mean productivity; they represent a general “impact-normalized” formulation of resistance and resilience that has been widely adopted in ecological stability studies^60,63–67^. Although initially applied in studies of soil communities^62^, the underlying formulation is mathematically general and has been widely used to quantify resistance and resilience of ecological functions across ecosystems^63–67^; here we apply it to remotely sensed productivity to ensure cross-site comparability.

Because resistance and resilience are event-based metrics, robust site-level estimates require multiple qualifying extreme events. Therefore, for each site we averaged resistance and resilience across all qualifying extreme years during 2000–2022 (Text S3). This aggregation avoids over-interpreting stability from a single anomalous year and reduces the risk that a site’s inferred stability is biased upward or downward by idiosyncratic events. Finally, to address concerns about temporal alignment with the 2018 vegetation/trait survey, we repeated all calculations using the 2000–2018 period as a robustness analysis and obtained highly consistent stability estimates and trait-stability relationships (Figs. S24–S26).

### Taxonomic diversity, community-level traits, and functional diversity

Taxonomic diversity was quantified with two complementary metrics: species richness and Shannon diversity, the latter incorporating species relative abundance. For each plot, we calculated the community-weighted mean (CWM) for each trait using the equation:

3 $${CWM}=\sum {P}_{i}\times {T}_{i},$$

where$\,{P}_{i}$ represents the relative biomass of species *i*, and ${T}_{i}$ is the trait value of species *i* in the plot. Functional diversity was characterized by two widely used indices—functional dispersion (FD_is_) and Rao’s Q—to maximize comparability with previous studies^6,12^. All CWMs and diversity metrics were computed using the *vegan* package (v2.7.2)^68^.

### Statistical analyses

All statistical analyses were conducted in R (v4.5.2; R Core Team). We conducted PCA to capture key dimensions of trait variation among surveyed plant communities (*n* = 160) across temperate and alpine grasslands. Prior to analysis, all traits were standardized (mean = 0, standard deviation = 1). PCA was performed using the *PCA* function in the *FactoMineR* package (v2.12)^69^. Because multiple plots were sampled within sites, we assessed the robustness of differences between grassland types in functional composition to potential site-level non-independence in two complementary ways. First, we repeated the PCA after aggregating trait values to the site level (i.e., averaging across plots within each site), which yielded the same ordination pattern (Fig. S3). Second, we tested for differences between grassland types in functional composition using PERMANOVA (vegan, v2.7.2) on standardized trait distances, implementing a plot-level test with permutations constrained by site (site-clustered permutations) and a site-level test based on site-mean trait values (Table S3).

To compare environmental differences between temperate and alpine grasslands, we used Bayesian hierarchical models with unequal variances using a Student’s *t* distribution implemented in brms (v2.23.0). Throughout the model descriptions, we use *i* to index plots, *j* to index sites, *g* to index grassland type, and *p* to index predictors. Plot-level responses are denoted as ${y}_{{ij}}$, whereas site-level responses are denoted as ${y}_{j}$. Grassland type was consistently coded as temperate versus alpine grassland. In these models, the response variable was modeled as a function of grassland type, with site included as a random intercept and residual standard deviations allowed to differ between grassland types. For plot-level variables, such as soil properties, species diversity, species composition, and functional composition, the comparison models were written as:

4 $${y}_{{ij}} \sim \,{t}_{v}({\mu }_{{ij}},\,{\sigma }_{g}),$$

5 $${\mu }_{{ij}}=\alpha+\beta {{{{\rm{Grassland}}}}}_{j}+{b}_{j},$$

6 $${b}_{j}\, \sim {{{\rm{Normal}}}}(0,\,{\sigma }_{{{{\rm{site}}}}}),$$

where ${y}_{{ij}}$ is the response variable for plot *i* within site *j*, ${{Grassland}}_{j}$ denotes grassland type of site *j* (temperate or alpine), α is the intercept, β is the effect of grassland type, ${b}_{j}$ is the site-level random intercept, ${\sigma }_{{site}}$ is the standard deviation of site-level random effects, ${\sigma }_{g}$ is the residual standard deviation allowed to differ between grassland types, and *ν* is the degrees-of-freedom parameter of the Student’s *t* distribution.

For variables quantified at the site level (e.g., temporal stability, resistance, and resilience, as well as site-level temperature and precipitation), we analyzed the site-level dataset with one record per site. These site-level models included grassland type as the predictor and allowed residual standard deviations to differ between grassland types, without including a site random effect because each site contributed only one observation. The residual standard deviation parameter, σ, represents unexplained variation in the response after accounting for grassland type. Differences between grassland types were considered significant if the 95% Bayesian credible intervals (CIs) excluded zero. The site-level comparison models were written as:

7 $${y}_{j}\, \sim \,{t}_{v}({\mu }_{j},\,{\sigma }_{g}),$$

8 $${\mu }_{j}=\alpha+\beta {{{{\rm{Grassland}}}}}_{j},$$

where ${y}_{j}$ is the site-level response variable for site *j*, ${{Grassland}}_{j}$ denotes grassland type, α is the intercept, β is the effect of grassland type, ${\sigma }_{g}$ is the grassland-type-specific residual standard deviation, and *ν* is the degrees-of-freedom parameter. Regional differences were considered significant if the 95% Bayesian credible intervals (CIs) excluded zero.

To quantify how temporal stability, resistance, and resilience relate to temperature, precipitation, CWM traits, taxonomic diversity, and functional diversity, we fitted multivariate Bayesian models using site-level data as the primary analysis. Because temporal stability, resistance, and resilience were quantified at the site level, and because plots within the same site are not independent replicates for these temporal response variables, the main regression analyses were conducted at the site level, with one observation per site. Site-level predictor values were obtained by averaging plot-level CWM traits, taxonomic diversity, functional diversity, and soil variables within each site before model fitting. We fitted models separately for temperate and alpine grasslands to evaluate whether the predictors associated with stability components differed between the two grassland types. Given the limited number of sites within each grassland type, we used a Student’s *t* likelihood for the site-level models to reduce sensitivity to potential outliers and heavy-tailed residuals; when tail heaviness is weak, this likelihood approximates a Gaussian error structure. The primary site-level regression models were written as:

9 $${y}_{j}\, \sim \,{t}_{v}({\mu }_{j},\,\sigma ),$$

10 $${\mu }_{j}=\alpha+\mathop{\sum }_{p=1}^{P}{\beta }_{p}{X}_{{jp}},$$

where ${y}_{j}$ represents temporal stability, resistance, or resilience at site *j*; ${X}_{{jp}}$ represents standardized site-level predictors *p* for site *j*, including climate, CWM traits, taxonomic diversity, and functional diversity; *P* is the number of predictors, *α* is the intercept; ${\beta }_{p}$ are standardized regression coefficients; *σ* is the residual standard deviation; and *ν* is the degrees-of-freedom parameter of the Student’s *t* distribution.

As sensitivity analyses, we also fitted plot-level models using Gaussian residuals and pooled site-level models across both grassland types. In the pooled site-level models, we included grassland type × economic trait interactions to test whether trait effects on temporal stability, resistance, and resilience differed between temperate and alpine grasslands. These pooled interaction models followed the general form:

11 $${\mu }_{j}=\alpha+{\beta }_{1}{{{{\rm{Trait}}}}}_{j}+{\beta }_{2}{{{{\rm{Grassland}}}}}_{j}+{\beta }_{3}{{{{\rm{Trait}}}}}_{j}\times {{{{\rm{Grassland}}}}}_{j}+\mathop{\sum }_{p=1}^{P}{\gamma }_{p}{X}_{{jp}},$$

where ${{Trait}}_{j}$ represents the focal economic trait at site *j*, ${{Grassland}}_{j}$ represents grassland type, ${\beta }_{3}$ quantifies whether the effect of economic traits on temporal stability, resistance, or resilience differs between temperate and alpine grasslands, and ${X}_{{jp}}$ denotes additional standardized covariates at site *j*. These supplementary analyses yielded qualitatively similar conclusions as our site-level analyses and are presented in the Supplementary Information (Fig. S6). We presented separate site-level models for temperate and alpine grasslands in the main text, as they more directly highlight the contrasts between the two grassland types while using sites as the independent units of replication.

The plant traits analyzed included both individual traits and PCA-derived composite traits (e.g., economic traits, Fig. S27), which were derived following the approach of Craven et al.^6^. Each Bayesian model included four chains, each with 3000 iterations, including 1, 500 warm-up iterations. We used weakly informative priors for all parameters. Specifically, we assigned Normal(0, 10) priors to population-level effects (intercepts and slopes) after standardizing predictors and responses, consistent with recommended practice for weakly informative priors in ecological Bayesian analyses and recent trait-based Bayesian studies^30,70^. Standard-deviation parameters (residual SD and, where applicable, random-effect SDs) were assigned Exponential(1) priors. When correlation structures were estimated (e.g., residual or group-level correlations), we assigned LKJ(2) priors. For Student’s *t* models, we additionally specified a weakly informative prior on the degrees of freedom (*ν*). When diagnostic warnings indicated sampling problems (e.g., divergent transitions), we refitted the affected models using a higher target acceptance rate and allowing deeper NUTS trajectories. This adjustment improves the numerical stability of Hamiltonian Monte Carlo in the presence of challenging posterior geometries (e.g., strong parameter correlations or funnel-shaped regions), thereby reducing divergent transitions and yielding more reliable posterior inference. Convergence and chain mixing were assessed visually using the *bayesplot* package (v1.15.0)^71^ and numerically with the Gelman-Rubin statistic (*R̂* < 1.01)^72^. Effective sample sizes exceeded 10% of post-warm-up iterations. Posterior means and 80% and 95% Bayesian CIs were displayed using raincloud plots (implemented with tidybayes v3.0.7 and ggplot2 v4.0.1) for all fixed model parameters. Conditional effects plots, generated via the *conditional_effects* function in the *brms* package (v2.23.0), visualized the relationships between explanatory and response variables. Model fit was evaluated using posterior predictive checks and *R*^2^ from the *bayes_R*^2^ function in *brms* (v2.23.0)^73^. Parameters were considered significant if their 95% CIs did not overlap with zero.

We quantified the relative importance of climate (mean annual temperature and precipitation), CWM traits, taxonomic diversity (species richness, Shannon diversity), and functional diversity (Rao’s Q, functional dispersion) in temperate and alpine grasslands with random forests. Compared with linear or single‑tree models, random forests tolerate multicollinearity, capture complex non‑linear interactions, limit overfitting through out‑of‑bag (OOB) validation, and require no data normalization^74^. Models were fitted with rfPermute (v2.5.5), which yields permutation‑based importance scores (%IncMSE) and *P* values. The number of trees and the mtry parameter were tuned by minimizing OOB error. Effect direction was assigned as the sign of the Spearman correlation between each predictor and its partial dependence values, and verified against the sign of its permutation importance. Because we evaluated multiple traits, we additionally performed a sensitivity analysis in which we fitted univariate frequentist regressions and applied Holm correction to *P* values within each region ×  stability-component family of tests; the main results were unchanged (Fig. S28).

We assessed how climate (mean annual temperature and precipitation), soil properties (pH, bulk density, organic carbon, available N and P), species composition (annual/perennial ratio), and economic traits (PCA‑derived composite traits) influenced ecosystem temporal stability, resistance, and resilience via both direct and indirect pathways, using Bayesian structural equation models (BSEMs). Because temporal stability, resistance, and resilience were quantified at the site level, the primary BSEMs were fitted using site-level data, with one observation per site. Site-level values of plot-based predictors, including soil properties, species composition, and economic traits, were obtained by averaging plot-level values within each site before model fitting. Given the limited number of sites within each grassland type, we fitted pooled site-level BSEMs across temperate and alpine grasslands, rather than separate grassland-specific BSEMs, to improve inferential stability. In these pooled models, growing-season temperature was used to represent ecosystem context because it captured the major environmental contrast between the two grassland types (Table S8) and provided a continuous proxy for this gradient (Fig. S2). We therefore tested biome-dependent using trait × temperature interactions. We did not additionally include grassland type or grassland type × trait interactions in the main BSEMs because grassland type was closely aligned with the temperature gradient already included in the models; including both would have introduced largely redundant predictors, increased model complexity, and potentially increased collinearity under the limited site-level sample size. To further avoid overparameterization, we used a priori variable selection in the main BSEMs, retaining LDMC as the focal economic trait because it is widely used in trait-stability research^20,21^, growing-season temperature as the focal climatic driver, and soil pH and species composition as key abiotic and biotic covariates, respectively. Plot-level BSEMs and BSEMs including the broader predictor set were retained as supplementary sensitivity analyses and are reported in the Supplementary Information (Figs. S10 and S11). For each endogenous variable in the BSEM, we fitted a Bayesian regression submodel of the general form:

12 $${z}_{j}\, \sim \,{t}_{v}({\mu }_{j},\,\sigma ),$$

13 $${\mu }_{j}=\alpha+\mathop{\sum }_{p=1}^{P}{\beta }_{p}{X}_{{jp}},$$

where ${z}_{j}$ represents an endogenous variable in the site-level path model for site *j*; ${X}_{{jp}}$ represents direct predictor *p* specified by the hypothesized causal structure, including the LDMC ×  temperature interaction where relevant, *α* is the intercept, ${\beta }_{p}$ are standardized path coefficients, *σ* is the residual standard deviation, and *ν* is the degrees-of-freedom parameter. Predictor collinearity was evaluated with pairwise correlations and variance‑inflation factors (VIFs, *performance v0.15.3*^75^). Precipitation was excluded because it was strongly correlated with temperature and soil organic carbon in alpine grasslands (*|r*| > 0.7; VIF ≈ 10); all remaining VIFs were <5. We then fitted the BSEMs using the “*brms*” package (v2.23.0), applying the same priors, warm-up settings, sampling parameters, model configurations, and validation procedures as in our previous analyses. Because Fisher’s C and d-separation tests are not directly provided by the Bayesian SEM framework implemented in brms, we additionally evaluated the implied conditional independencies using the frequentist piecewiseSEM framework as a complementary diagnostic check. These diagnostics were used to assess the consistency between the hypothesized path structure and the observed covariance structure, whereas parameter estimates and inference were based on the Bayesian models. Pathways in the BSEMs were considered significant if their 95% CIs did not include zero.

### Reporting summary

Further information on research design is available in the Nature Portfolio Reporting Summary linked to this article.

## Supplementary information

Supplementary Information

Reporting Summary

Transparent Peer Review File

## Source data

Source Data

## Data Availability

The plant community and trait data generated in this study, the derived data underlying the figures, and the associated analysis materials have been deposited in Figshare at https://doi.org/10.6084/m9.figshare.27895827. CRU TS v4.09 climate data^76,77^ are available from the NERC EDS Centre for Environmental Data Analysis at https://catalogue.ceda.ac.uk/uuid/9cf07e92afaa405da4f40b6733f362d3/. SPEIbase v2.11 data^78–80^ are available from the Spanish National Research Council at https://spei.csic.es/spei_database_2_11/. Monthly potential evapotranspiration data from the Global Aridity Index and Potential Evapotranspiration (ET0) Database v3.1^81,82^ are available at https://doi.org/10.6084/m9.figshare.7504448.v7. MODIS/Terra MOD13Q1 Version 6.1 vegetation-index data [ref. ^83^] are available from NASA’s Land Processes Distributed Active Archive Center at https://doi.org/10.5067/MODIS/MOD13Q1.061. Source data are provided with this paper.

## Code availability

The custom R code used for data processing, statistical analyses, and figure generation is available in Figshare at https://doi.org/10.6084/m9.figshare.27895827.
