# Supplementary material for: Contrasting plant trait strategies underpin ecosystem stability in temperate and alpine grasslands
Source: Nat Commun. 2026 Oct 8;17:11435. doi: 10.1038/s41467-026-78537-2 (PMC13737107; doi:10.1038/s41467-026-78537-2)
Supplement: Supplementary file 1 [file 41467_2026_78537_MOESM1_ESM.pdf]

Supplementary Information for

Contrasting plant trait strategies underpin ecosystem stability in temperate and alpine grasslands

This file includes:

1) Supplementary Notes 1–5

2) Supplementary Tables 1–8

3) Supplementary Figures 1–28

4) Supplementary References

Supplementary Note 1: Independent-dataset validation of trait–stability relationships in temperate and alpine grasslands

To address the limited site-level replication in our main survey (n = 10 per biome) and to evaluate the generality and robustness of our findings, we conducted an external validation using two independent transect datasets with increased site numbers (temperate: n = 35 sites; alpine: n = 33 sites; see Figure S11). These transects are independent of the main survey but span comparable regions and vegetation types. Species-composition data were taken from the published sources [1,2], and species' trait values were matched to our standardized, field-measured trait database (2018). Following the identical preprocessing and analytical workflow described in the main Methods, we computed community-weighted mean (CWM) traits and quantified temporal stability from MODIS EVI time series (2000–2022).

Supplementary Note 2: Plant trait sampling and measurement protocols

We quantified plant functional strategies using a trait dataset that we compiled during the 2018 field campaign, following trait-specific standardized protocols. We provide full procedural details here to ensure reproducibility. In total, we measured 15 morphological, chemical, and stomatal traits for 343 vascular plant species (Table S2), including plant height (cm), leaf area (LA, cm^2), specific leaf area (SLA, $cm^2\ g^{-1}$), leaf dry matter content (LDMC, %), stem dry matter content (SDMC, %), root dry matter content (RDMC, %), leaf carbon concentration (Leaf C, %), leaf nitrogen concentration (Leaf N, %), leaf phosphorus concentration (Leaf P, mg g^{-1}), root nitrogen concentration (Root N, %), root phosphorus concentration (Root P, mg g^{-1}), stomatal length (μm), stomatal width (μm), stomatal density (no. mm^{-2}), and stomatal area (μm^2; derived). Plant height was measured in situ prior to harvest as the distance from the soil surface to the highest photosynthetic tissue (excluding inflorescences when applicable); for each species within a plot we measured 3–5 healthy individuals (depending on local abundance) and used the mean as the plot-level species value. For leaf morphological traits (LA, SLA, LDMC), we collected 6–10 fully expanded, healthy leaves per species (pooled across individuals when necessary), scanned them using a portable flatbed scanner (CanoScan LiDE 110, Japan), and quantified LA with ImageJ; leaves were then rehydrated to saturated mass by immersion in deionized water for 24 h at room temperature, gently blotted, weighed to obtain saturated fresh mass, oven-dried at 60 °C for 48 h, and reweighed to obtain dry mass. LDMC (%) was calculated as 100 × (dry mass / saturated fresh mass), and SLA ($cm^2\ g^{-1}$) as LA / dry mass. For SDMC and RDMC, stem segments (main stem/culm tissue) and fine roots were sampled from 3–5 individuals per species when possible, cleaned of debris, brought to saturated fresh mass by soaking in deionized water until fully hydrated, blotted, weighed for saturated fresh mass, and then oven-dried at 60 °C for 48 h to determine dry mass; SDMC (%) and RDMC (%) were calculated as 100 × (dry mass / saturated fresh mass) for stem and root tissues, respectively. For elemental traits, dried

leaf and root samples were ground to a fine powder using an agate mortar grinder; Leaf C (%) and Leaf N (%) and Root N (%) were measured using an elemental analyzer (Vario MAX CN, Elementar, Germany). For phosphorus, powdered samples were digested with concentrated nitric acid (68% HNO_3) using a microwave digestion system, and Leaf P and Root P (mg g^{-1}) were quantified by inductively coupled plasma optical emission spectrometry (ICP-OES). For stomatal traits, three leaf subsamples (replicates) per species were prepared; each replicate was imaged on both adaxial and abaxial surfaces when distinguishable, using a scanning electron microscope (S-3400N, Hitachi, Japan). Stomatal length and width were measured from calibrated micrographs, stomatal density was computed as the number of stomata per unit leaf area (mm^2) across multiple fields of view per replicate, and stomatal area was derived assuming an elliptical geometry: stomatal area = stomatal length × stomatal width × (π/4). When both leaf surfaces were measured, stomatal metrics were averaged across surfaces to yield a single species value. Species-level trait values were summarized as means of replicate measurements and then used in downstream community calculations (e.g., community-weighted means and functional diversity metrics; see Statistical Analyses). Because trait sampling was conducted in a single year (2018) to maintain consistent field and laboratory processing across the broad transect, these measurements primarily capture spatial (among-site) variation in trait expression and may not fully reflect interannual trait plasticity; we therefore acknowledge this as a limitation and discuss its implications for interpreting trait–stability relationships in the Discussion.

Supplementary Note 3: Soil sampling and measurement of soil properties

To quantify soil properties, we collected soil samples from the 0–10 cm layer in each plot immediately after the vegetation survey. Samples were air-dried and sieved through a 2-mm mesh prior to analysis. Soil organic carbon (SOC) was determined using the H_2SO_4–$K_2Cr_2O_7$ oxidation method [3]. Soil available phosphorus was measured using Olsen's method [4]. Soil available nitrogen (defined as the sum of ammonium, nitrate, and dissolved organic N) was quantified using a TOC–TN analyzer (Shimadzu, Kyoto, Japan). Soil pH was measured in a soil–water suspension (1:2.5, w/v) using an Ultrameter II 6PFCE (Myron L Company, USA). Soil bulk density (BD) was determined using the volumetric ring method.

Supplementary Note 4: Determination of growing-season length and derivation of growing-season climate variables

High-resolution monthly climate data (CRU TS v4.03) were obtained at 0.5° spatial resolution for 2000–2022 from the CRU data portal (https://crudata.uea.ac.uk/cru/data/hrg/). Monthly potential evapotranspiration (PET) was obtained from the Global Potential Evapo-Transpiration Climate Database (http://www.csi.cgiar.org). We calculated a monthly moisture index (MI) as the ratio of monthly precipitation to PET to represent monthly water balance at each site. Consecutive months were classified as part of the plant growing season if they met both criteria: (1) mean monthly temperature ⩾ 5 °C and (2) MI ⩾ 0.05 [5,6]. Growing-season length was defined as the number of months satisfying these criteria. Growing-season temperature was calculated as the mean temperature across all growing-season months, and growing-season precipitation was calculated as the cumulative precipitation across the same months.

Supplementary Note 5: Identification of extreme climate events and the calculation of resistance and resilience

At each study site, we classified growing seasons as dry, normal, or wet based on their standardized precipitation–evapotranspiration index (SPEI) values, using a threshold of ±0.67 times the standard deviation (SD). Growing seasons with SPEI $\leqslant$ −0.67 SD were considered dry (approximately at or below the 25th percentile), and those with SPEI $\geqslant$ 0.67 SD were considered wet (approximately at or above the 75th percentile). Seasons with SPEI values between −0.67 and 0.67 SD were regarded as normal. Adopting these thresholds aligns with established research conventions [7-9], ensuring both comparability and consistency among studies. Moreover, this classification yielded a sufficient number of dry and wet seasons across all sites, thereby increasing the statistical robustness of our analyses.

To calculate resistance and resilience reliably, we imposed specific criteria for handling consecutive extreme events. If two consecutive extreme seasons were of different types (e.g., a dry season followed by a wet season), we excluded the earlier season from the resilience calculation and the subsequent one from both resistance and resilience calculations. When two or more consecutive extreme seasons were of the same type (e.g., consecutive wet seasons), we calculated resilience only for the later season, and only if it was followed by a normal season. For sites experiencing multiple qualifying extreme events between 2000 and 2022, we averaged all corresponding resistance and resilience values to derive a single representative measure. This approach provides a robust overall indicator of each site's mean resistance and resilience, an approach that is especially suitable given that community structure was surveyed only once (i.e., cross-sectionally). It also aligns well with the data structure used in our temporal stability metrics.

Supplementary Table 1. List of 15 plant traits in this study

TraitName	Category	VarName	UnitName
Leaf carbon concentration	Chemical	Leaf C	%
Leaf nitrogen concentration	Chemical	Leaf N	%
Leaf phosphorus concentration	Chemical	Leaf P	mg g^{-1}
Leaf area	Morphological	LA	cm^2
Leaf dry mass content	Morphological	LDMC	%
Plant height	Morphological	Height	cm
Specific leaf area	Morphological	SLA	cm^2 g^{-1}
Stem dry mass content	Morphological	SDMC	%
Root nitrogen concentration	Chemical	Root N	%
Root phosphorus concentration	Chemical	Root P	mg g^{-1}
Root dry mass content	Morphological	RDMC	%
Stomatal area	Morphological	Stomatal area	μm^2
Stomatal length	Morphological	Stomatal length	μm
Stomatal width	Morphological	Stomatal width	μm
Stomatal density	Morphological	Stomatal density	stomata/mm^2

Supplementary Table 2. Summary table comparing abiotic and biotic factors between temperate and alpine grasslands using Bayesian estimation. A positive estimate indicates that the value is higher in temperate grasslands compared to alpine grasslands, whereas a negative estimate indicates higher value in alpine grasslands. Significant differences are indicated when the 95% credible interval (CI) does not include zero; otherwise, the difference is not considered significant.

Trait	Model type	N	Estimate	CI_{lower}	CI_{upper}	Significant
Species richness	Hierarchical	160	-0.2	-1.23	0.81	Not Significant
**Temperature**	**Site-level**	**20**	**-8.15**	**-9.74**	**-6.56**	**Significant**
**Precipitation**	**Site-level**	**20**	**2.14**	**-151.03**	**155.31**	**Not Significant**
**Soil properties**	**Hierarchical**	**160**	**0.33**	**-1.12**	**1.78**	**Not Significant**
**Temporal stability**	**Site-level**	**20**	**5.07**	**1.59**	**8.59**	**Significant**
**Resistance**	**Site-level**	**20**	**0.06**	**0**	**0.12**	**Significant**
**Resilience**	**Site-level**	**20**	**0.02**	**-0.3**	**0.33**	**Not Significant**
Height	Hierarchical	160	-11.21	-19.43	-2.04	Significant
LA	Hierarchical	160	-1.73	-3.67	0.21	Not Significant
SLA	Hierarchical	160	-0.88	-19.41	18.52	Not Significant
LDMC	Hierarchical	160	-3.77	-14.79	6.73	Not Significant
SDMC	Hierarchical	160	1.69	-6.56	9.92	Not Significant
RDMC	Hierarchical	160	-5.48	-14.46	3.66	Not Significant
Leaf C	Hierarchical	160	-1.12	-3.81	1.58	Not Significant
Leaf N	Hierarchical	160	-0.27	-0.94	0.44	Not Significant
Leaf P	Hierarchical	160	-0.03	-18.93	18.21	Not Significant
Root N	Hierarchical	160	-0.11	-0.37	0.16	Not Significant
Root P	Hierarchical	160	0.26	-20.07	20.19	Not Significant
SDdensity	Hierarchical	160	0.83	-17.97	19.8	Not Significant
SDlen	Hierarchical	160	-10.26	-14.71	-5.86	Significant
SDw	Hierarchical	160	-7.72	-10.12	-5.37	Significant
SDarea	Hierarchical	160	-1.93	-21.4	17.51	Not Significant
APratio	Hierarchical	160	-0.17	-0.3	-0.03	Significant
Economic traits	Hierarchical	160	-0.39	-2.5	1.81	Not Significant
Size traits	Hierarchical	160	-2.49	-3.5	-1.52	Significant

All models used a Student's t likelihood and allowed unequal variances between regions (σ ~ Region).

Supplementary Table 3. Regional differences in multivariate trait composition between temperate and alpine grasslands assessed by PERMANOVA under alternative permutation schemes.

Method	n_plots	n_sites	n_perm	F_obs	R^2	*P*-value
PERMANOVA (plot level; free permutations)	160	20	9999	29.18	0.160	0.0001
PERMANOVA (plot level; clustered permutations by site)	160	20	9999	29.20	0.156	0.003
PERMANOVA (site level; free permutations)	NA	20	9999	4.22	0.189	0.0035

PERMANOVA was performed on Euclidean distances calculated from z-standardized trait matrices. To account for potential non-independence among plots within sites, we report a plot-level test using site-clustered permutations and a site-level test based on site-mean trait values. All PERMANOVA tests were one-sided upper-tail permutation tests based on 9,999 permutations. Values shown are the observed pseudo-F statistic (F_obs), variance explained (R^2), and permutation ***P***-values (***P***_value; 9,999 permutations). Method (PERMANOVA scheme), n_plots (number of plots), n_sites (number of sites), n_perm (number of permutations), F_obs (observed pseudo-F), R^2_obs (variance explained), ***P***_value (permutation ***P***-value).

Supplementary Table 4. Summary table of species composition metrics based on annual and perennial plants in temperate and alpine grasslands. Values represent the mean ± standard error (SE) and median for each metric, based on plot-level measurements. Metrics include the species richness of annual and perennial plants, the richness ratio of annuals to perennials, and the biomass ratio of annuals to perennials. Estimates are derived from standardized field surveys conducted across 160 plots in 2018. These comparisons highlight differences in life-history strategies between the two grassland types.

Grassland type	Metric	Mean ± SE	Median
Temperate grassland	Perennial species richness	7.95 ± 0.31	8
	Annual species richness	1.98 ± 0.19	2
	Annual:Perennial richness ratio	**0.27 ± 0.02**	**0.27**
	Annual:Perennial biomass ratio	**0.32 ± 0.06**	**0.07**
Alpine grassland	Perennial species richness	8.40 ± 0.56	8
	Annual species richness	0.82 ± 0.11	1
	Annual:Perennial richness ratio	**0.09 ± 0.01**	**0.09**
	Annual:Perennial biomass ratio	**0.07 ± 0.03**	**0**

Supplementary Table 5. Convergence and sampling diagnostics for plot- and site-level multivariate Bayesian models quantifying relationships between three stability components (temporal stability, resistance, and resilience) and economic traits. We report standardized posterior mean effects of the economic trait predictor (β; mean [95% credible interval]) for each Scale (Plot vs Site) × Region × stability component combination, together with Markov chain convergence and sampling efficiency metrics (Rhat, Bulk_ESS, Tail_ESS). Effect estimates were quantitatively consistent between plot- and site-level analyses; however, credible intervals were generally wider at the site level due to the smaller sample size (n = 10 per grassland type).

Level	Region	Response	Est_ci	Rhat	Bulk_ESS	Tail_ESS
Plot	Temperate grassland	Temporal stability	0.65 [0.50, 0.79]	1.00	4313	3455
Plot	Temperate grassland	Resistance	0.33 [0.16, 0.51]	1.00	4004	4015
Plot	Temperate grassland	Resilience	0.55 [0.39, 0.70]	1.00	5393	4462
Plot	Alpine grassland	Temporal stability	-0.57 [-0.73, -0.43]	1.00	5157	4724
Plot	Alpine grassland	Resistance	-0.70 [-0.83, -0.56]	1.00	4583	4133
Plot	Alpine grassland	Resilience	0.59 [0.44, 0.74]	1.00	5367	4490
Site	Temperate grassland	Temporal stability	0.79 [0.28, 1.28]	1.00	4483	3564
Site	Temperate grassland	Resistance	0.65 [0, 1.27]	1.00	4957	3635
Site	Temperate grassland	Resilience	0.67 [0.12, 1.25]	1.00	4838	3562
Site	Alpine grassland	Temporal stability	-0.58 [-1.17, -0.04]	1.00	3001	2321
Site	Alpine grassland	Resistance	-0.82 [-1.31, -0.37]	1.00	4656	3344
Site	Alpine grassland	Resilience	0.83 [0.38, 1.29]	1.00	3887	3355

Note: ***β*** values are standardized posterior means with 95% credible intervals (CrI). All rows report the effect of the economic trait axis. Rhat values close to 1.00 and large effective sample sizes (Bulk_ESS and Tail_ESS) indicate satisfactory convergence and mixing. Plot-level models used a Gaussian likelihood, whereas site-level models used a Student's t likelihood to improve robustness under small sample size. Asterisks denote effects whose 95% credible intervals exclude zero. Higher values of the economic trait axis indicate more acquisitive (fast) strategies (lower values indicate more conservative strategies).

Supplementary Table 6. Posterior summaries for the grassland type × economic trait interaction term from multivariate Bayesian models at plot and site levels. The table reports the estimated interaction effects on temporal stability, resistance, and resilience, including posterior mean (Estimate), posterior SD (Est.Error), 95% credible intervals (l-95% CI, u-95% CI), and convergence/ESS diagnostics (Rhat, Bulk_ESS, Tail_ESS).

Model	Term	Estimate	l-95% CI	u-95% CI	Rhat	Bulk_ESS	Tail_ESS
Plot	Temporal stability	-4.6	-5.77	-3.44	1.00	3451	3968
Plot	Resistance	-0.07	-0.09	-0.06	1.00	3020	4250
Plot	Resilience	0.16	0.07	0.25	1.00	3486	4190
Site	Temporal stability	-4.49	-8.04	-1.68	1.00	4917	3821
Site	Resistance	-0.09	-0.15	-0.03	1.00	6210	3848
Site	Resilience	0.24	-0.07	0.56	1.00	6040	4346

Supplementary Table 7. Posterior summaries of focal structural paths from Bayesian structural equation models (BSEMs) relating climate, soil properties, and plant economic traits to ecosystem temporal stability, resistance, and resilience in temperate and alpine grasslands. For brevity and space constraints, only focal (hypothesis-driven) structural paths are shown.

Region	Response	Term	Est_ci	Rhat	Bulk_ESS	Tail_ESS
Temperate grassland	Resilience	P_mean	0.15 [-0.19, 0.49]	1.00	4485	4176
Temperate grassland	Resilience	T_mean	-0.74 [-1.05, -0.43]*	1.00	5012	4490
Temperate grassland	Resilience	Economic trait	0.53 [0.26, 0.80]*	1.00	5177	4067
Temperate grassland	Resilience	soilBD	-0.20 [-0.41, 0.00]	1.00	7174	4105
Temperate grassland	Resilience	soilC	-0.43 [-0.78, -0.07]*	1.00	4676	4106
Temperate grassland	Resilience	soilN	-0.32 [-0.51, -0.13]*	1.00	7205	4635
Temperate grassland	Resilience	soilP	0.09 [-0.10, 0.29]	1.00	7515	4982
Temperate grassland	Resilience	soilpH	0.43 [0.25, 0.60]*	1.00	6754	4604
Temperate grassland	Resistance	P_mean	0.35 [-0.05, 0.74]	1.00	3282	4214
Temperate grassland	Resistance	T_mean	-0.11 [-0.47, 0.24]	1.00	4286	3870
Temperate grassland	Resistance	Economic trait	0.46 [0.14, 0.77]*	1.00	5021	4718
Temperate grassland	Resistance	soilBD	0.24 [-0.01, 0.49]	1.00	7584	4814
Temperate grassland	Resistance	soilC	-0.49 [-0.89, -0.09]*	1.00	3475	4092
Temperate grassland	Resistance	soilN	0.63 [0.40, 0.85]*	1.00	8325	4760
Temperate grassland	Resistance	soilP	-0.27 [-0.50, -0.04]*	1.00	8414	4971
Temperate grassland	Resistance	soilpH	-0.16 [-0.37, 0.06]	1.00	7402	4617
Temperate grassland	Temporal stability	P_mean	-0.10 [-0.36, 0.15]	1.00	4016	4134
Temperate grassland	Temporal stability	T_mean	0.47 [0.24, 0.72]*	1.00	3889	3960
Temperate grassland	Temporal stability	Economic trait	0.36 [0.16, 0.56]*	1.00	5387	4656
Temperate grassland	Temporal stability	soilBD	0.14 [-0.02, 0.30]	1.00	8701	4671
Temperate grassland	Temporal stability	soilC	-0.20 [-0.47, 0.05]	1.00	3857	4288
Temperate grassland	Temporal stability	soilN	0.32 [0.18, 0.46]*	1.00	8228	4700
Temperate grassland	Temporal stability	soilP	-0.22 [-0.37, -0.07]*	1.00	8016	4639
Temperate grassland	Temporal stability	soilpH	-0.21 [-0.35, -0.07]*	1.00	7259	4403
Alpine grassland	Resilience	T_mean	-0.22 [-0.52, 0.09]	1.00	9833	4815
Alpine grassland	Resilience	Economic trait	0.58 [0.36, 0.81]*	1.00	8951	4453
Alpine grassland	Resilience	soilBD	0.11 [-0.15, 0.36]	1.00	9598	4353
Alpine grassland	Resilience	soilC	0.16 [-0.25, 0.57]	1.00	9374	4324
Alpine grassland	Resilience	soilN	-0.14 [-0.41, 0.13]	1.00	8404	4267
Alpine grassland	Resilience	soilP	-0.07 [-0.27, 0.14]	1.00	8470	4268
Alpine grassland	Resilience	soilpH	0.04 [-0.35, 0.43]	1.00	7597	4337
Alpine grassland	Resistance	T_mean	0.08 [-0.19, 0.35]	1.00	9288	4562
Alpine grassland	Resistance	Economic trait	-0.65 [-0.84, -0.46]*	1.00	10119	4667
Alpine grassland	Resistance	soilBD	-0.01 [-0.24, 0.22]	1.00	10137	4628

Alpine grassland	Resistance	soilC	-0.34 [-0.69, 0.02]	1.00	8698	4633
Alpine grassland	Resistance	soilN	0.25 [0.01, 0.49]*	1.00	9258	4718
Alpine grassland	Resistance	soilP	-0.04 [-0.22, 0.14]	1.00	9595	4506
Alpine grassland	Resistance	soilpH	-0.20 [-0.54, 0.14]	1.00	7817	4428
Alpine grassland	Temporal stability	T_mean	-0.65 [-0.89, -0.42]*	1.00	6860	4364
Alpine grassland	Temporal stability	Economic trait	-0.48 [-0.65, -0.30]*	1.00	7767	4862
Alpine grassland	Temporal stability	soilBD	0.11 [-0.10, 0.32]	1.00	8464	4057
Alpine grassland	Temporal stability	soilC	-0.33 [-0.65, -0.01]*	1.00	7063	4354
Alpine grassland	Temporal stability	soilN	0.18 [-0.04, 0.40]	1.00	6826	4269
Alpine grassland	Temporal stability	soilP	-0.10 [-0.27, 0.06]	1.00	7942	4578
Alpine grassland	Temporal stability	soilpH	0.44 [0.13, 0.75]*	1.00	7452	4510

Entries report posterior means with 95% credible intervals (CrI) for direct effects on each response variable. Economic trait denotes the PCA-derived plant economic trait composite used as the focal functional-strategy predictor. T_mean and P_mean denote mean temperature and precipitation, respectively. soilBD, soilpH, soilC, soilN, and soilP denote soil bulk density, pH, soil organic carbon, available nitrogen, and available phosphorus, respectively (see Methods for details). Parameters were considered supported when the 95% credible interval excluded zero. All models were fitted in brms with 4 chains, 3,000 iterations (1,500 warm-up). When diagnostic warnings indicated sampling problems (e.g., divergent transitions), we increased the NUTS target acceptance rate and tree depth (adapt_delta = 0.95; max_treedepth = 12). Convergence diagnostics ($\hat{R}$) and effective sample sizes (Bulk_ESS, Tail_ESS) are reported for each parameter. In alpine grasslands, precipitation (P_mean) was excluded where necessary due to strong collinearity with other predictors (see collinearity screening in Methods); therefore, P_mean does not appear for the alpine models.

Supplementary Table 8. Geographic and climatic (including annual and growing season [GS]) information for sampling sites in our study temperate and alpine grasslands.

Ecosystem types	ID	Longitude (°E)	Latitude (°N)	Elevation (m)	Aridity	Temperature (℃)		Precipitation (mm)	
						annual	GS	annual	GS
Temperate grassland	01	123.51	44.59	144	0.68	5.10	16.60	425	410
Temperate grassland	02	121.04	44.52	269	0.73	5.80	16.66	393	378
Temperate grassland	03	120.33	45.11	660	0.71	3.72	13.60	387	372
Temperate grassland	04	118.36	44.77	1019	0.71	0.56	12.03	345	320
Temperate grassland	05	116.52	44.26	1129	0.77	1.17	12.27	283	267
Temperate grassland	06	116.67	43.55	1272	0.73	0.16	11.74	321	304
Temperate grassland	07	117.68	44.51	1024	0.73	1.96	12.10	319	298
Temperate grassland	08	114.89	44.01	1101	0.83	0.10	12.94	228	219
Temperate grassland	09	113.50	43.84	1022	0.86	2.47	14.20	199	190
Temperate grassland	10	112.15	43.63	955	0.88	3.69	14.87	183	169
Alpine grassland	01	95.45	31.46	4104	0.4	0.41	5.7	606	572
Alpine grassland	02	93.53	31.85	4509	0.37	-1.5	3.14	593	560
Alpine grassland	03	92.01	31.64	4587	0.61	-4.37	4.4	430	414
Alpine grassland	04	90.74	31.38	4617	0.65	-6.76	5.89	426	414
Alpine grassland	05	89.72	31.54	4588	0.67	-3.06	6.93	426	412
Alpine grassland	06	87.82	31.87	4570	0.79	-2.57	6.77	286	261
Alpine grassland	07	85.84	31.92	4938	0.9	-3.77	3.74	125	95
Alpine grassland	08	83.34	32.41	4578	0.94	-3.9	5.71	75	62
Alpine grassland	09	81.23	32.3	4558	0.92	-3.49	5.29	102	89
Alpine grassland	10	80.15	32.48	4328	0.93	-1.27	6.73	89	78

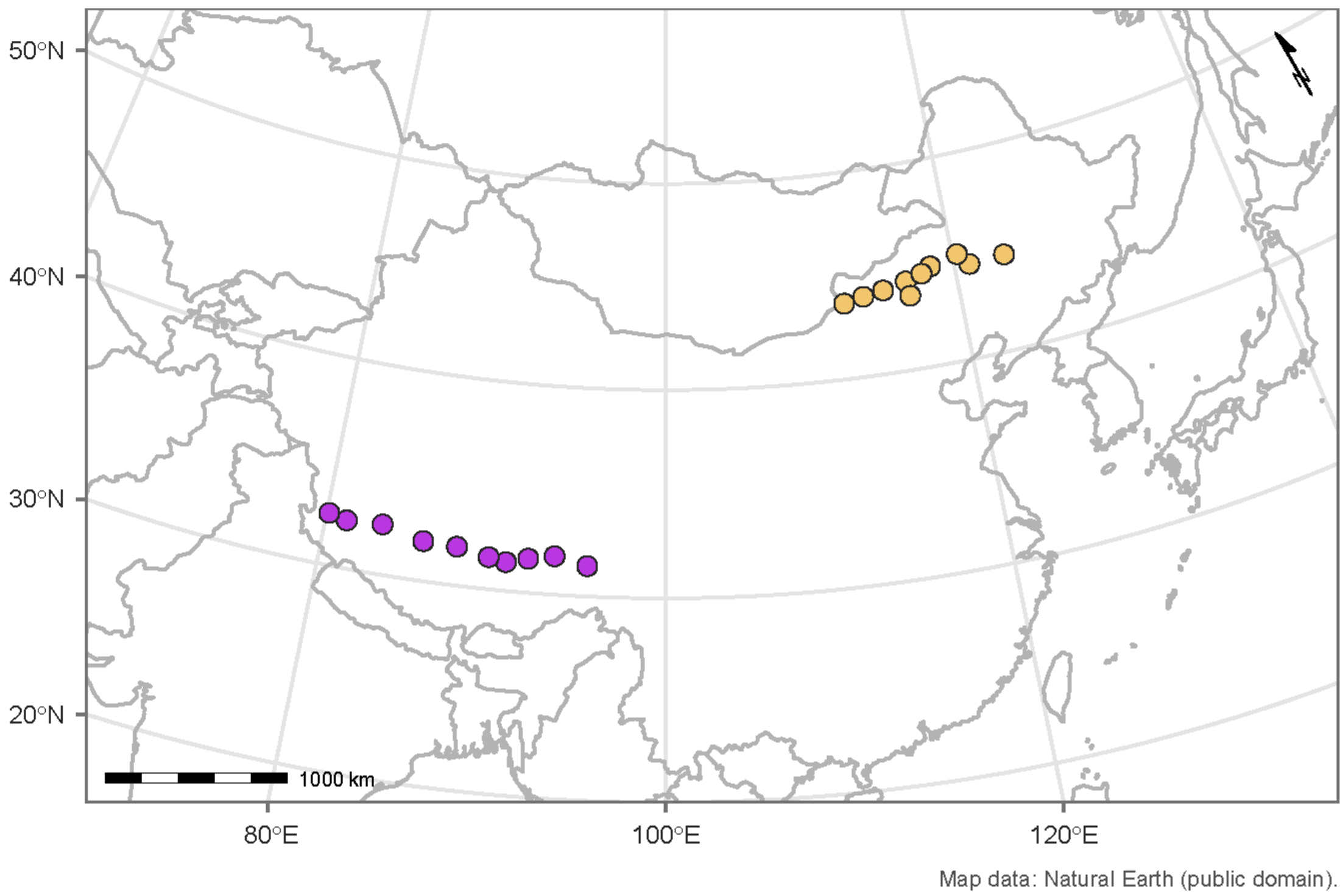

Figure S1. Geographic distribution of the sampling sites. Yellow and purple circles indicate temperate and alpine grassland sampling sites, respectively. The map was created by the authors in R using the sf and ggplot2 packages. Country boundaries and coastlines were obtained from Natural Earth (public domain; https://www.naturalearthdata.com/about/terms-of-use/) through the rnaturalearth package.

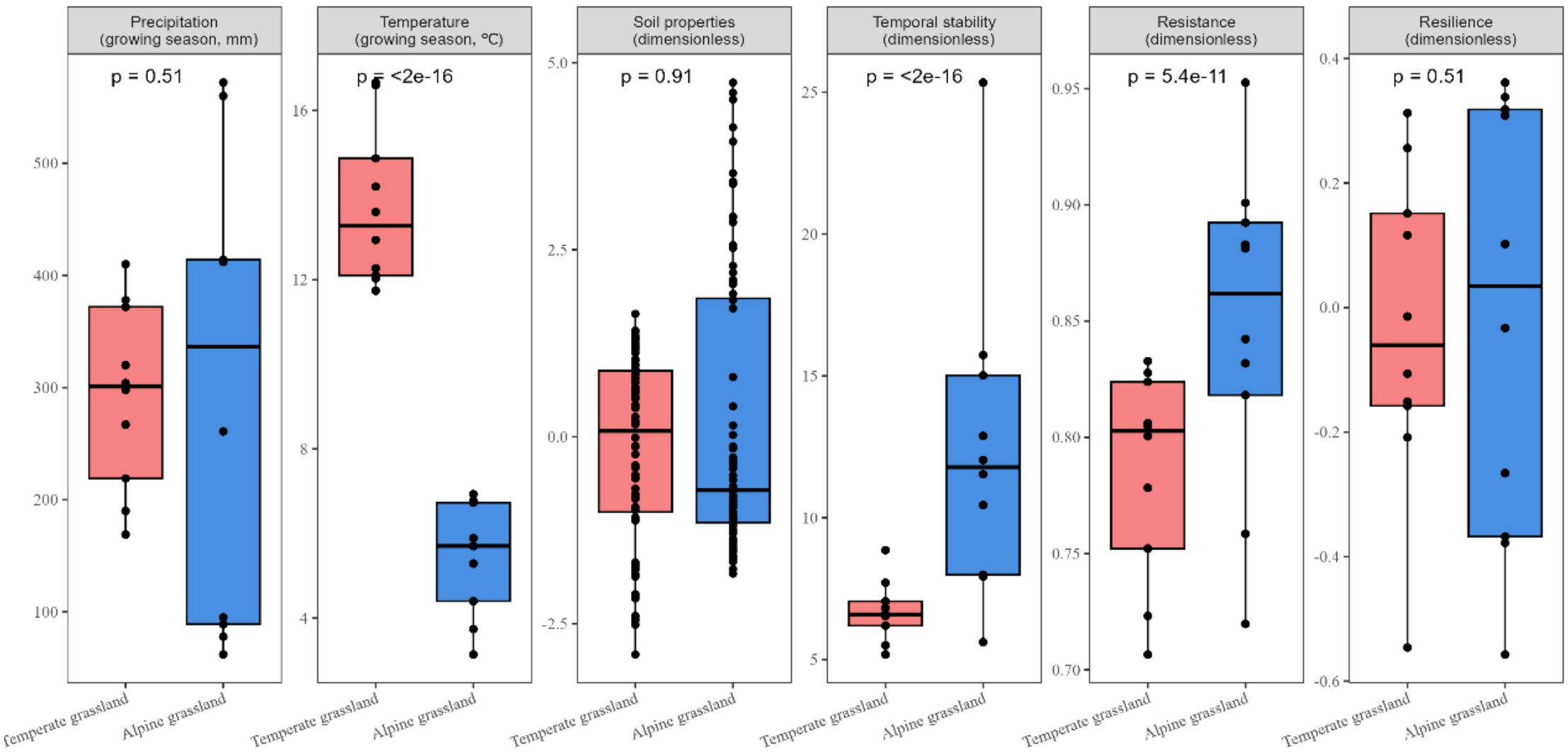

Figure S2 Comparison of abiotic factors (precipitation, temperature, and soil properties) and ecosystem stability (temporal stability, resistance and resilience) between temperate and alpine grasslands. Boxplots show the interquartile range (IQR) within the box, with whiskers extending to either the minimum value or 1.5 times the IQR, whichever is more extreme, and the median indicated by a horizontal line. Soil properties are represented by the first principal component (PC1), based on principal component analysis of soil pH, bulk density, organic carbon, available nitrogen, and phosphorus. PC1 is positively correlated with organic carbon, available nitrogen, and phosphorus, and negatively correlated with soil pH and bulk density. Group differences in this figure were evaluated using frequentist tests for simplicity, and the results are consistent with the robust Bayesian comparisons reported in Table S2.

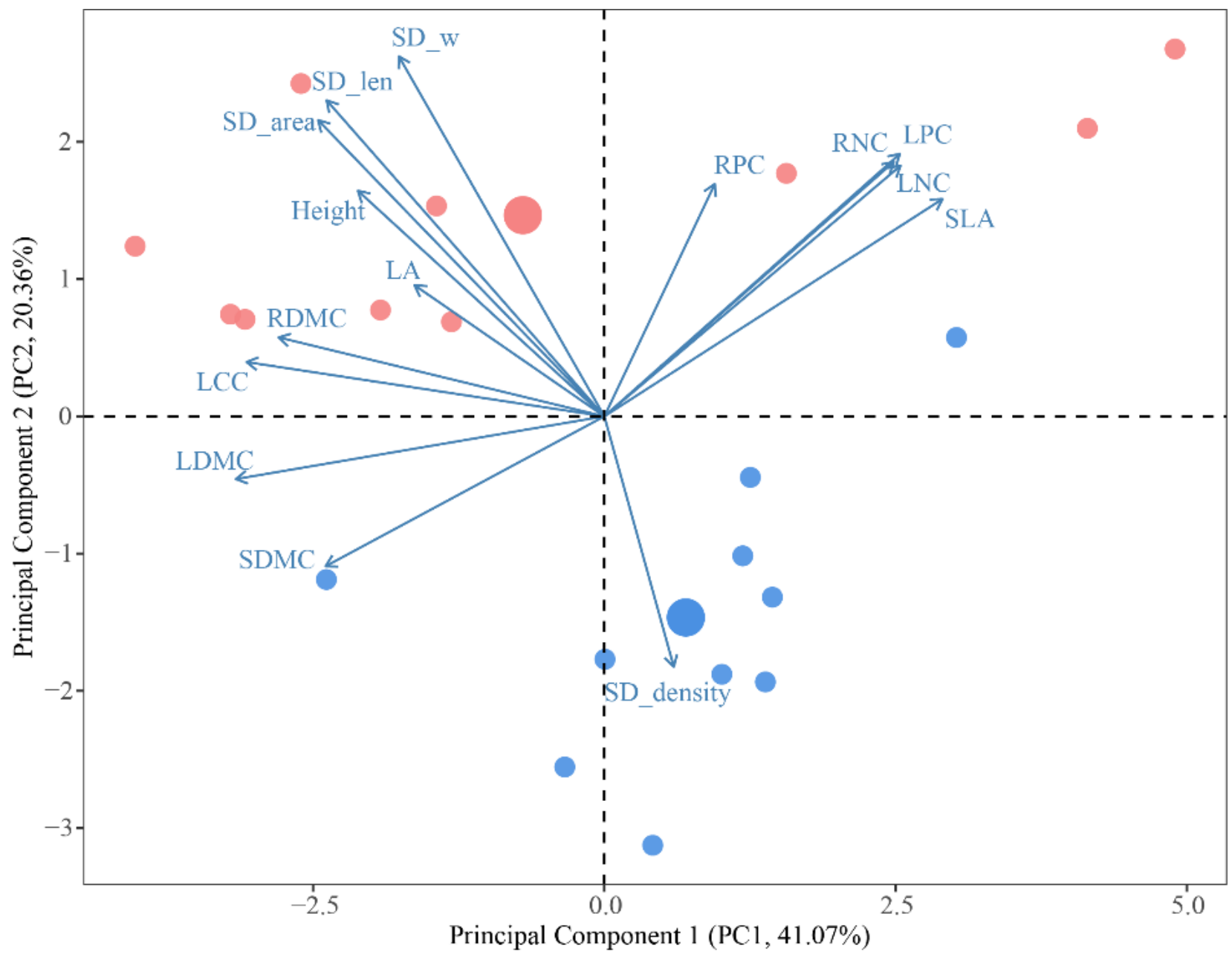

Figure S3. Site-level trait space captured by PCA (n = 20 sites). Principal component analysis (PCA) was performed on community-weighted mean values of 15 plant functional traits, aggregated to the site level by averaging across plots within each site (temperate and alpine grasslands combined). Traits included leaf carbon (Leaf C, %), leaf nitrogen (Leaf N, %), leaf phosphorus (Leaf P, mg g^{-1}), leaf area (LA, cm^2), leaf dry matter content (LDMC, %), plant height (Height, cm), specific leaf area (SLA, cm^2 g^{-1}), stem dry matter content (SDMC, %), root nitrogen (Root N, %), root phosphorus (Root P, mg g^{-1}), root dry matter content (RDMC, %), and stomatal traits (density, length, width, and area). All traits were standardized (z-score; mean = 0, SD = 1) prior to PCA. Points represent sites and are colored by region; arrows indicate trait loadings. **PC1 captures the plant economic spectrum (higher values indicate more acquisitive strategies, with SLA loading positively), whereas PC2 reflects an orthogonal axis of trait variation primarily associated with plant size-related attributes**.

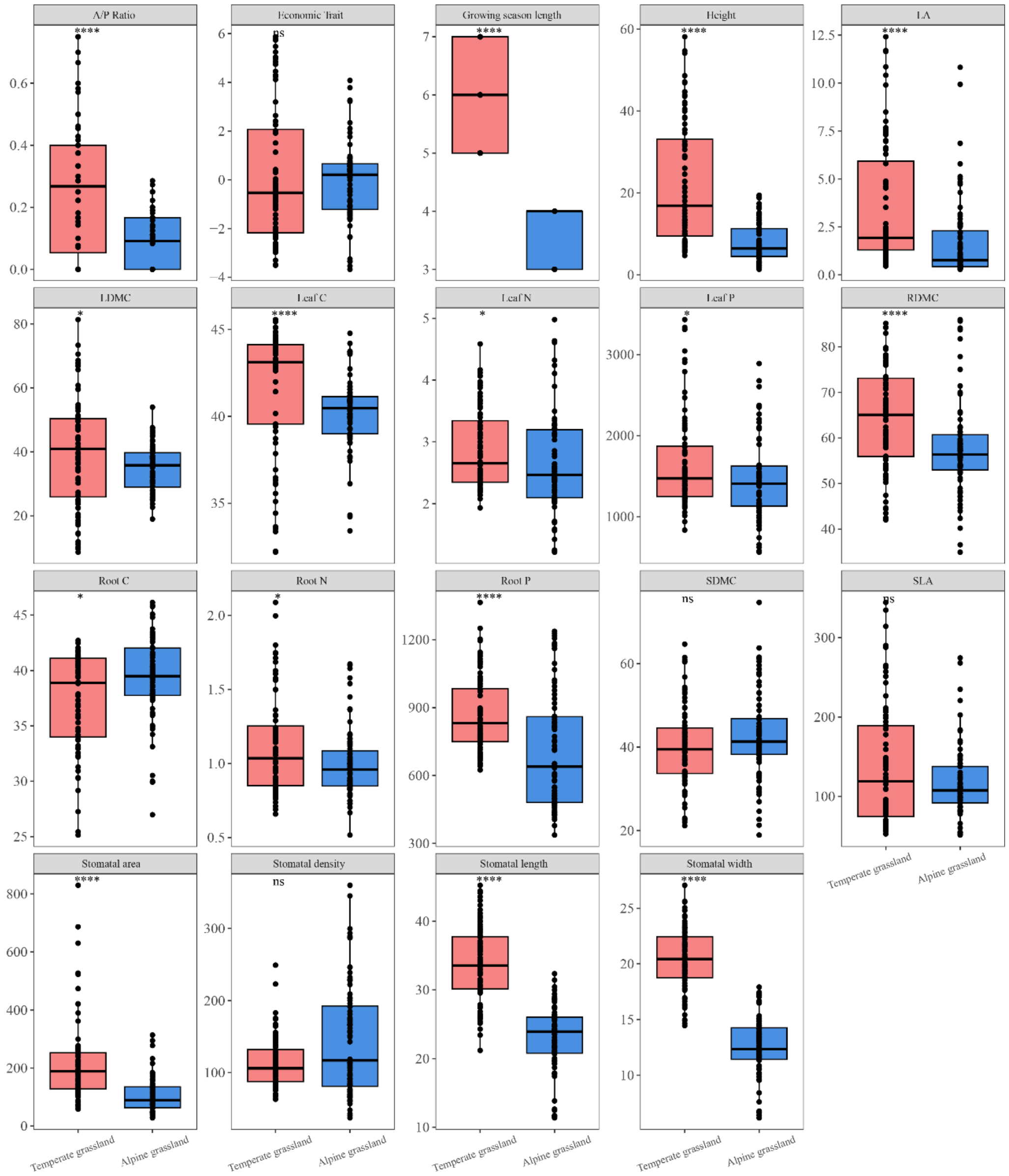

Figure S4. Comparison of species and functional composition between temperate and alpine grasslands. A/P ratio: the abundance ratio of annual and perennial plants; Leaf C: Leaf carbon concentration (%); Leaf N: Leaf nitrogen concentration (%); Leaf P: Leaf phosphorus concentration (mg g^{-1}); LA: Leaf area (cm^2); LDMC: Leaf dry mass content (%); Height: Plant height (cm); SLA: Specific leaf area (cm^2 g^{-1}); SDMC: Stem dry mass content (%); Root N: Root nitrogen concentration (%); Root P: Root phosphorus concentration (mg g^{-1}); RDMC: Root dry mass content (%). The statistical significance of differences was tested using the non-parametric Wilcoxon test for two independent samples. Significance levels are indicated as follows: ns (not significant): $p > 0.05$, $^{*}p \leq 0.05$, $^{**}p \leq 0.01$, $^{***}p \leq 0.001$. Group differences shown in this figure were assessed using frequentist tests, and detailed Bayesian comparisons, including posterior estimates and 95% credible intervals, are reported in Supplementary Table 2 and support the same conclusions.

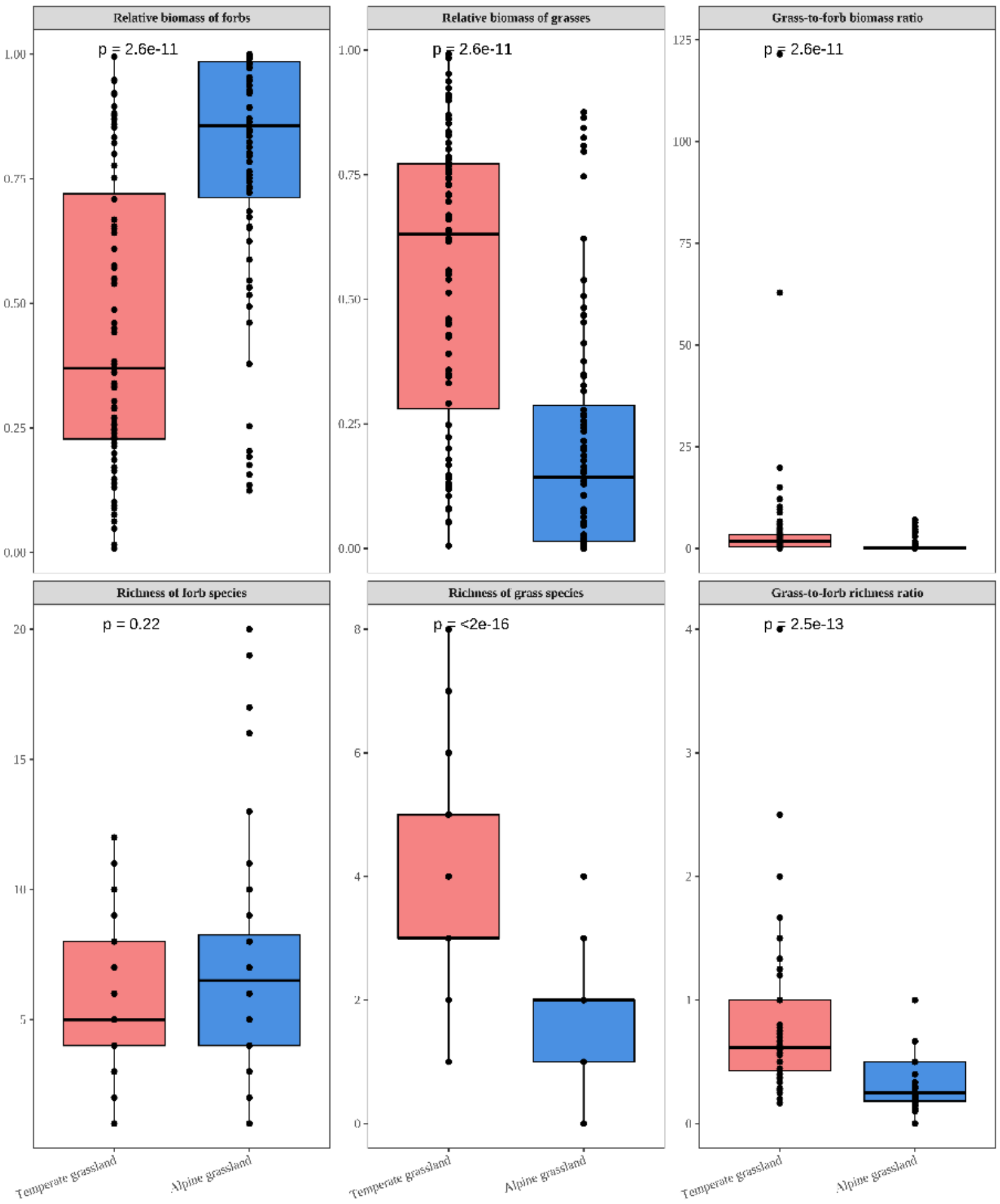

Figure S5. Grass and forb composition in temperate and alpine grasslands. The figure illustrates significant differences in the relative biomass and richness of grasses and forbs across the two grassland types.

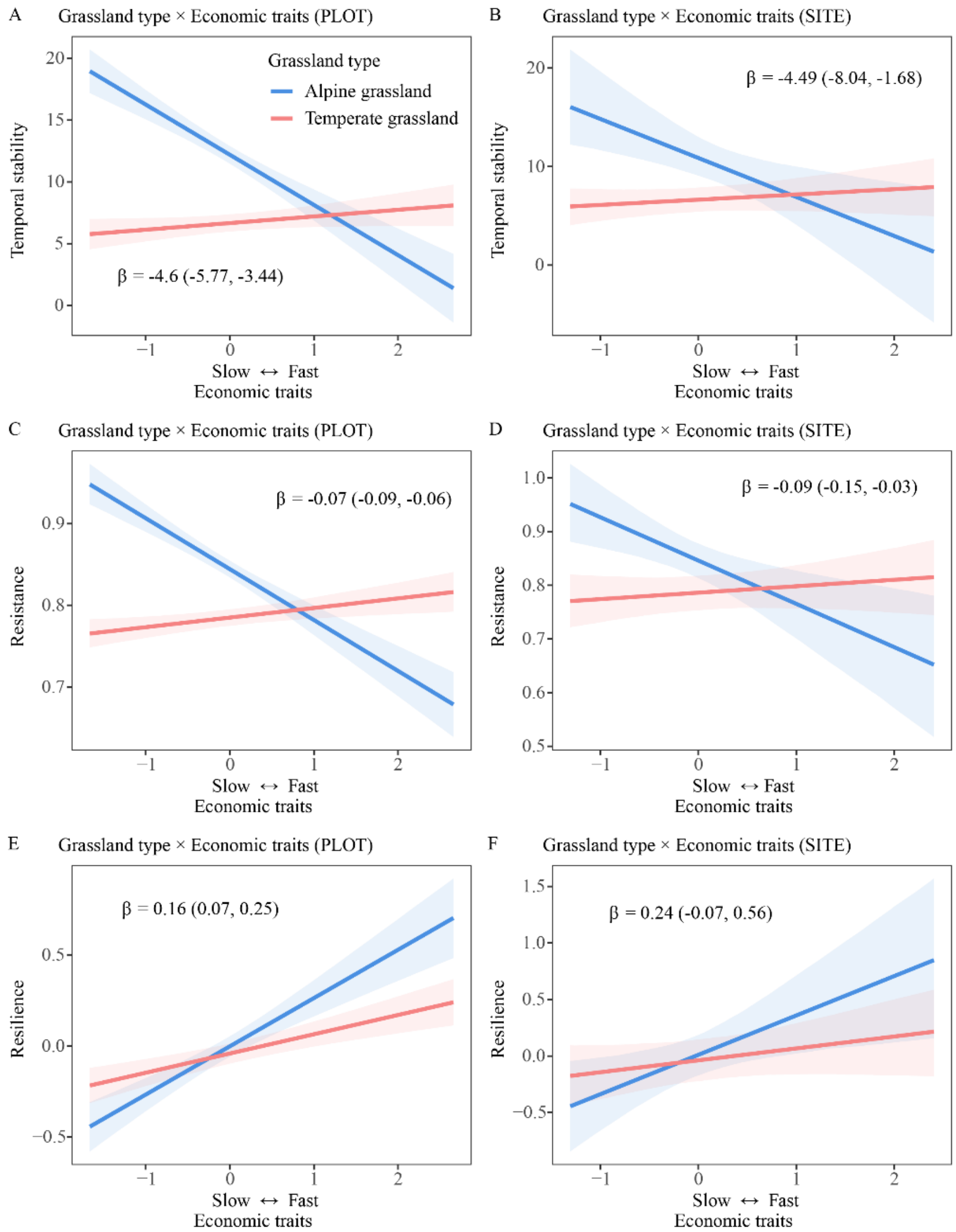

Figure S6. Main effects of economic traits, grassland type, and their interaction on components of ecosystem stability at plot and site levels. Plot-level analyses (A, C, E) are based on 160 plots. Site-level analyses (B, D, F) are based on 20 sites. (A, C, E) Posterior estimates of standardized effect sizes for economic traits, grassland type, and their interaction (grassland type × economic traits) on temporal stability (A), resistance (C), and resilience (E), from a multivariate Bayesian model fitted to plot-level data. (B, D, F) Posterior estimates of standardized effect sizes for the same predictors on temporal stability (B), resistance (D), and resilience (F), from the corresponding multivariate Bayesian model fitted to site-level data. Line colors indicate grassland type: red = temperate grassland, blue = alpine grassland. **Across both plot and site levels, trait–stability relationships are context dependent, with clear interaction effects for temporal stability and resistance, whereas the interaction for resilience is substantially weaker.**

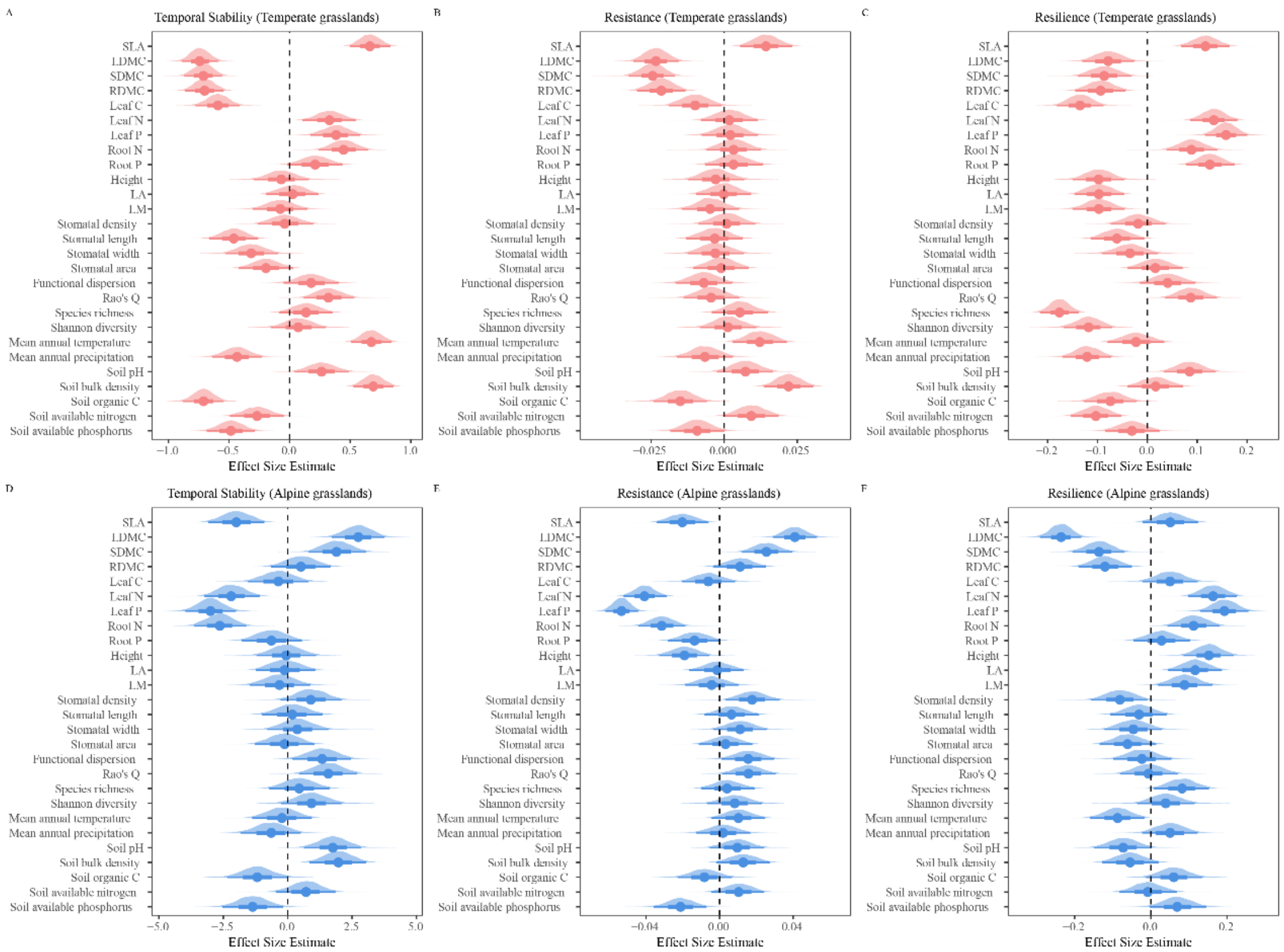

Figure S7. Effects of abiotic factors (mean annual temperature and mean annual precipitation) and biotic factors—community-weighted mean (CWM) traits, functional diversity (functional dispersion, Rao's Q), and taxonomic diversity (species richness, Shannon diversity)—on three facets of ecosystem stability: temporal stability, resistance, and resilience. Panels A–C show results for temperate grasslands, and panels D–F show results for alpine grasslands. Each panel presents posterior effect-size distributions from a multivariate Bayesian model with the three response variables. Circles indicate mean standardized effect sizes; thick and thin horizontal bars show the 80 % and 95 % credible intervals, respectively. Plant-trait CWMs include Leaf C (leaf carbon concentration, %), Leaf N (leaf nitrogen concentration, %), Leaf P (leaf phosphorus concentration, mg g^{-1}), LA (leaf area, cm^2), LDMC (leaf dry-matter content, g g^{-1}), Height (plant height, cm), SLA (specific leaf area, cm^2 g^{-1}), SDMC (stem dry-matter content, g g^{-1}), Root N (root nitrogen concentration, %), Root P (root phosphorus concentration, mg g^{-1}), and RDMC (root dry-matter content, %). All predictors were standardized (z-score, mean = 0, SD = 1) prior to analysis, producing directly comparable effect size estimates across variables.

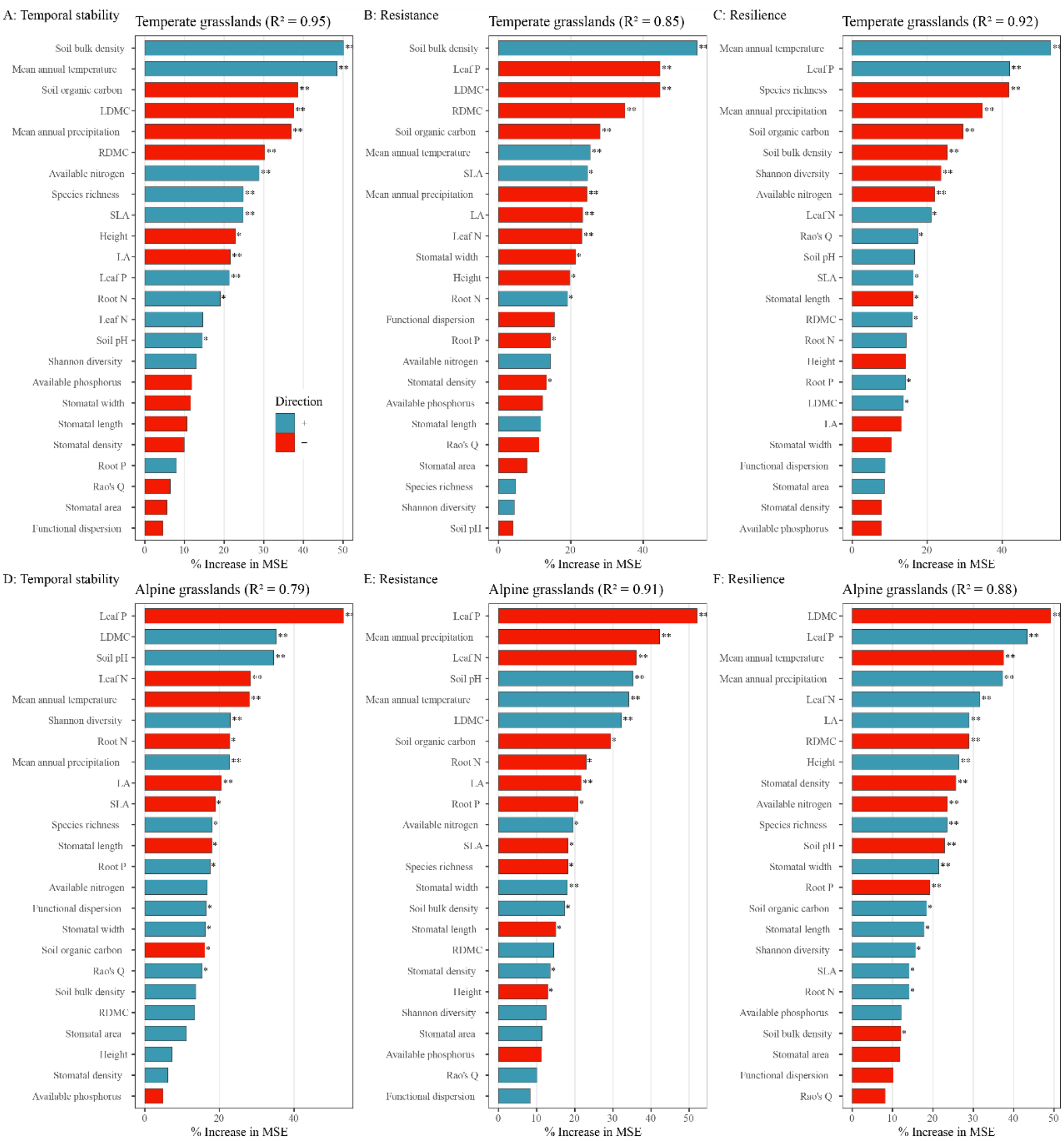

Figure S8. Results of random forest analyses showing the relative importance of predictor variables for explaining ecosystem temporal stability, resistance, and resilience. Panels A–C show results for temperate grasslands, and panels D–F show results for alpine grasslands. Predictors include abiotic factors (mean annual temperature and mean annual precipitation) and biotic factors—community-weighted mean (CWM) traits, functional diversity (functional dispersion, Rao's Q), and taxonomic diversity (species richness, Shannon diversity). CWM traits analyzed are: Leaf C (leaf carbon concentration, %), Leaf N (leaf nitrogen concentration, %), Leaf P (leaf phosphorus concentration, mg g^{-1}), LA (leaf area, cm^2), LDMC (leaf dry-matter content, g g^{-1}), Height (plant height, cm), SLA (specific leaf area, cm^2 g^{-1}), SDMC (stem dry-matter content, g g^{-1}), Root N (root nitrogen concentration, %), Root P (root phosphorus concentration, mg g^{-1}), and RDMC (root dry-matter content, %). Variable importance was quantified as the percentage increase in mean squared error (%IncMSE) when each predictor was permuted.

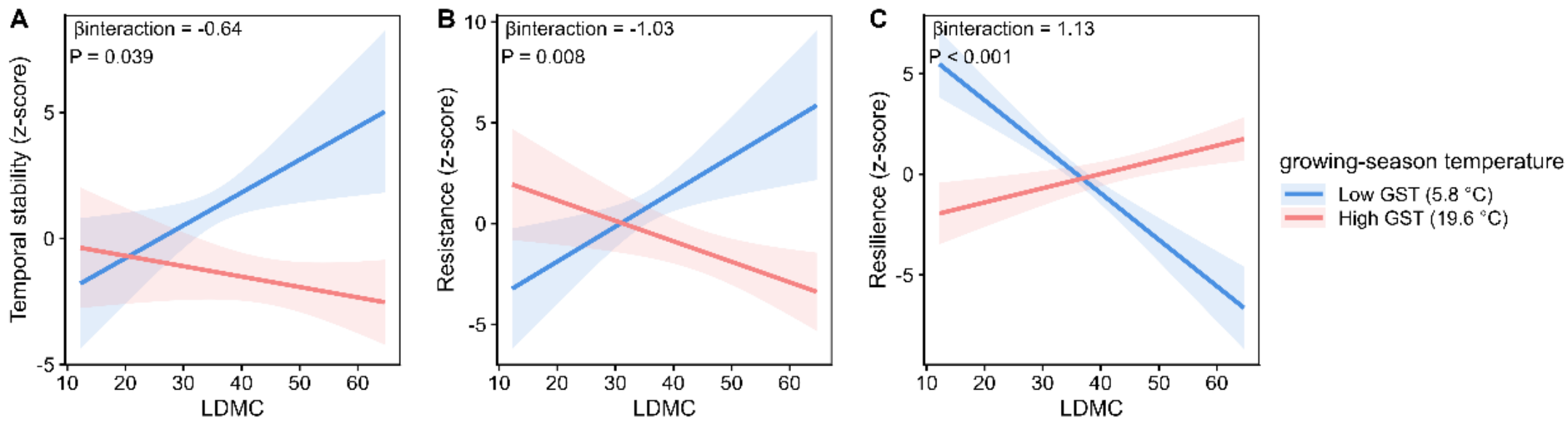

Figure S9. Bayesian estimates of the interactive effects of leaf dry matter content and growing-season temperature on ecosystem stability components. Model-predicted relationships between leaf dry matter content (LDMC, %) and standardized temporal stability (A), resistance (B), and resilience (C) are shown under low and high growing-season temperature (GST), corresponding to the temperature regimes of the alpine and temperate grasslands, respectively. This temperature-based contrast is consistent with, and complementary to, the grassland-type moderation shown in Figure S6. Lines represent posterior mean predictions, and shaded bands indicate 95% credible intervals. The annotation in each panel reports the posterior mean of the LDMC × GST interaction coefficient (β), with values below zero indicating that the LDMC effect becomes weaker or more negative at higher GST and values above zero indicating that the LDMC effect becomes stronger or more positive at higher GST. Values in brackets are 95% credible intervals. Pr(β > 0) or Pr(β < 0) denotes the posterior probability that the interaction coefficient is positive or negative, respectively; values ⩾ 0.95 were interpreted as strong directional support for the sign of the interaction effect. Models included Rao's quadratic entropy, species richness, and soil pH as covariates. Response variables were standardized before model fitting.

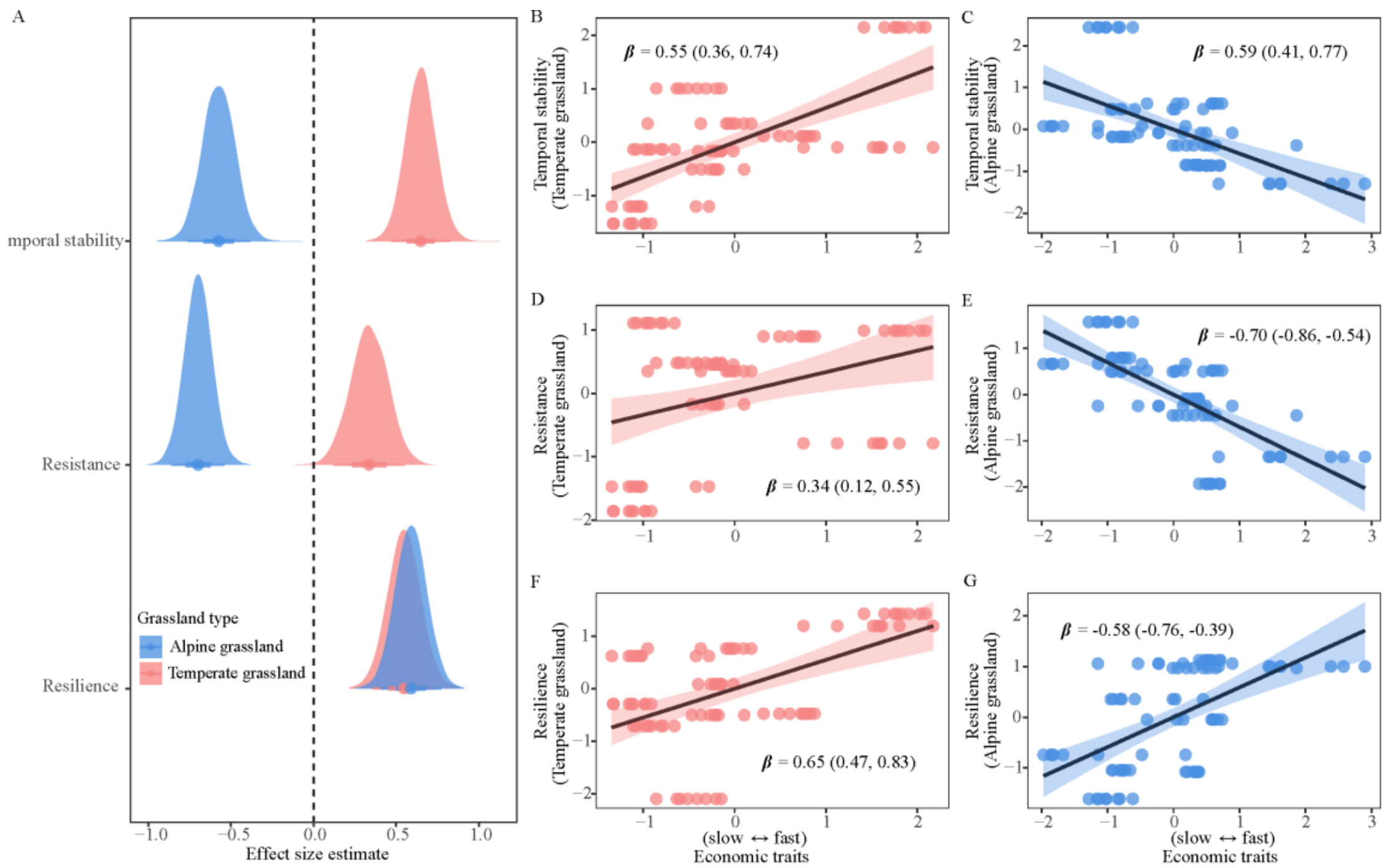

Figure S10. Effects of economic traits on ecosystem temporal stability, resistance, and resilience at the plot level. Panel A displays posterior distributions of effect sizes, with circles indicating mean standardized effect sizes alongside 80% (thick lines) and 95% (thin lines) credible intervals derived from multivariate Bayesian models. Panels B–G display the relationships between economic traits and temporal stability, resistance, and resilience. Each data point corresponds to a single plot. In Panels B–G, solid lines with shaded areas represent mean model predictions with their 95% credible intervals. Economic traits are represented by the first principal component from the principal component analysis of nine individual functional traits, where larger values indicate more acquisitive strategies and lower values indicate more conservative strategies.

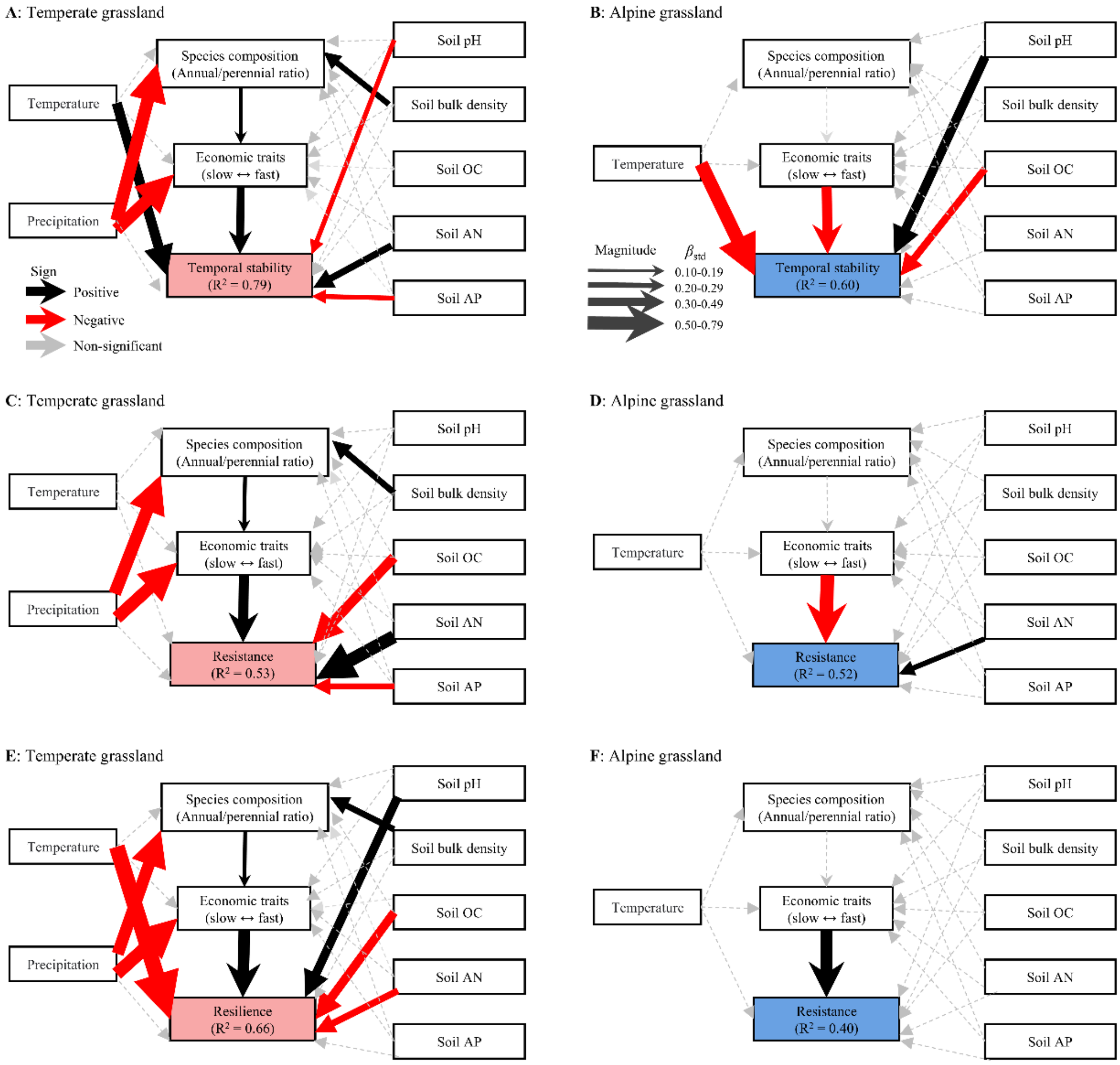

Figure S11. Results of plot-level Bayesian structural equation models showing the direct and indirect effects of climate (mean annual temperature and precipitation), soil properties (soil pH, bulk density, organic carbon, available nitrogen, and available phosphorus), species composition (annual vs. perennial plants), and economic traits on temporal stability (A and B), resistance (C and D), and resilience (E and F). Economic traits are represented by the first principal component from a principal component analysis of nine traits, where higher values reflect acquisitive strategies and lower values reflect conservative strategies. Arrow widths correspond to the magnitude of standardized path coefficients. Black and red arrows indicate positive and negative relationships, respectively; solid lines represent statistically significant effects, while dotted lines denote non-significant paths.

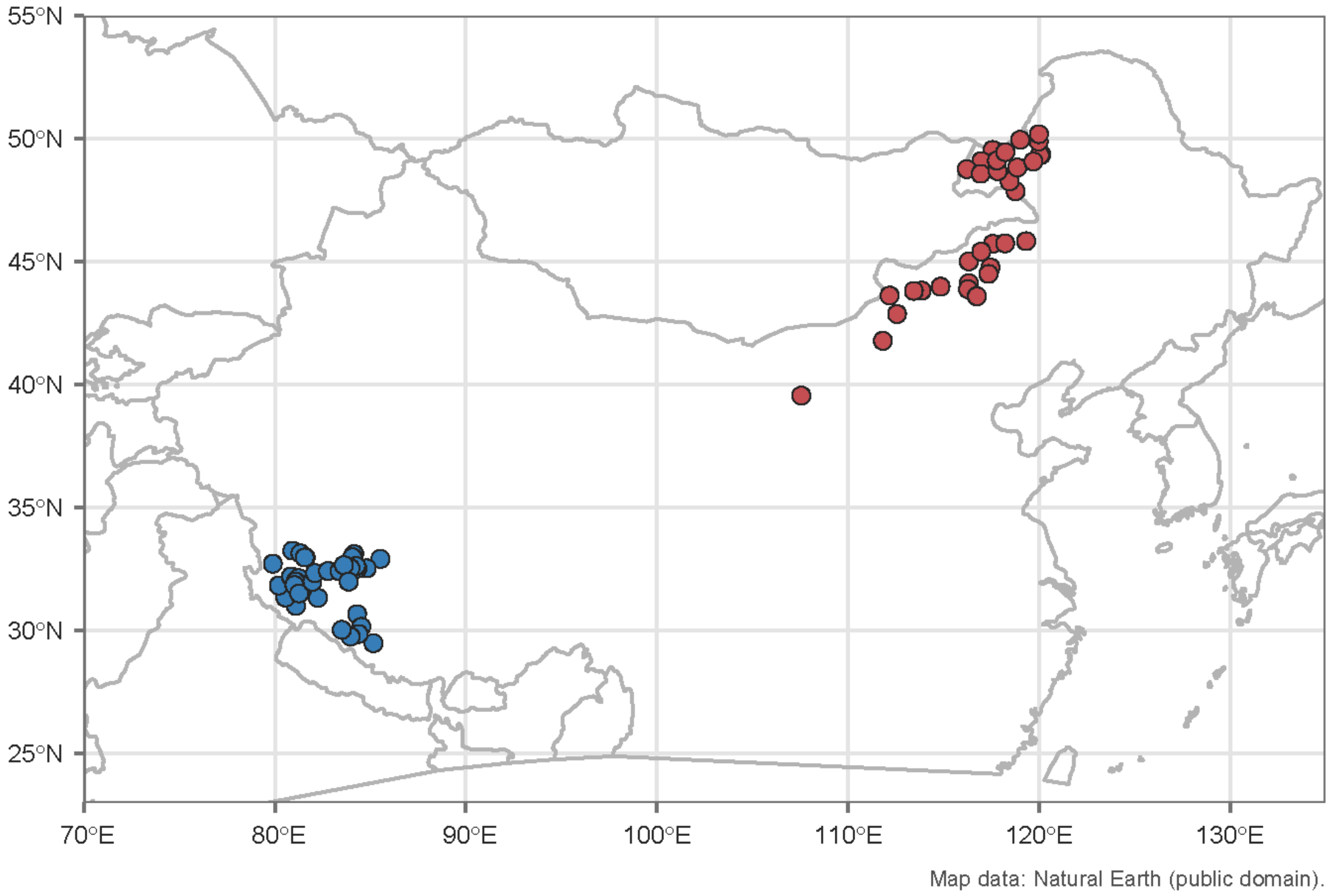

Figure S12. Geographic distribution of independent transect sites used for external validation. Blue circles indicate alpine grassland sites on the Tibetan Plateau (n = 33), and red circles indicate temperate grassland sites on the Mongolian Plateau (n = 35). The map was created by the authors in R using the sf and ggplot2 packages. Country boundaries and coastlines were obtained from Natural Earth (public domain; https://www.naturalearthdata.com/about/terms-of-use/) through the rnaturalearth package.

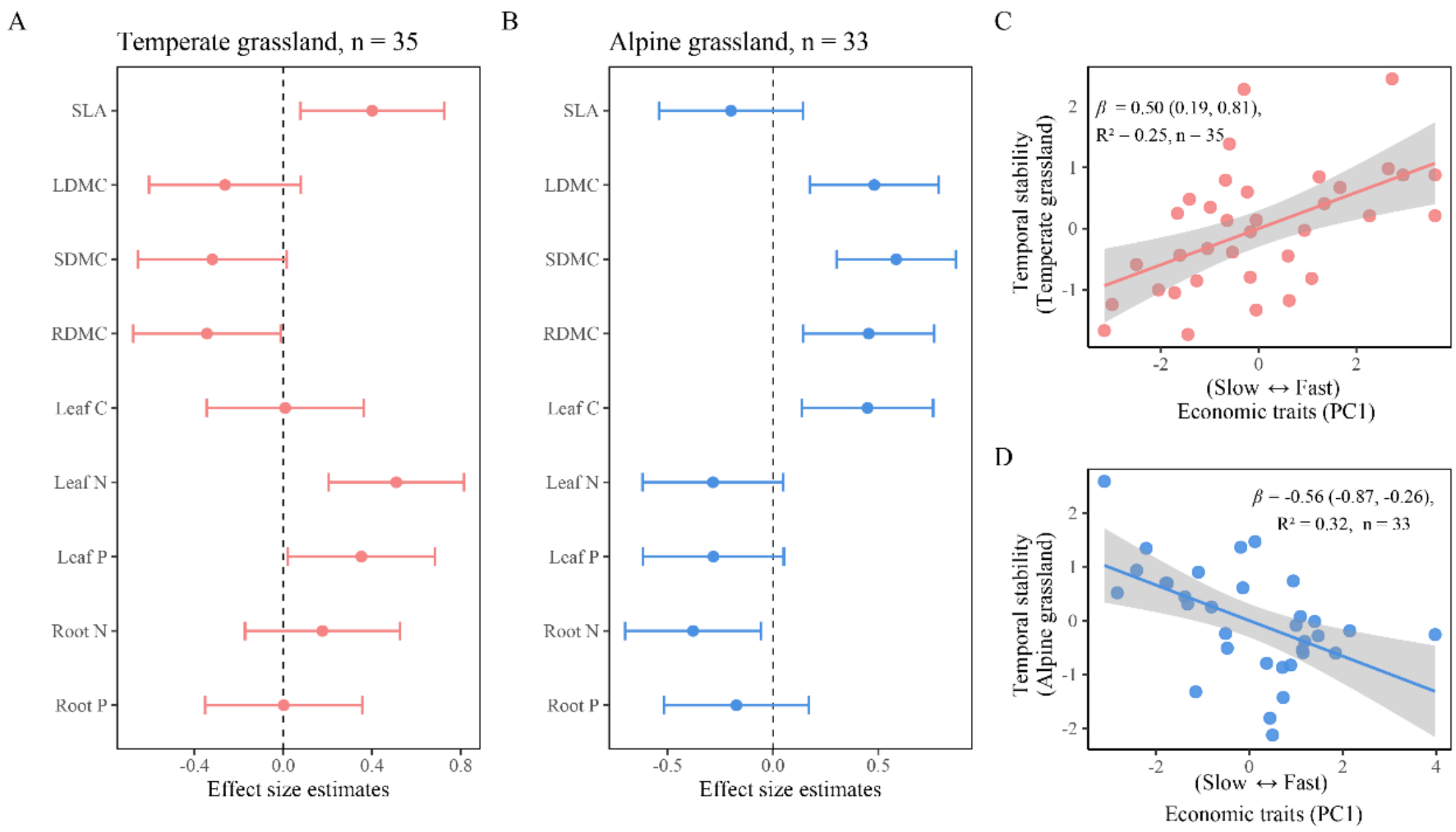

Figure S13. Independent-dataset validation: effects of plant economic traits on ecosystem temporal stability (temperate transect: n = 35; alpine transect: n = 33). Panels A–B display standardized effect sizes (posterior means ± 95% credible intervals) from biome-specific models. Panels C–D show the relationships between economic traits and temporal stability; solid lines indicate mean model predictions and shaded bands are 95% credible intervals. Economic traits are summarized as the first principal component (PC1) derived from a principal component analysis of the economic-trait subset identified in the main analysis.

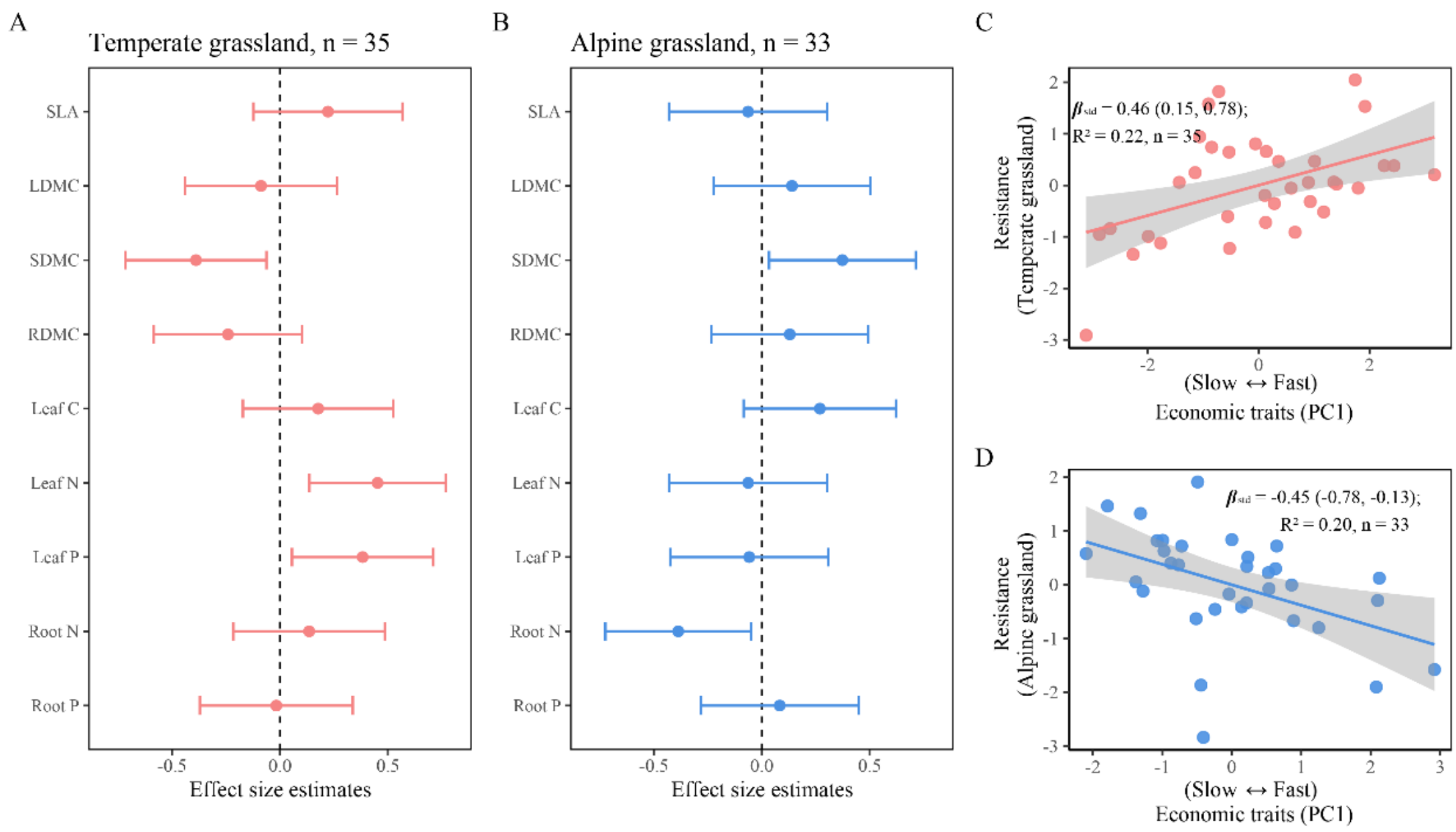

Figure S14. Independent-dataset validation: effects of plant economic traits on ecosystem resistance (temperate transect: n = 35; alpine transect: n = 33). Panels A–B display standardized effect sizes (posterior means ± 95% credible intervals) from biome-specific models. Panels C–D show the relationships between economic traits and resistance; solid lines indicate mean model predictions and shaded bands are 95% credible intervals. Economic traits are summarized as the first principal component (PC1) derived from a principal component analysis of the economic-trait subset identified in the main analysis.

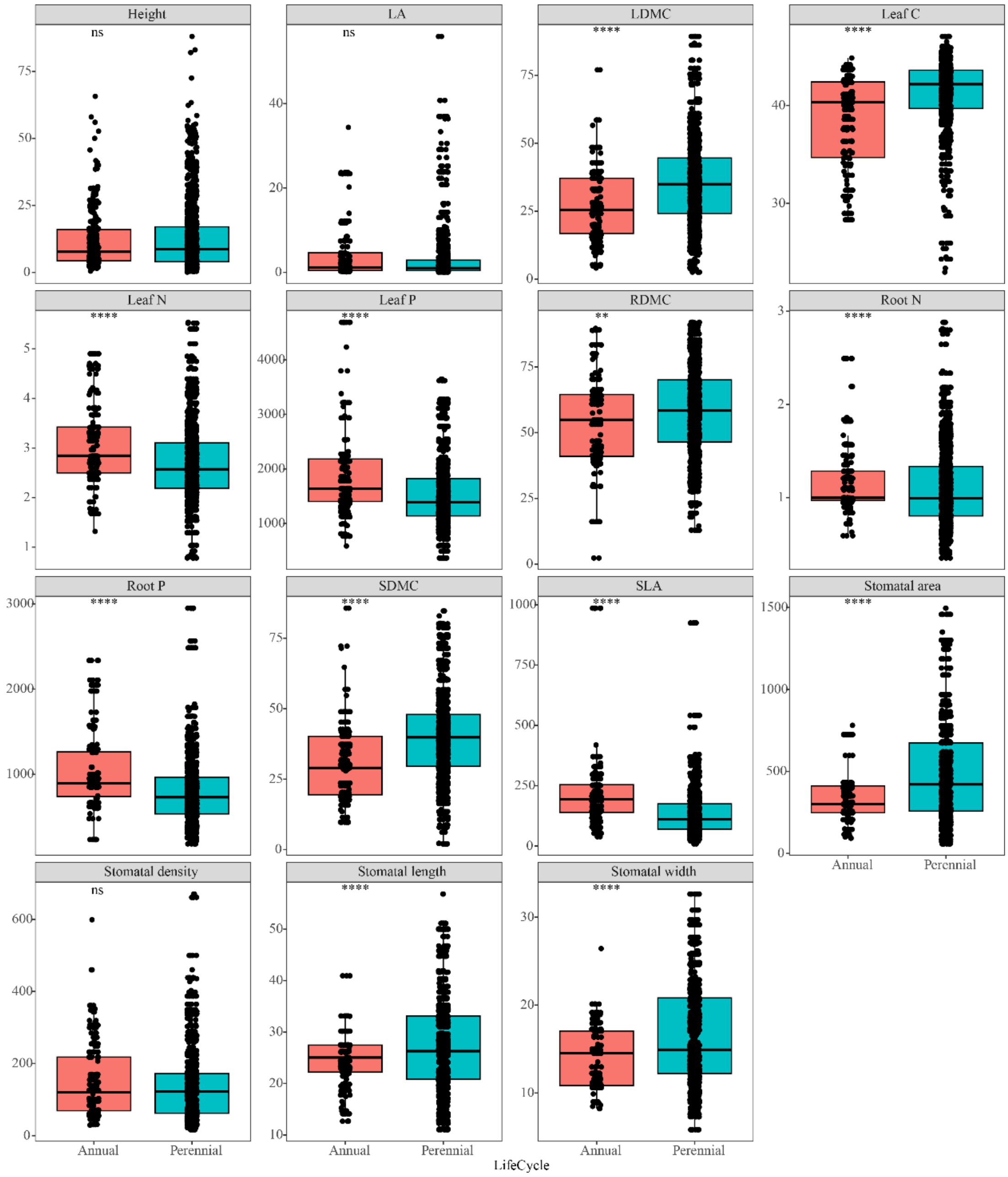

Figure S15. Comparison of mean trait values between annual and perennial plants. Boxplots show the interquartile range (IQR) within the box, with whiskers extending to either the minimum value or 1.5 times the IQR, whichever is more extreme, and the median indicated by a horizontal line. P values reflect the significant levels in the nonparametric Wilcoxon Rank Sum Test. Significance levels are indicated as follows: ns (not significant): p > 0.05, $^{*}p \leq 0.05$, $^{**}p \leq 0.01$, $^{***}p \leq 0.001$.

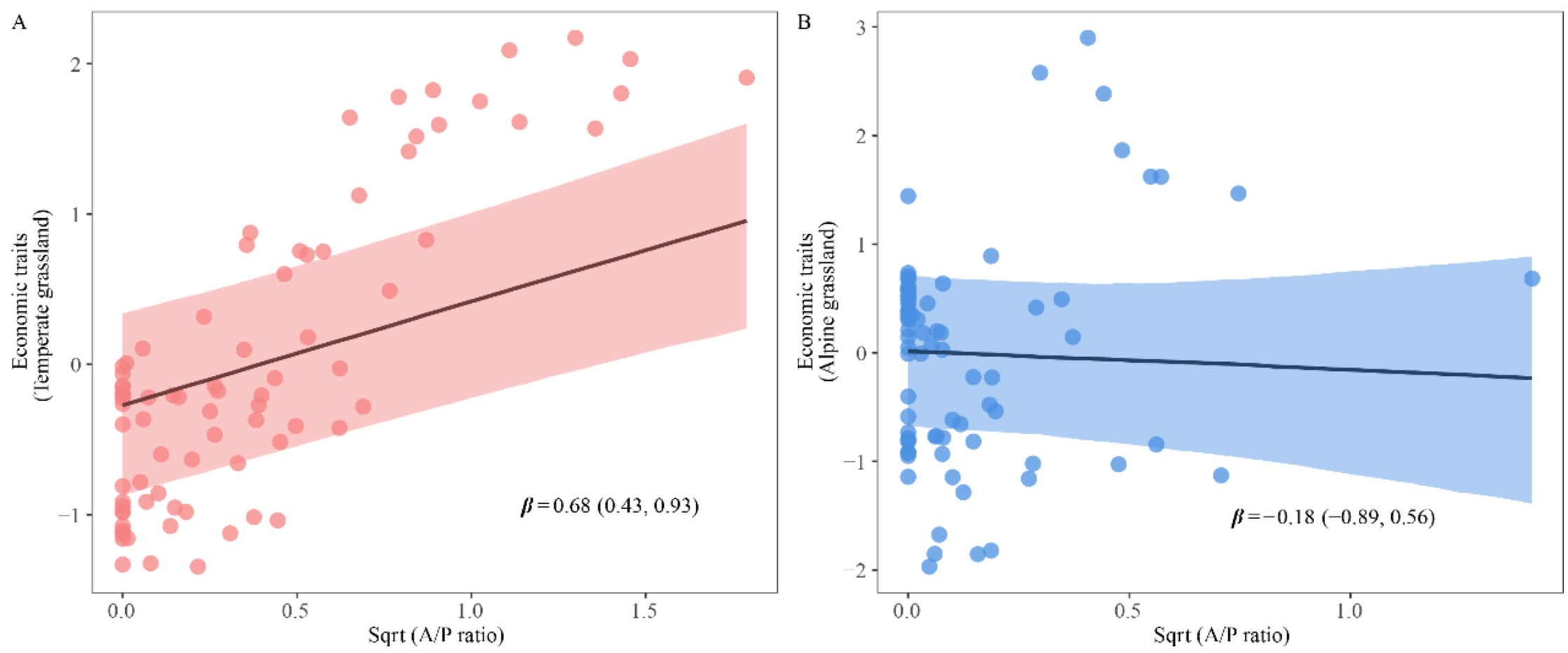

Figure S16. Relationships between community life-history composition and economic traits. Panels A and B show results for temperate and alpine grasslands, respectively. Community life-history composition is represented by the ratio of annual to perennial plants, whereas functional composition is characterized by plant economic traits.

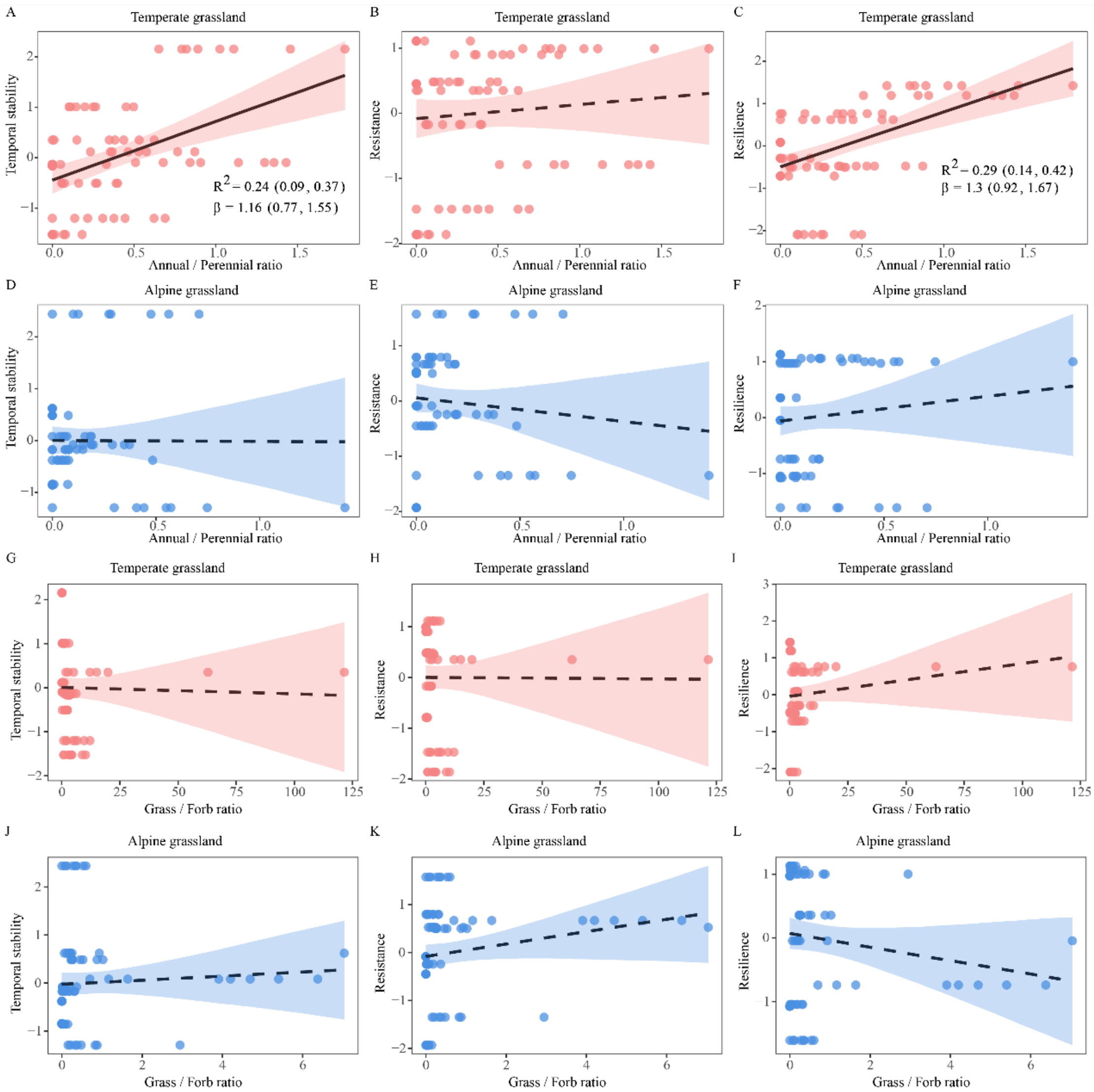

Figure S17. Relationships between species composition metrics and ecosystem stability dimensions. (A–C) Annual/perennial ratio versus temporal stability, resistance, and resilience in temperate grasslands. (D–F) Annual/perennial ratio versus the same stability components in alpine grasslands. (G–I) Grass/forb ratio versus temporal stability, resistance, and resilience in temperate grasslands. (J–L) Grass/forb ratio versus the same stability components in alpine grasslands. Dashed lines denote non-significant slopes (95 % credible intervals overlap zero).

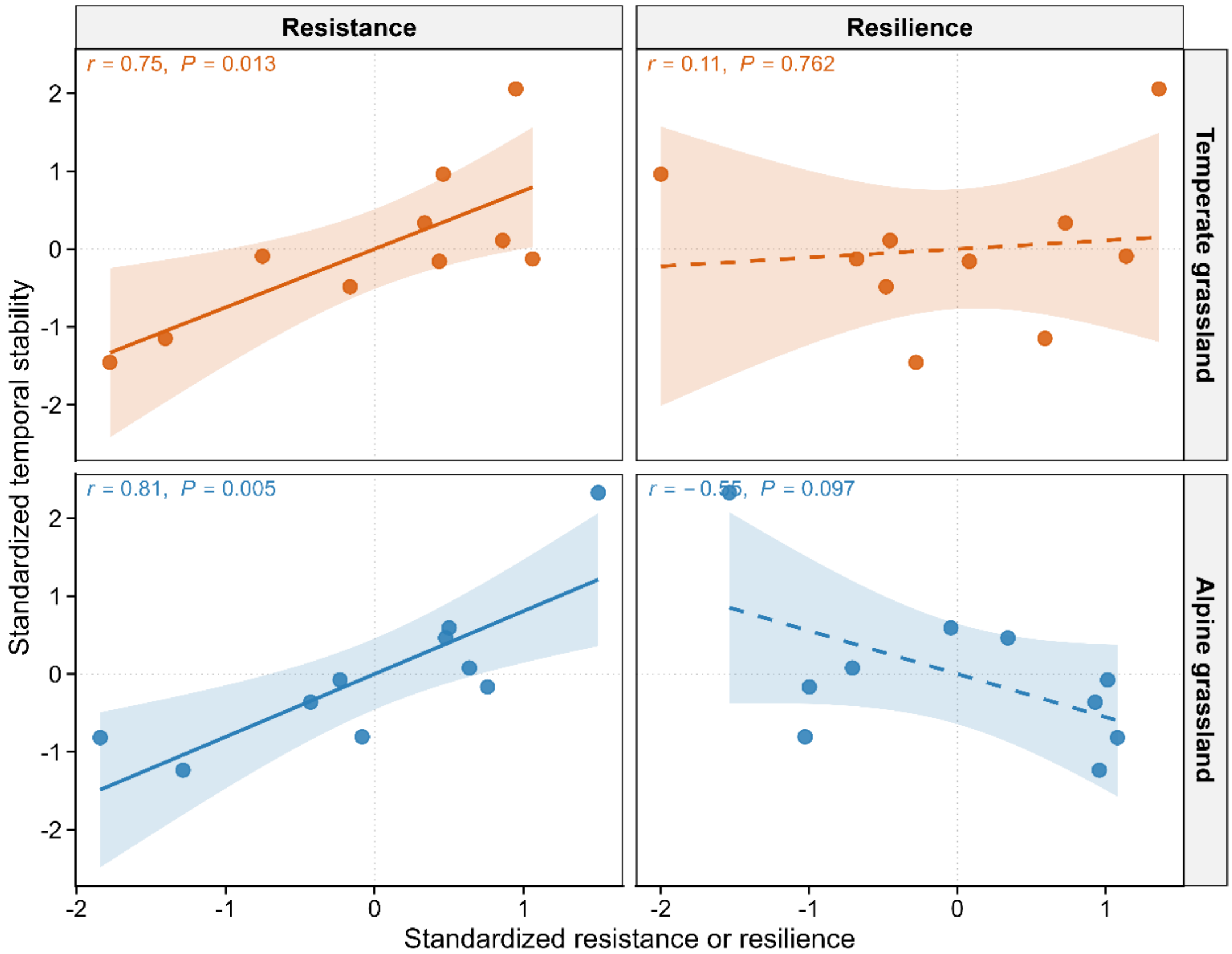

Figure S18. Site-level relationships between temporal stability and resistance or resilience across two grassland regions. Relationships between standardized temporal stability and standardized resistance or resilience were analyzed at the site level in temperate and alpine grasslands. Each point represents one site, with plot-level values first averaged within each site to avoid non-independence among plots from the same site. Temporal stability was quantified using the inverse coefficient of variation of EVI, while resistance and resilience were quantified using bounded stability metrics derived from EVI responses to climate extremes. All variables were standardized within each region before analysis to allow direct comparison of effect sizes. Solid and dashed fitted lines indicate significant and non-significant effects, respectively, based on region-specific multiple regression models including both resistance and resilience as predictors of temporal stability. Shaded bands represent 95% confidence intervals.

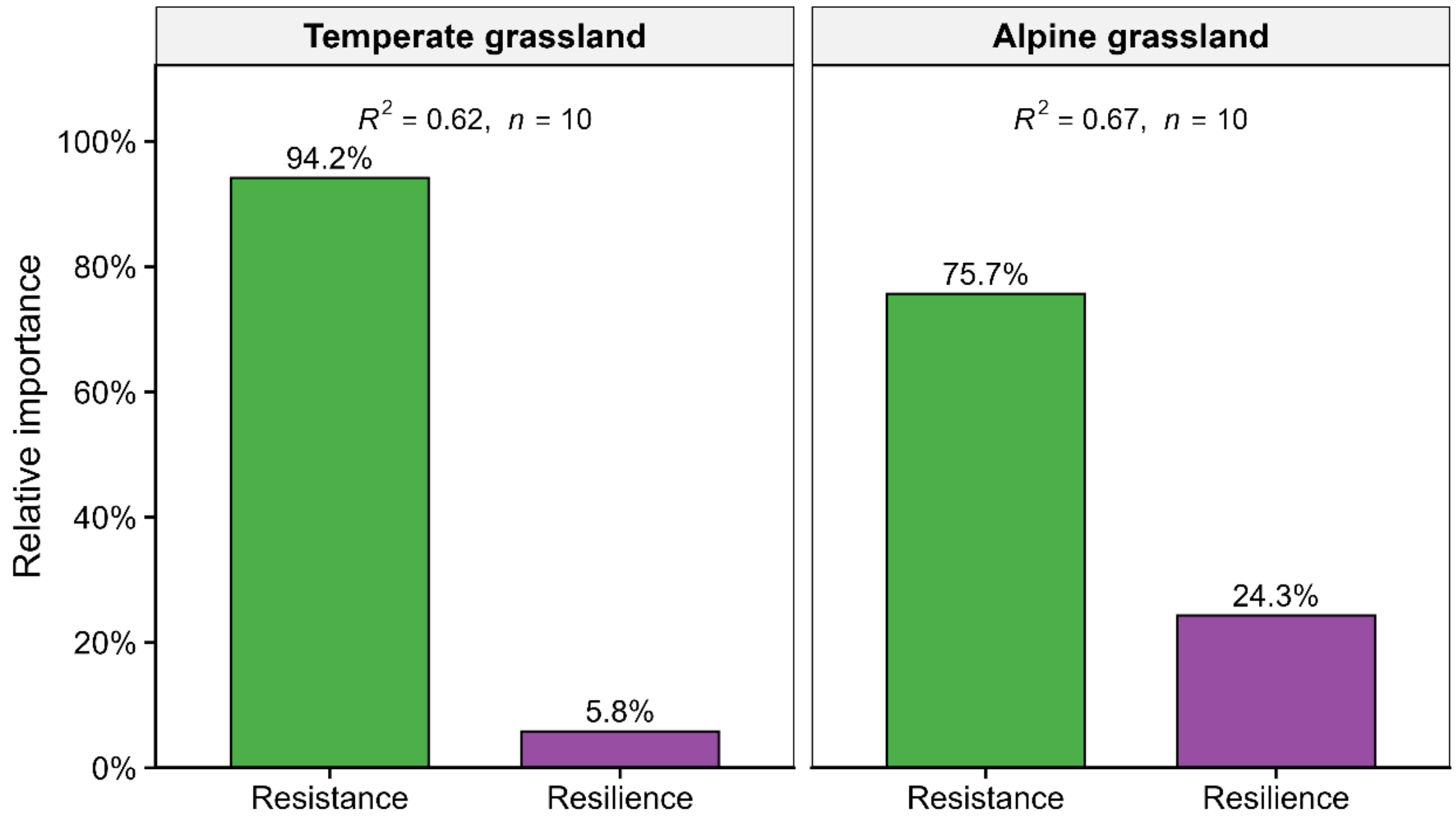

Figure S19. Relative contributions of resistance and resilience to temporal stability in temperate and alpine grasslands. Relative importance of resistance and resilience in explaining site-level temporal stability was quantified separately for temperate and alpine grasslands using region-specific multiple regression models. Temporal stability, resistance, and resilience were first averaged at the site level and then standardized within each region. Bars show the percentage contribution of each stability component to the explained variance in temporal stability, estimated using Lindeman–Merenda–Gold relative importance decomposition. Model R^2 and the number of sites are shown for each region. This analysis was used to evaluate whether resistance or resilience made greater contribution to ecosystem temporal stability in each grassland region.

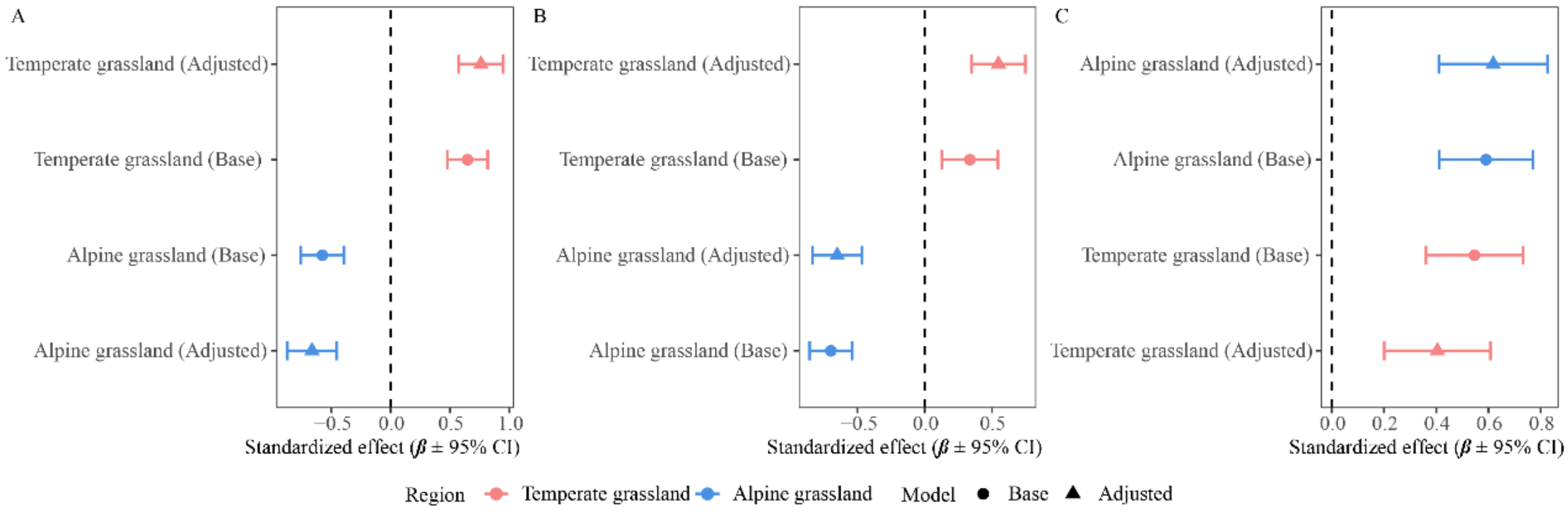

Figure S20. Economic-trait effects on ecosystem stability are robust to controlling for latitude and productivity across temperate and alpine grasslands. Panels A–C show standardized regression coefficients (***β***) and 95% confidence intervals for the effects of economic traits on temporal stability (A), resistance (B), and resilience (C). Points indicate model estimates (***β***) and horizontal bars denote 95% CIs; the dashed vertical line marks no effect (***β*** = 0). Colors denote grassland type (red = temperate, blue = alpine). Point shapes distinguish model specifications: Base models include economic traits only, whereas Adjusted models additionally include latitude and aboveground biomass (as a productivity proxy). Higher values of economic traits indicate a more acquisitive strategy.

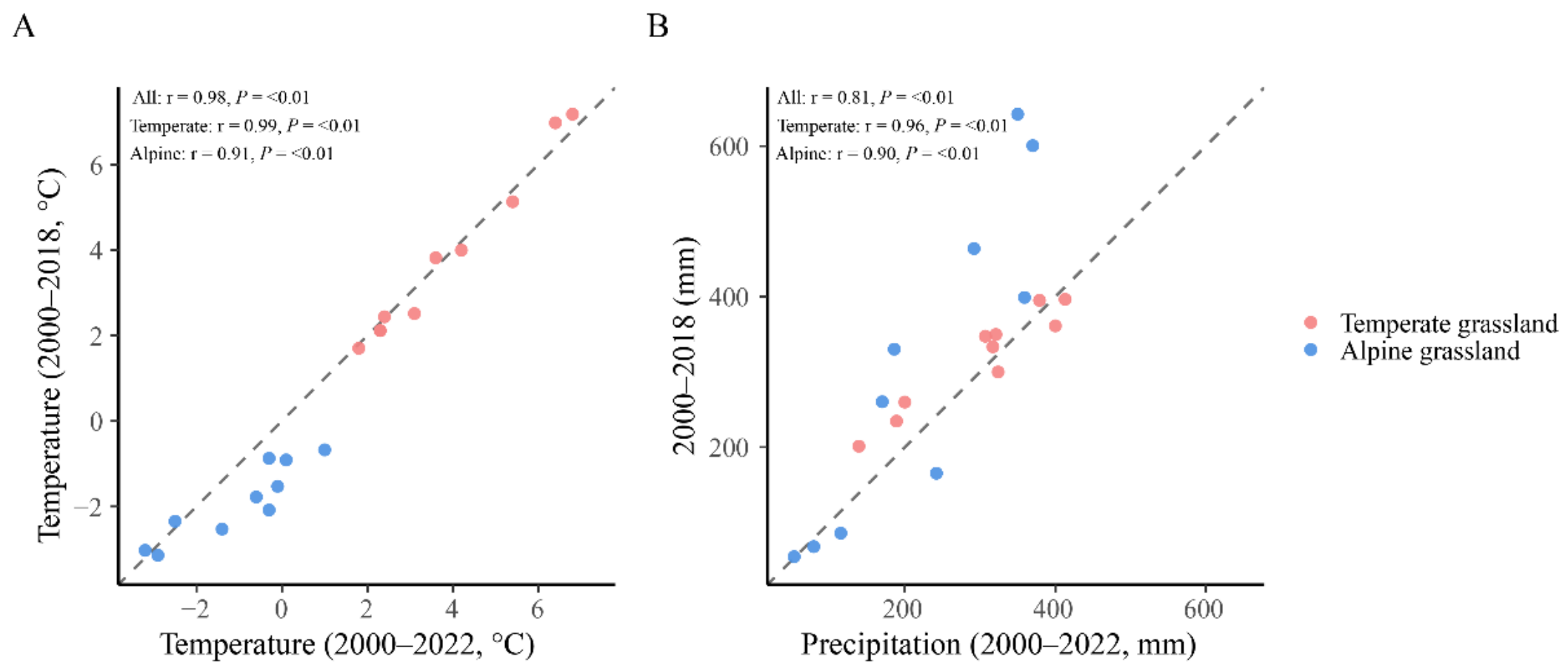

Figure S21. Comparison of site-level climate means between the full record (2000–2022) and the 2000–2018 window. Panels show (A) mean annual temperature and (B) mean annual precipitation. Each point represents a site, coloured by region (temperate grassland vs. alpine grassland). The dashed 1:1 line indicates perfect agreement between the two periods. Text in the upper-left of each panel reports Pearson correlations (r) and corresponding P values for all sites combined and for each region separately.

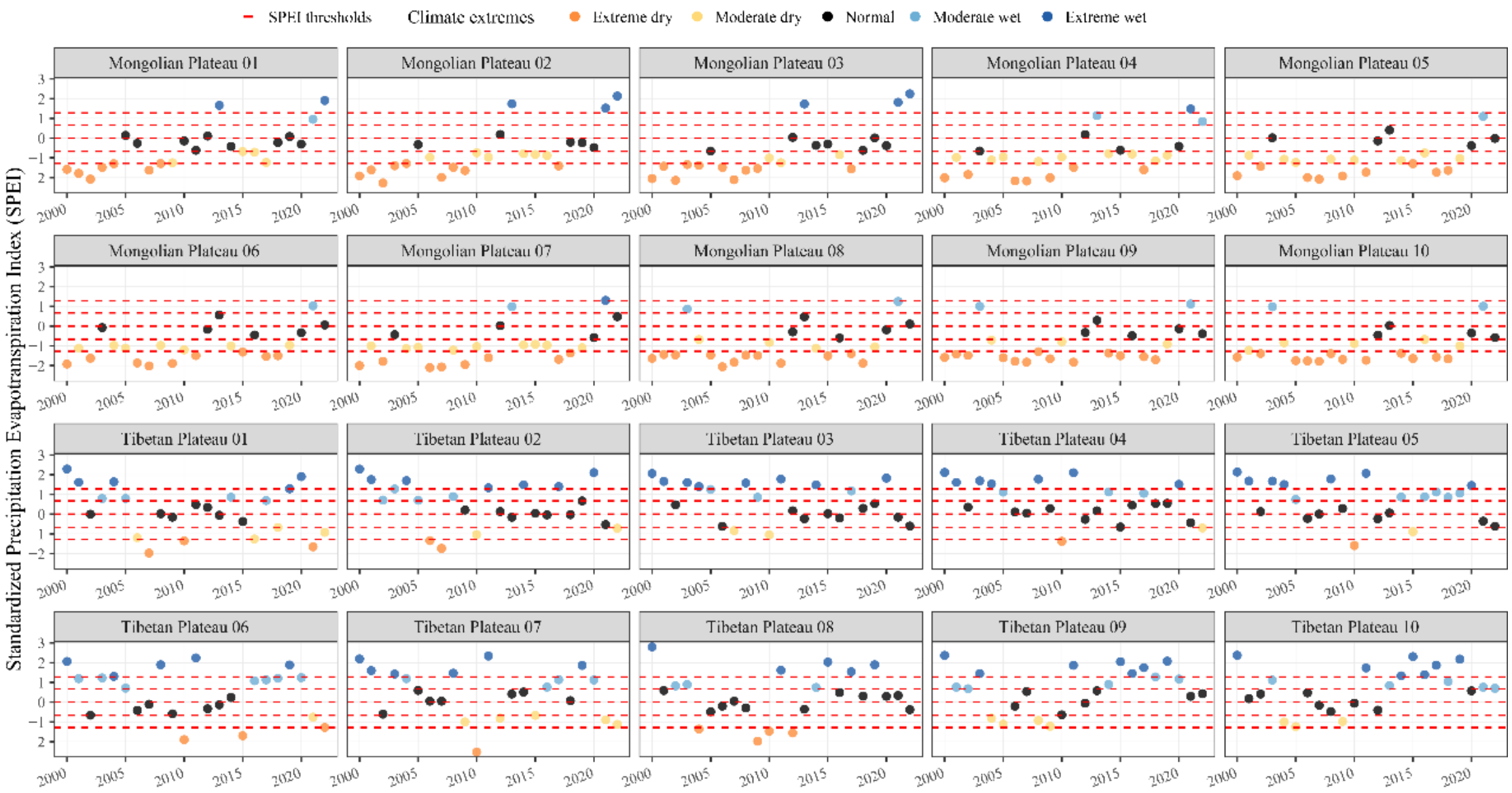

Figure S22. Temporal dynamics of climate extremes in temperate and alpine grasslands. Points show annual Standardized Precipitation Evapotranspiration Index (SPEI; yearly mean) for each site (facets) across the temperate grassland (Mongolian Plateau) and alpine grassland (Tibetan Plateau) regions. Point colours indicate the climate categories (e.g., extreme dry, moderate dry, normal, moderate wet, extreme wet) defined in this study (legend). Red dashed horizontal lines mark the SPEI thresholds used to classify climate extremes (−1.28, −0.67, 0, 0.67, 1.28); values below/above these cut-offs represent progressively drier/wetter conditions.

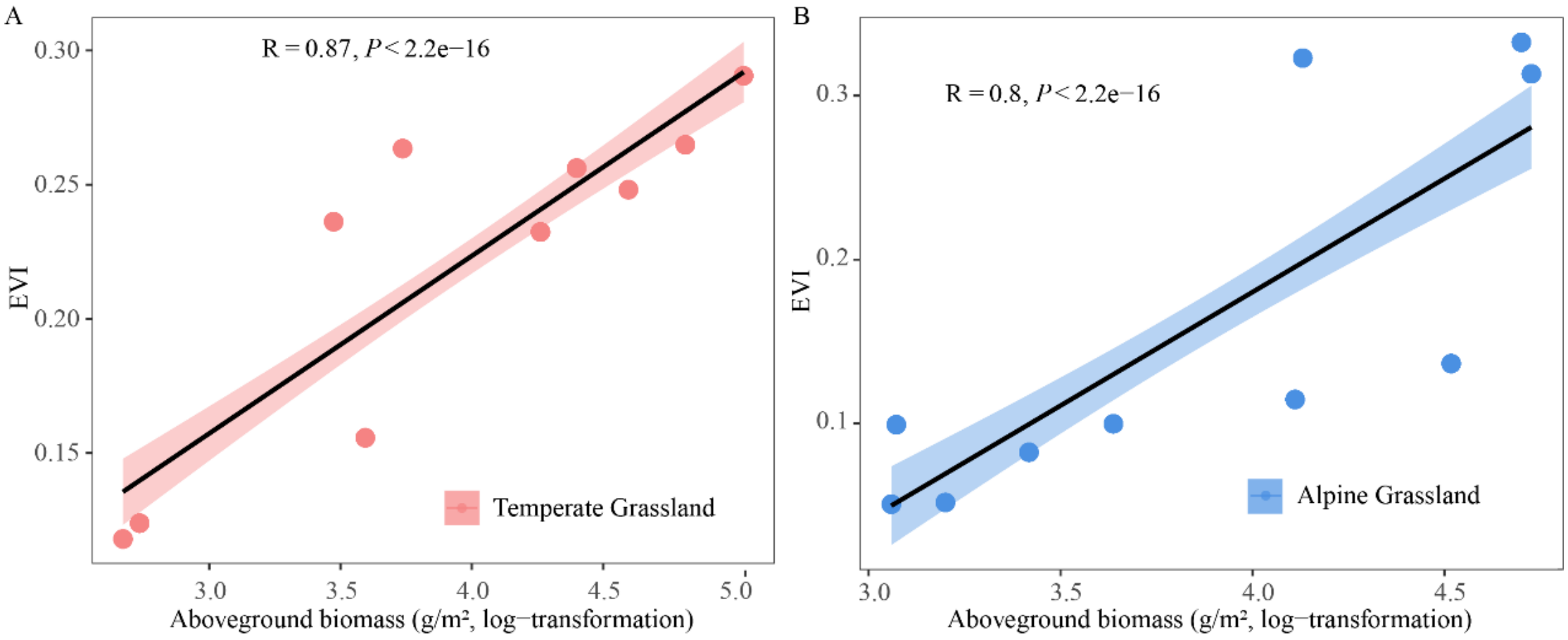

Figure S23. Relationships between field-measured aboveground biomass and remotely sensed EVI. Panels A and B show results for temperate grasslands (n = 10) and alpine grasslands (n = 10), respectively. Field-measured aboveground biomass (AGB, g m^{-2}) was strongly positively associated with the remotely sensed enhanced vegetation index (EVI) in both grassland types.

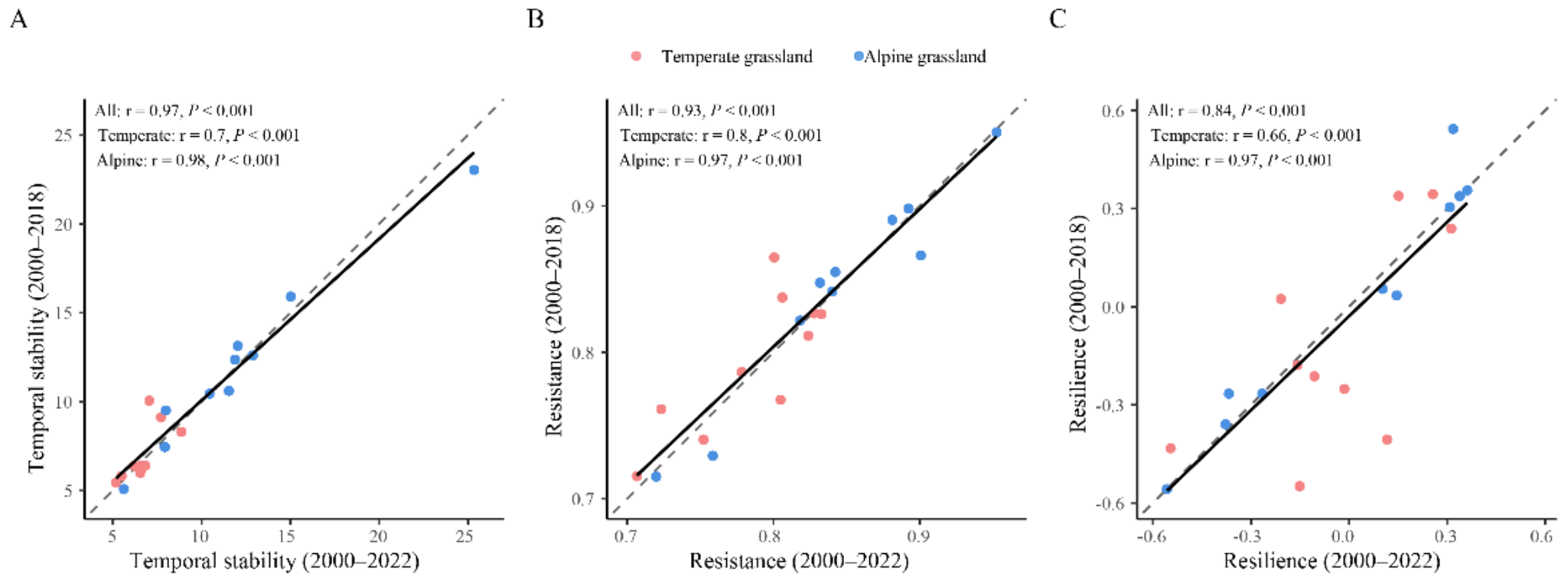

Figure S24. Consistency of EVI-based stability metrics between the full record (2000–2022) and the 2000–2018 subset. Panels show site-level comparisons of (A) temporal stability, (B) resistance, and (C) resilience calculated from MODIS EVI. The x-axis represents values computed using the full time series (2000–2022), and the y-axis represents values computed using the 2000–2018 subset. Points are colored by grassland type (temperate grassland, red; alpine grassland, blue). The dashed line indicates the 1:1 relationship, and the solid black line shows the fitted linear trend across all observations. Text in the upper-left reports correlation coefficients and ***P*** values for all sites combined and for each grassland type separately.

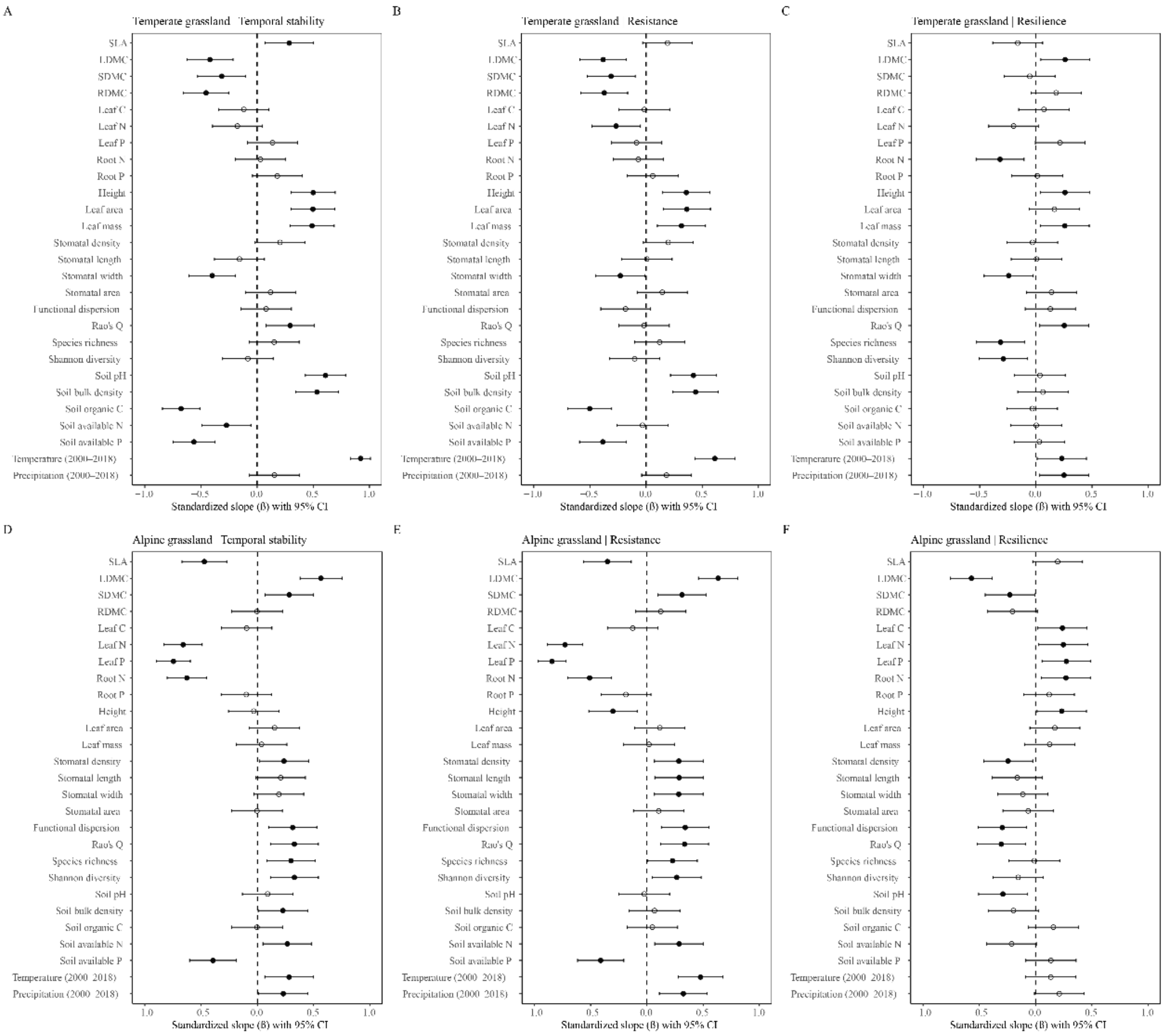

Figure S25. Robustness analysis of predictor–stability relationships using the corrected 2000–2018 observation window. Panels A–C show results for temperate grasslands on the Mongolian Plateau, and panels D–F show results for alpine grasslands on the Tibetan Plateau. Results are presented for temporal stability (A, D), resistance (B, E) and resilience (C, F). Predictors include abiotic factors (mean annual temperature and mean annual precipitation) and biotic factors: community-weighted mean (CWM) traits, functional diversity (functional dispersion and Rao's Q), and taxonomic diversity (species richness and Shannon diversity). Each panel summarizes standardized effect sizes estimated using separate single-predictor ordinary least-squares linear models. Circles indicate standardized slope estimates (β), and horizontal bars indicate 95% confidence intervals. All predictors and stability responses were standardized to z-scores (mean = 0, SD = 1), with response preprocessing described in the Methods. All three stability facets were calculated from the corrected 2000–2018 time series to assess the sensitivity of trait–stability relationships to the observation window.

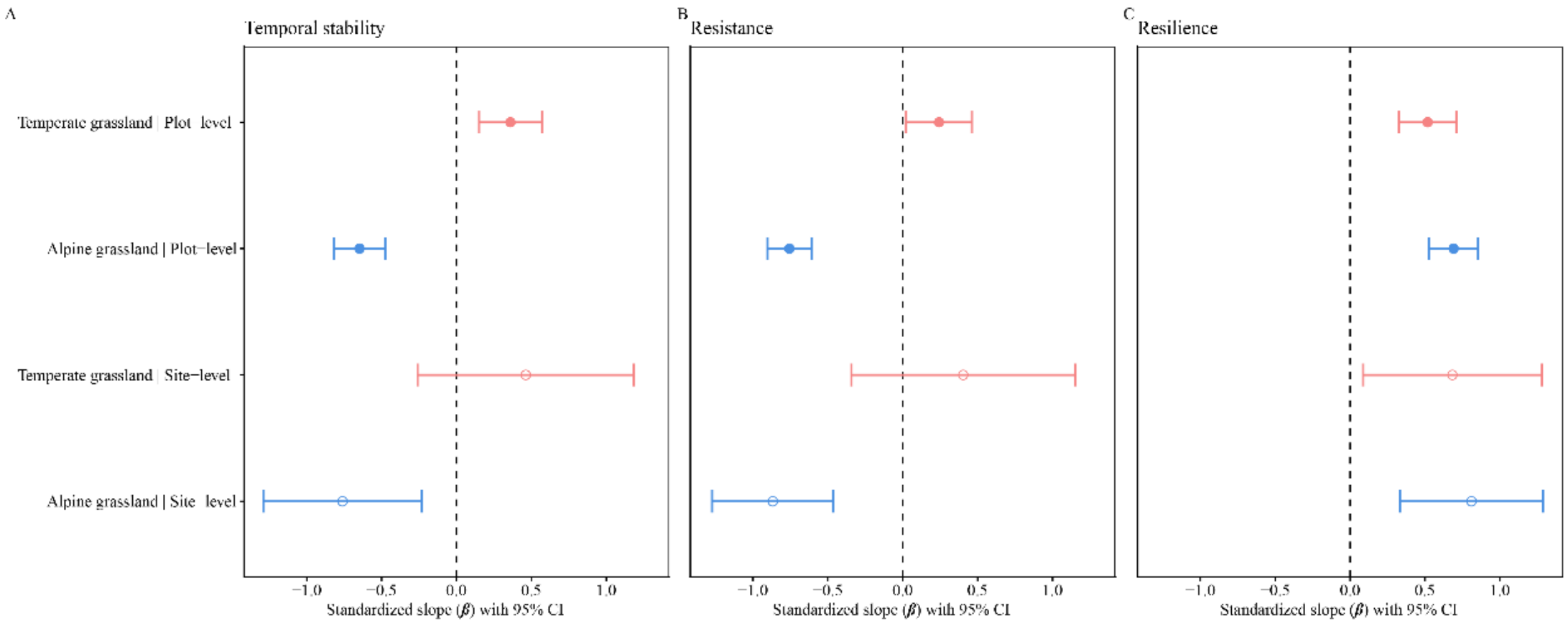

Figure S26. Robustness of economic strategy–stability relationships using the corrected 2000–2018 observation window. Panels A, B and C show relationships between the economic-strategy axis and temporal stability (TS), resistance (Rs) and resilience (Rl), respectively, in temperate and alpine grasslands. Forest plots summarize standardized regression slope estimates (*β*) and their 95% confidence intervals. All three stability components were calculated from MODIS EVI time series for the corrected 2000–2018 observation window. Positive *β* indicates that higher values on the economic-strategy axis are associated with higher values of the corresponding stability component, whereas negative *β* indicates the opposite. Colors distinguish temperate and alpine grasslands, and symbols distinguish plot-level models from site-level reanalyses using site-aggregated responses.

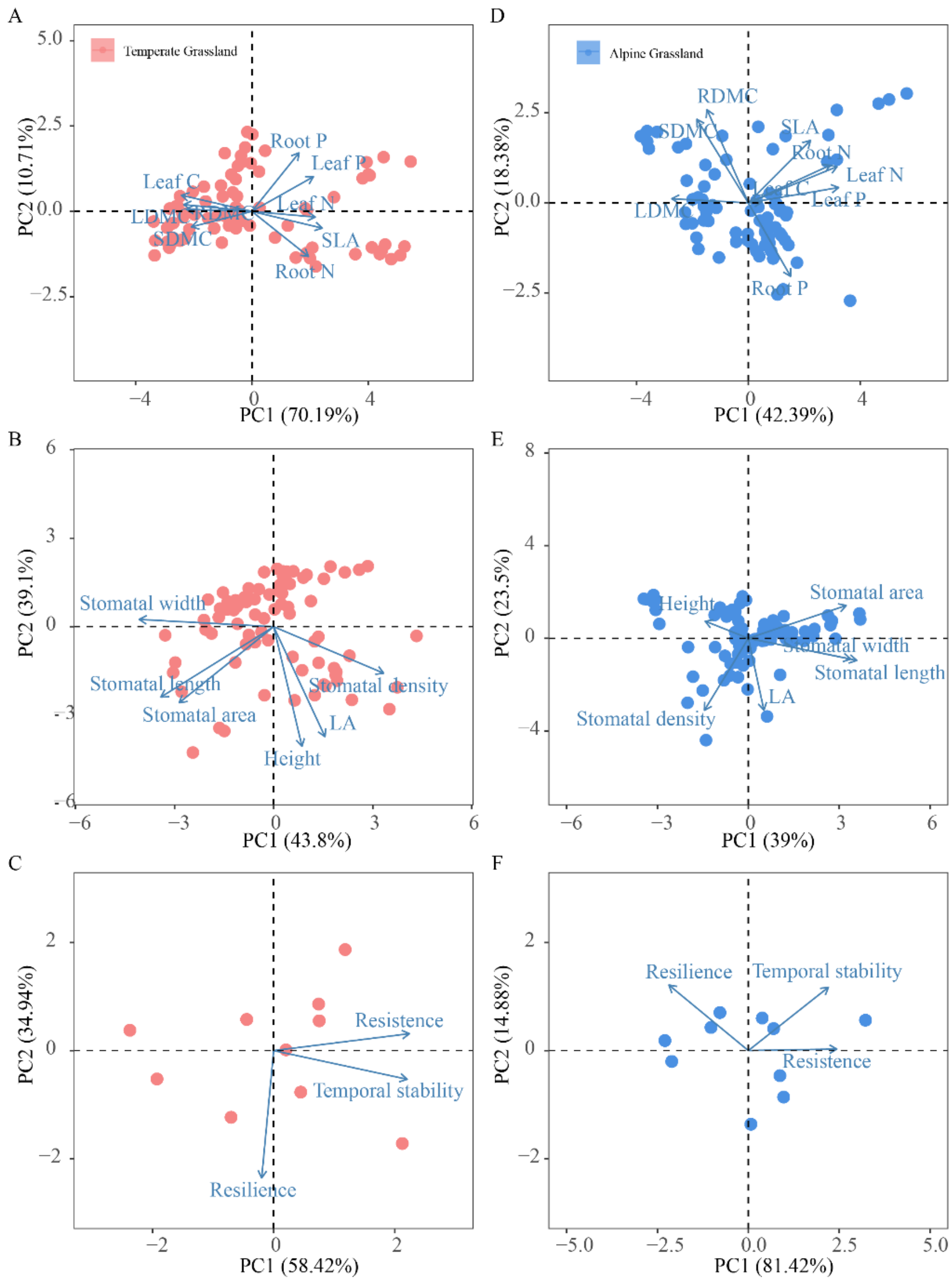

Figure S27. Results of principal component analysis of economic traits, size traits, and stability components (including temporal stability, resistance, and resilience) for temperate and alpine grasslands. Panels A–C show results for temperate grasslands, and panels D–F show results for alpine grasslands. Sample size for all panels is $n = 80$. Note that in panels C and F, overlapping points occur because EVI values are identical across the eight plots within the same site. Leaf C: Leaf carbon concentration (%); Leaf N: Leaf nitrogen concentration (%); Leaf P: Leaf phosphorus concentration (mg g^{-1}); LM: Leaf dry mass (g, single leaf); LA: Leaf area (cm^2); LDMC: Leaf dry mass content (g g^{-1}); Height: Plant height (cm); SLA: Specific leaf area (cm^2 g^{-1}); SDMC: Stem dry mass content (g g^{-1}); Root N: Root nitrogen concentration (%); Root P: Root phosphorus concentration (mg g^{-1}); RDMC: Root dry mass content (%). All variables were standardized (mean = 0, SD = 1) prior to analysis to derive standardized estimated effects.

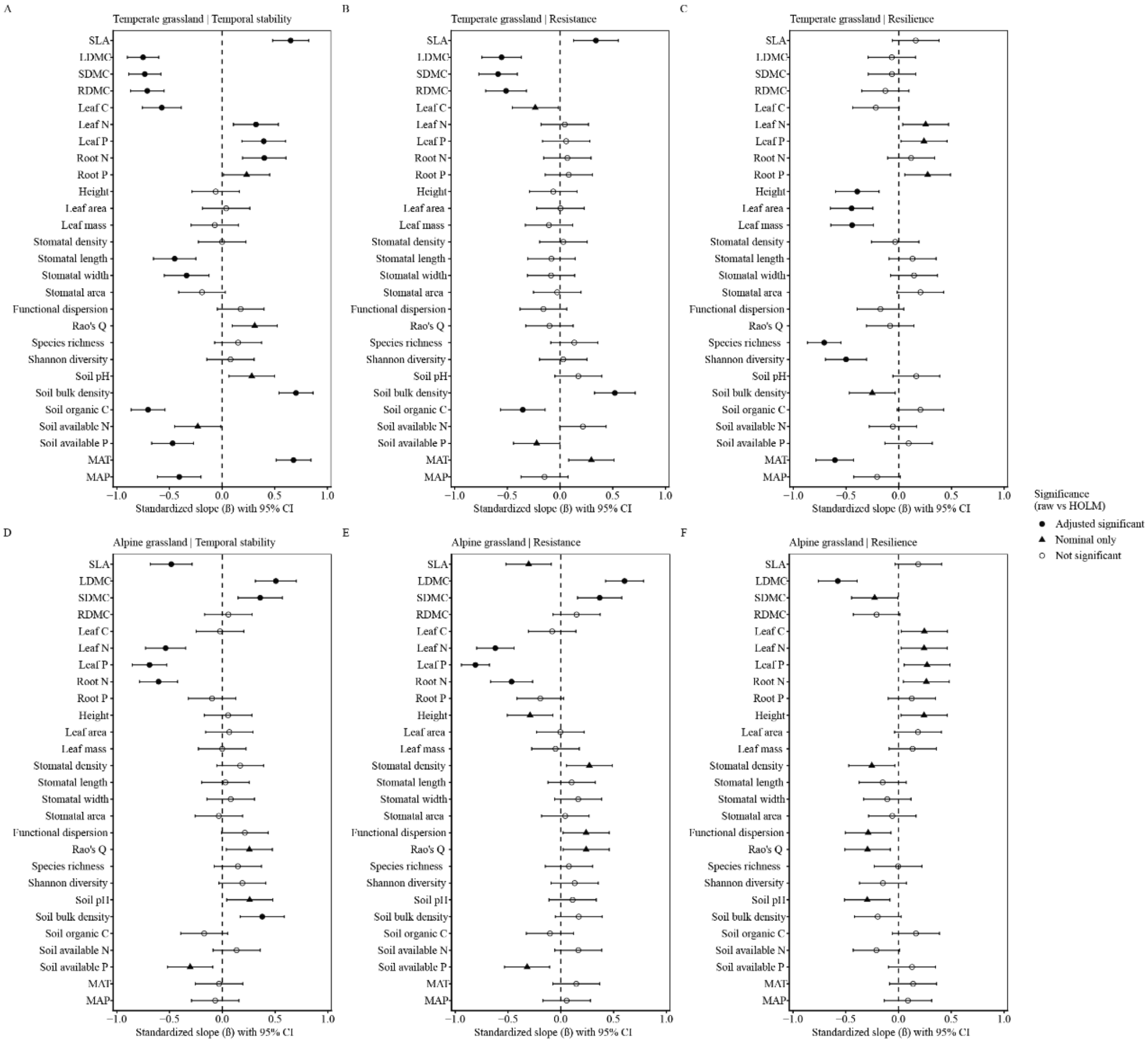

Figure S28. Sensitivity analysis of predictor–stability relationships using multiple-testing correction. Panels A–C show results for temperate grasslands, and panels D–F show results for alpine grasslands. Results are presented for temporal stability (TS; A, D), resistance (Rs; B, E) and resilience (Rl; C, F). Forest plots summarize standardized regression slopes (*β*) from separate single-predictor linear regressions relating functional traits, diversity metrics and abiotic variables to each stability component. All predictors were standardized to z-scores (mean = 0, SD = 1); stability metrics were log-transformed before standardization. Points indicate estimated slopes, and horizontal bars indicate their 95% confidence intervals. Point symbols distinguish significance before and after multiple-testing correction: solid circles (●) indicate associations that remain significant after correction (adjusted $P < 0.05$); triangles (▲) indicate associations that are significant before correction but not afterward (nominal $P < 0.05$ and adjusted $P \geq 0.05$); and open circles (○) indicate associations that are not significant before correction (nominal $P \geq 0.05$). The Holm–Bonferroni procedure was applied to P values separately within each grassland type × stability component combination, corresponding to an individual panel.

Supplementary References

1 Wang, Y. et al. Biotic stability mechanisms in Inner Mongolian grassland. Proc. R. Soc. B 287, 20200675 (2020). https://doi.org/10.1098/rspb.2020.0675

2 Jin, Y. L. et al. A plot-based dataset of plant community on the Qingzang Plateau. Chin. J. Plant Ecol. 46, 846-854 (2022). https://doi.org/10.17521/cjpe.2022.0174

3 Walkley, A. & Black, I. A. An examination of the Degtjareff method for determining soil organic matter, and a proposed modification of the chromic acid titration method. Soil Sci. 37, 29-38 (1934). https://doi.org/10.1097/00010694-193401000-00003

4 Olsen, S. R., Cole, C. V., Watanabe, F. S. & Dean, L. A. Estimation of available phosphorus in soils by extraction with sodium bicarbonate. USDA Circular No. 939 (US Government Printing Office, Washington, DC, 1954).

5 Yan, P., He, N., Yu, K., Xu, L. & Van Meerbeek, K. Integrating multiple plant functional traits to predict ecosystem productivity. Commun. Biol. 6, 239 (2023). https://doi.org/10.1038/s42003-023-04626-3

6 Kerkhoff, A. J., Enquist, B. J., Elser, J. J. & Fagan, W. F. Plant allometry, stoichiometry and the temperature-dependence of primary productivity. Glob. Ecol. Biogeogr. 14, 585-598 (2005). https://doi.org/10.1111/j.1466-822X.2005.00187.x

7 Isbell, F. et al. Biodiversity increases the resistance of ecosystem productivity to climate extremes. Nature 526, 574-577 (2015). https://doi.org/10.1038/nature15374

8 Chen, Q. et al. Multidimensional responses of grassland stability to eutrophication. Nat. Commun. 14, 6375 (2023). https://doi.org/10.1038/s41467-023-42081-0

9 Liu, S. et al. Phylotype diversity within soil fungal functional groups drives ecosystem stability. Nat. Ecol. Evol. 6, 900-909 (2022). https://doi.org/10.1038/s41559-022-01756-5
