# Supplementary material for: Contrasting plant trait strategies underpin ecosystem stability in temperate and alpine grasslands
Source: Nat Commun. 2026 Oct 8;17:11435. doi: 10.1038/s41467-026-78537-2 (PMC13737107; doi:10.1038/s41467-026-78537-2)
Supplement: Supplementary file 2 [file 41467_2026_78537_MOESM2_ESM.pdf]

nature portfolio

Corresponding author(s): Lin Jiang (lin.jiang@biology.gatech.edu)
Nianpeng He (henp@igsnrr.ac.cn)

Last updated by author(s): Aug 20, 2026

Reporting Summary

Nature Portfolio wishes to improve the reproducibility of the work that we publish. This form provides structure for consistency and transparency in reporting. For further information on Nature Portfolio policies, see our Editorial Policies and the Editorial Policy Checklist.

Statistics

For all statistical analyses, confirm that the following items are present in the figure legend, table legend, main text, or Methods section.

n/a	Confirmed	
☐	☒	The exact sample size (*n*) for each experimental group/condition, given as a discrete number and unit of measurement
☐	☒	A statement on whether measurements were taken from distinct samples or whether the same sample was measured repeatedly
☐	☒	The statistical test(s) used AND whether they are one- or two-sided<br>*Only common tests should be described solely by name; describe more complex techniques in the Methods section.*
☐	☒	A description of all covariates tested
☐	☒	A description of any assumptions or corrections, such as tests of normality and adjustment for multiple comparisons
☐	☒	A full description of the statistical parameters including central tendency (e.g. means) or other basic estimates (e.g. regression coefficient) AND variation (e.g. standard deviation) or associated estimates of uncertainty (e.g. confidence intervals)
☐	☒	For null hypothesis testing, the test statistic (e.g. *F*, *t*, *r*) with confidence intervals, effect sizes, degrees of freedom and *P* value noted<br>*Give P values as exact values whenever suitable.*
☐	☒	For Bayesian analysis, information on the choice of priors and Markov chain Monte Carlo settings
☐	☒	For hierarchical and complex designs, identification of the appropriate level for tests and full reporting of outcomes
☐	☒	Estimates of effect sizes (e.g. Cohen's *d*, Pearson's *r*), indicating how they were calculated

Our web collection on statistics for biologists contains articles on many of the points above.

Software and code

Policy information about availability of computer code

Data collection	No dedicated software or custom code was used for field data collection. Plant community, biomass, soil and trait data were recorded and measured using standardized field and laboratory protocols. Public remote-sensing and climate products were downloaded from their respective repositories and processed in R v4.5.2; dataset sources are listed in the Data Availability section.
Data analysis	All analyses were conducted in R v4.5.2. Community-weighted means, functional diversity and PERMANOVA were calculated with vegan v2.7-2; PCA with FactoMineR v2.12; Bayesian hierarchical, multivariate and structural-equation models with brms v2.23.0; convergence and posterior diagnostics with bayesplot v1.15.0; visualization with tidybayes v3.0.7 and ggplot2 v4.0.1; random forests with rfPermute v2.5.5; collinearity and model diagnostics with performance v0.15.3; and complementary d-separation diagnostics with piecewiseSEM v2.3.1. Bayesian models used four chains of 3,000 iterations (1,500 warm-up), weakly informative priors, and R-hat < 1.01 and effective sample sizes for convergence assessment. Custom R code and full session information are available at https://doi.org/10.6084/m9.figshare.27895827.

For manuscripts utilizing custom algorithms or software that are central to the research but not yet described in published literature, software must be made available to editors and reviewers. We strongly encourage code deposition in a community repository (e.g. GitHub). See the Nature Portfolio guidelines for submitting code & software for further information.

Data

Policy information about availability of data

All manuscripts must include a data availability statement. This statement should provide the following information, where applicable:

- Accession codes, unique identifiers, or web links for publicly available datasets
- A description of any restrictions on data availability
- For clinical datasets or third party data, please ensure that the statement adheres to our policy

The processed data supporting this study are deposited at Figshare (https://doi.org/10.6084/m9.figshare.27895827). Source data are provided with this paper. MOD13Q1 EVI data are available from NASA LP DAAC (https://lpdaac.usgs.gov/products/mod13q1v061/); SPEI data from the Global SPEI database (https://spei.csic.es/database.html); CRU TS climate data from the Climatic Research Unit (https://crudata.uea.ac.uk/cru/data/hrg/); and monthly potential evapotranspiration data from the Global Potential Evapo-Transpiration Climate Database (http://www.csi.cgiar.org). Species-composition data used for independent validation were obtained from the published datasets cited as Supplementary References 1 and 2.

Research involving human participants, their data, or biological material

Policy information about studies with human participants or human data. See also policy information about sex, gender (identity/presentation), and sexual orientation and race, ethnicity and racism.

Reporting on sex and gender	This study did not involve any analysis related to sex or gender.
Reporting on race, ethnicity, or other socially relevant groupings	This study did not involve any analysis related to race, ethnicity, or other socially relevant characteristics.
Population characteristics	No human participants or animals were involved; the study focused solely on plant communities in natural grassland ecosystems.
Recruitment	Not applicable. The study did not involve human participants.
Ethics oversight	Ethics approval was not required, as this study involved only plant and environmental measurements in natural ecosystems, with no human or animal participants.

Note that full information on the approval of the study protocol must also be provided in the manuscript.

Field-specific reporting

Please select the one below that is the best fit for your research. If you are not sure, read the appropriate sections before making your selection.

☐ Life sciences ☐ Behavioural & social sciences ☒ Ecological, evolutionary & environmental sciences

For a reference copy of the document with all sections, see nature.com/documents/nr-reporting-summary-flat.pdf

Life sciences study design

All studies must disclose on these points even when the disclosure is negative.

Sample size	
Data exclusions	
Replication	
Randomization	
Blinding	

Behavioural & social sciences study design

All studies must disclose on these points even when the disclosure is negative.

Study description	
Research sample	

Sampling strategy	
Data collection	
Timing	
Data exclusions	
Non-participation	
Randomization	

Ecological, evolutionary & environmental sciences study design

All studies must disclose on these points even when the disclosure is negative.

Study description	This observational comparative study tested how community functional traits relate to temporal stability, resistance and resilience in temperate and alpine grasslands in China. The main survey had a nested design of 20 sites (10 per grassland type) and 160 plots (eight 1-m2 plots per site); sites were the independent units for analyses of satellite-derived stability. We integrated 15 leaf, stem and root traits measured for 343 species with 2000-2022 EVI, climate, soil and community data. Primary site-level Bayesian analyses were supplemented by plot-level sensitivity analyses and two independent validation transects (35 temperate and 33 alpine sites).
Research sample	The main research sample comprised natural plant communities in 160 1-m2 plots across 20 sites in China (10 temperate sites on the Mongolian Plateau and 10 alpine sites on the Tibetan Plateau). The sites were selected to represent broad climatic and compositional gradients within these two major grassland types. The dataset includes 15 morphological and chemical traits measured for 343 species, plot-level community and soil data collected in 2018, and site-level EVI and climate data for 2000-2022. Independent validation used published species-composition datasets from 35 temperate and 33 alpine sites (Supplementary References 1 and 2), matched to the field-measured trait database.
Sampling strategy	No a priori power calculation was performed because this was a large-scale observational field survey constrained by standardized transect sampling and site accessibility. We used balanced replication of 10 sites per grassland type and eight plots per site (160 plots total), with sites separated by at least 80 km and spanning broad environmental gradients. Sites were the independent replicates for the primary stability analyses (n = 10 per grassland type). Independent validation with 35 temperate and 33 alpine sites addressed the limited site-level replication and tested the generality of the results.
Data collection	Trained field teams collected data during July-August 2018, at peak vegetation growth. At each site, eight 1-m2 plots were established within a relatively uniform 1,000 x 1,000 m area. Researchers recorded species presence, richness, height and cover, harvested above-ground biomass by species, and collected soil and plant material. Fifteen leaf, stem and root traits were measured using standardized protocols; replicate measurements were integrated to site-specific species-level values. Remote-sensing and climate records for 2000-2022 were obtained from public repositories. Species composition for the independent validation was obtained from two published datasets. Identical protocols and trained personnel were used across sites to minimize observer and processing variation.
Timing and spatial scale	Field surveys and trait sampling were conducted once during the peak growing season in July-August 2018. Ecosystem stability and climate metrics were derived from annual growing-season records spanning 2000-2022. The main spatial design comprised 160 1-m2 plots nested within 20 sites separated by at least 80 km across the Mongolian and Tibetan Plateaus.
Data exclusions	No sampled plots or study sites were excluded from the main analyses. For resistance and resilience calculations, consecutive climate extremes were handled using pre-specified eligibility rules described in Supplementary Text 5 so that an appropriate normal post-event season was available. In alpine structural-equation models, precipitation was omitted where necessary because of strong collinearity with other predictors; this did not exclude any observations.
Reproducibility	This was an observational study rather than a manipulative experiment. Reproducibility was evaluated through standardized field and laboratory protocols, replicate trait measurements, site-level independent replication, hierarchical analyses accounting for plots nested within sites, and sensitivity analyses at both site and plot levels. The main trait-stability patterns were independently reproduced using two additional transect datasets (35 temperate and 33 alpine sites). Additional checks using an alternative time window, biomass validation of EVI, and multiple-testing corrections yielded qualitatively consistent conclusions.
Randomization	Random allocation was not applicable because this was an observational study with no experimental treatments or assigned groups. Ten sites were distributed across each Plateau to span broad environmental gradients and were selected to minimize recent human disturbance. At each site, eight plots were established within a relatively uniform 1,000 x 1,000 m area. Analyses accounted for site-level nesting and relevant climate, soil and diversity covariates.
Blinding	Blinding was not applicable because there were no assigned treatments or subjective outcome assessments. Field variables, laboratory traits and satellite-derived stability metrics were measured or calculated using standardized, predefined procedures.

Did the study involve field work? ☒ Yes ☐ No

Field work, collection and transport

Field conditions	Fieldwork was conducted during the peak growing season in natural temperate grasslands (elevation approximately 1,000-1,200 m; mean growing-season temperature 14 degrees C; growing-season precipitation 293 mm) and alpine grasslands (elevation > 4,000 m; mean growing-season temperature 5 degrees C; growing-season precipitation 296 mm).
Location	Fieldwork was conducted at 20 sites across temperate grasslands on the Mongolian Plateau (40°–50°N, 106°–121°E, elevation ~1,000–1,200 m) and alpine grasslands on the Tibetan Plateau (25°–40°N, 74°–104°E, elevation >4,000 m), China. Sites were spaced ≥80 km apart to ensure spatial independence (see Supplementary Figure S1).
Access & import/export	Field sampling was authorized by the Institute of Geographic Sciences and Natural Resources Research, Chinese Academy of Sciences, and conformed to local governmental regulations. No biological material was transported across national borders.
Disturbance	Fieldwork involved minor disturbances through vegetation and soil sampling. Vegetation sampling required minimal removal of leaf, stem, and root material. Soil sampling may have disturbed soil structure and microbial communities.<br><br>To minimize impacts, we:<br>- Used minimally invasive sampling techniques, limiting the amount and frequency of vegetation and soil sampling.<br>- Conducted all sampling carefully to preserve vegetation integrity and soil structure.<br>- Avoided repeatedly sampling the same individual plants or locations.<br><br>Overall, disturbances were minor, localized, and transient, and are unlikely to have caused significant ecological impacts.

Reporting for specific materials, systems and methods

We require information from authors about some types of materials, experimental systems and methods used in many studies. Here, indicate whether each material, system or method listed is relevant to your study. If you are not sure if a list item applies to your research, read the appropriate section before selecting a response.

Materials & experimental systems

n/a	Involved in the study	
☒	☐	Antibodies
☒	☐	Eukaryotic cell lines
☒	☐	Palaeontology and archaeology
☒	☐	Animals and other organisms
☒	☐	Clinical data
☒	☐	Dual use research of concern
☐	☒	Plants

Methods

n/a	Involved in the study	
☒	☐	ChIP-seq
☒	☐	Flow cytometry
☒	☐	MRI-based neuroimaging

Antibodies

Antibodies used	
Validation	

Eukaryotic cell lines

Policy information about cell lines and Sex and Gender in Research

Cell line source(s)	
Authentication	
Mycoplasma contamination	
Commonly misidentified lines<br>(See ICLAC register)	

Palaeontology and Archaeology

Specimen provenance	

Specimen deposition

Dating methods

☐ Tick this box to confirm that the raw and calibrated dates are available in the paper or in Supplementary Information.

Ethics oversight

Note that full information on the approval of the study protocol must also be provided in the manuscript.

Animals and other research organisms

Policy information about studies involving animals; ARRIVE guidelines recommended for reporting animal research, and Sex and Gender in Research

Laboratory animals

Wild animals

Reporting on sex

Field-collected samples

Ethics oversight

Note that full information on the approval of the study protocol must also be provided in the manuscript.

Clinical data

Policy information about clinical studies

All manuscripts should comply with the ICMJE guidelines for publication of clinical research and a completed CONSORT checklist must be included with all submissions.

Clinical trial registration

Study protocol

Data collection

Outcomes

Dual use research of concern

Policy information about dual use research of concern

Hazards

Could the accidental, deliberate or reckless misuse of agents or technologies generated in the work, or the application of information presented in the manuscript, pose a threat to:

No	Yes	
☒	☐	Public health
☒	☐	National security
☒	☐	Crops and/or livestock
☒	☐	Ecosystems
☒	☐	Any other significant area

Experiments of concern

Does the work involve any of these experiments of concern:

No	Yes	
☒	☐	Demonstrate how to render a vaccine ineffective
☒	☐	Confer resistance to therapeutically useful antibiotics or antiviral agents
☒	☐	Enhance the virulence of a pathogen or render a nonpathogen virulent
☒	☐	Increase transmissibility of a pathogen
☒	☐	Alter the host range of a pathogen
☒	☐	Enable evasion of diagnostic/detection modalities
☒	☐	Enable the weaponization of a biological agent or toxin
☒	☐	Any other potentially harmful combination of experiments and agents

Plants

Seed stocks	No seed stocks were used. Wild plant material was collected from natural grassland plots at the 20 Mongolian and Tibetan Plateau sites during July-August 2018, following the field procedures described in the Methods and Supplementary Text 2.
Novel plant genotypes	No novel plant genotypes were generated or used.
Authentication	Not applicable because no seed stocks or novel plant genotypes were used. Species identities were recorded during standardized field community surveys.

ChIP-seq

Data deposition

☐ Confirm that both raw and final processed data have been deposited in a public database such as GEO.

☐ Confirm that you have deposited or provided access to graph files (e.g. BED files) for the called peaks.

Data access links<br>*May remain private before publication.*	
Files in database submission	
Genome browser session<br>(e.g. UCSC)	

Methodology

Replicates	
Sequencing depth	
Antibodies	
Peak calling parameters	
Data quality	
Software	

Flow Cytometry

Plots

Confirm that:

- [ ] The axis labels state the marker and fluorochrome used (e.g. CD4-FITC).
- [ ] The axis scales are clearly visible. Include numbers along axes only for bottom left plot of group (a 'group' is an analysis of identical markers).
- [ ] All plots are contour plots with outliers or pseudocolor plots.
- [ ] A numerical value for number of cells or percentage (with statistics) is provided.

Methodology

Sample preparation

Instrument

Software

Cell population abundance

Gating strategy

- [ ] Tick this box to confirm that a figure exemplifying the gating strategy is provided in the Supplementary Information.

Magnetic resonance imaging

Experimental design

Design type

Design specifications

Behavioral performance measures

Acquisition

Imaging type(s)

Field strength

Sequence & imaging parameters

Area of acquisition

Diffusion MRI ☐ Used ☐ Not used

Preprocessing

Preprocessing software

Normalization

Normalization template

Noise and artifact removal

Volume censoring

Statistical modeling & inference

Model type and settings

Effect(s) tested

Specify type of analysis: ☐ Whole brain ☐ ROI-based ☐ Both

Statistic type for inference

(See Eklund et al. 2016)

Correction

Models & analysis

n/a	Involved in the study
☐	☐ Functional and/or effective connectivity
☐	☐ Graph analysis
☐	☐ Multivariate modeling or predictive analysis

Functional and/or effective connectivity

Graph analysis

Multivariate modeling and predictive analysis
