# Supplementary material for: Contrasting plant trait strategies underpin ecosystem stability in temperate and alpine grasslands
Source: Nat Commun. 2026 Oct 8;17:11435. doi: 10.1038/s41467-026-78537-2 (PMC13737107; doi:10.1038/s41467-026-78537-2)
Supplement: Supplementary file 3 [file 41467_2026_78537_MOESM3_ESM.pdf]

nature portfolio

Peer Review File

Contrasting plant trait strategies underpin ecosystem stability in temperate and alpine grasslands

Corresponding Author: Professor Lin Jiang

This file contains all reviewer reports in order by version, followed by all author rebuttals in order by version.

Version 0:

Reviewer comments:

Reviewer #1

(Remarks to the Author)
The authors present evidence that alternate ecological strategies can promote ecosystem stability. These results are potentially and novel. However, I have a number of comments on the current version of the manuscript.

1) The goals and predictions of this study are not clear. I recommend that the authors clearly state hypotheses at the end of the introduction.

2) The authors included a large number of functional trait comparisons and should perhaps consider controlling for multiple comparisons (e.g. Holm-Bonferroni corrections).

3) The authors argue that annuals are characterised by greater resource acquisition traits than perennials (e.g. line 187). I'm not sure I agree with this interpretation as resource acquisition traits can be associated with the capacity to acquire resources in the presence of competition. If annual plants grow and reproduce prior to the onset of intense competition, then they may not need to allocate resources to traits that may increase resource acquisition in the presence of competitors. Thus annual plants express some traits associated with high resource acquisition (e.g. high SLA) but these traits are also associated with fast life histories (since low SLA is also associated with long leaf life span, up to several years, annual plants would express high SLA as part of their life history strategy). Other traits associated with resource acquisition such as high allocation to roots tend to be expressed in perennial plants, or plants experiencing below ground competition.

There are a large number of traits included in this study, the authors need to make clear how these traits are associated with resource acquisition (including in the presence of competitors) and life histories.

4) The sites used in this study are spatially related – all of the alpine sites are in one geographic area and the temperate sites are in another geographic area. Thus, other geographic specific effects (e.g. latitude, productivity, biotic interactions) could explain differences observed between these sites, perhaps in addition to the temperate versus alpine comparison. This should be addressed by the authors.

5) Line 48 – why have these aspects of ecosystems intrigued ecologists?

6) Line 52 – Clarify how biotic and abiotic factors affect ecosystem stability.

7) Lines 54-56 – How do functional traits define niches? How are response to environmental changes crucial in shaping stability?

8) Line 59-60 – What part of this relationship is fragmented and incomplete?

9) Lines 93-95 – While alpine and temperate grasslands are likely to differ in species composition, two temperate or alpine grasslands in different regions or continents would also differ in species composition.

10) Line 209 – The study to this point has largely focussed on abiotic stresses – what disturbances are the authors referring to here?

11) Lines 284-287 – Related to some of my above comments, it is not clear how each of these traits is associated with tolerance or acquisition strategies. For example, leaf area is not consistently related to resource acquisition.

12) Line 325 – what are extreme years – extreme low or high productivity?

(Remarks on code availability)
I have not reviewed the code.

Reviewer #2

(Remarks to the Author)
NCOMMS-25-78625-T
Two paths to ecosystem stability: opposing roles of plant functional traits in temperate and alpine grasslands
Thank you for the opportunity to review this manuscript 'Two paths to ecosystem stability: opposing roles of plant functional traits in temperate and alpine grasslands'. Yan and colleagues investigate trait-environment relationships and their influence on community stability across two grassland types. The study examined how plant conservative and resource-acquisitive traits mediate community responses to environmental variability (precipitation extremes) and potentially represents an important contribution to community ecology. The study uses 343 species across 20 sites (10 per grassland type) with 8 plots per site, employing ordinations and contrasts, linear models and Structural Equation Models to examine relationships between community-aggregated trait values and vegetation stability.
They calculated temporal stability, resistance and resilience using remotely sensed Enhanced Vegetation Index. EVI basically reflects greenness and standing crop. They find that trait strategies show opposing relationships in the two ecosystems. The comparison of two grassland systems (temperate and alpine) provides a valuable test of generalisability and sets up a clear contrast to examine whether functional traits influence community stability differentially across systems. The work is both novel and significant, in that it explicitly addresses inconsistency of trait-function relationships between two biomes that share some similarities (major growth forms), but differ in the climate. Such investigations are important for the field to move between context-dependent local studies and unstructured global metanalyses that blur important processes. However, methodological issues compromise the validity of the results. Most critically, the analyses fail to account for the hierarchical sampling design, treating plots as independent observations when they are nested within sites. This inflates sample sizes and leads to underestimated uncertainties. The analyses must be redone to account for this hierarchical sampling design. Importantly, there are insufficient methodological details for plant traits collection and measurements and specification of the data analyses. Moreover, the relationships between the remotely sensed EVI and biomass are not so good from Alpine grasslands. We also note lack of consideration for grazing disturbances. With revision addressing these issues, this work has the potential to make a valuable contribution to community ecology.
Major issues
• Non-independence: The study has 20 sites with 8 plots each, but all analyses (apparently) treat plots as independent observations. Plots within sites are not independent. All analyses must be redone to account for study design.
• Line 339-344: Is the PCA performed on plots or species? If on plots, did the authors account for site-level dependence? Clarify and justify and/or rectify.
• Lines 345-392: Bayesian linear and structural equation models apparently lack hierarchical structure. The models do not appear to account for the sampling design or plot dependence within sites. Models must include random effects for sites. The manuscript should provide: (1) complete model formula in R syntax or mathematical notation showing hierarchical structure, (2) explicit prior specifications for all parameters with justification, (3) model family with reasoning, (4) convergence diagnostics, and (5) brms package version.
• Lines 303-321: Insufficient justification for wet/dry thresholds. Why were SPI wet/dry thresholds specifically chosen? Why not temperature extremes? Provide clear ecological justification for threshold selection appropriate to these grassland ecosystems. Furthermore, citations for resistance and resilience indices are from soil biota papers (line 321). Explain why these methods are appropriate for plant communities or cite relevant plant ecology references.
• Lines 292-293, 302, 317: Climate and imagery data span 2000-2022, but plant surveys and trait measurements were in 2018. Why extend climate/imagery data to 2022, four years after trait sampling? Using data from years after trait measurements could lead to spurious correlations. Justify this temporal scope or revise analysis to use 2000-2018 data.
• Supplementary Materials Figure S13: The relationship between biomass and EVI is not compelling, particularly for alpine grassland. At high biomass levels, there is very little predictive capacity. This raises questions about using EVI as a proxy for biomass in stability calculations. Either justify why EVI is appropriate despite weak relationships or acknowledge as a limitation.
• Grazing disturbances not addressed: Worldwide, grasslands are historically heavily grazed and grazing intensity may vary with elevation and through time. The manuscript states sites have "no human disturbance," but provides no evidence of other disturbance types. Either quantify grazing at both sites and control for it in analyses, provide evidence that disturbance was minimal and equivalent across sites, or acknowledge as a limitation.
• Lines 280-290: Insufficient trait collection detail. Simply citing Pérez-Harguindeguy et al. is inadequate. Provide explicit details on collection and measurement protocols, including number of plants sampled per species, number of species per plot/site/grassland type, number of replicates per trait measurement and method of trait measurement. Include complete details in supplementary materials if space is limited.
• Lines 292-294, 303: Climate data spatial resolution unclear. For example, climate data from China Meteorological Data Service Centre appears very broad-scale. How were site-specific values derived? Specify exact data source, spatial resolution, how site-specific values were obtained/interpolated, justification for site-level climate being applied to plot-level analyses.

Minor Issues
Introduction:
• Difficult to understand results without reading methods and supplementary materials. It is suggested to expand explanation of community stability concepts and improve logical flow, particularly for transition from traits to ecosystem stability to grasslands is disjunct at Line 90.
• Line 98: Introduction mentions 15 traits; methods state 16. Correct and ensure consistency throughout.
Methods:
• Lines 281-282: Wording implies authors used existing dataset rather than collecting new data. Reword for clarity.
• Lines 284-286: Define all acronyms before first use (write out full terms first, then acronym in parentheses).
• Line 315: Missing citations for rationale for EVI metric.
• Missing R package citations and versions. Cite all R packages used with version numbers (brms, vegan, etc). Additionally, add R version session at the end of R code. Essential for reproducibility.
Supplementary Materials:
• Lines 268, 272 (and throughout): Incorrect supplementary references. Lines 268 and 272 refer to Table S1 and Figure S1 when they should be Table S2 and Figure S2. Systematically check and correct all supplementary citations.
• Supplementary materials labelling: Figure, table, and text labels are mixed up (e.g., Text 2 should be Text 3). Supplementary Text 3 lacks references for growing season calculations. Several supplementary figures are not described in main manuscript, either describe or remove them.
Figures and references:
• Figure quality: Many figures have poor resolution, overlapping text, and cut-off elements (e.g., bottom arrow in Figure 1). Ensure all figures meet journal quality standards.
• Reference formatting: Inconsistent formatting, some references missing DOIs. Ensure consistent style per journal guidelines and include DOIs where available.
• Permits: Confirm appropriate permits were obtained for plant biomass removal from both sites and state permit number.

(Remarks on code availability)
In a partial review, two issues were noted.
1) it appears that the sampling design of plots nested within sites is not reflected in the analyses.
2) analyses produced interim data objects that the researchers apparently saved on their local drives. these were subsequently called and read by the code. For an independent analyst, these scripts could be run, but required significant interaction to reset interim data object names.

Reviewer #3

(Remarks to the Author)
I co-reviewed this manuscript with one of the reviewers who provided the listed reports. This is part of the Nature Communications initiative to facilitate training in peer review and to provide appropriate recognition for Early Career Researchers who co-review manuscripts.

(Remarks on code availability)

Version 1:

Reviewer comments:

Reviewer #1

(Remarks to the Author)
The authors generally made appropriate responses to my previous review. I have several comments on the revised manuscript.

1) I remain uncertain about how the authors (and others in this field) link functional traits to ecological strategies such as stress tolerance. While traits associated with resource conservation (e.g. low SLA) can be associated with stress tolerance, they are also observed in low stress environments such as rainforests. While these environments are low stress, they tend to favour long-life spans and functional traits that are often associated with resource conservation. Perhaps this could be addressed in the manuscript by limiting the discussion to grasslands on environmental gradients, as these ecosystems are functionally similar and functional differences are likely due to differences in abiotic stress.

2) The key finding of this study is that conservative and acquisitive strategies can promote ecosystem stability. Is there any way we can predict which of these strategies is more likely to promote stability in a given ecosystem?

3) In the alpine ecosystem traits associated with resilience are opposite to those that are associated with resistance. Is resilience or resistance more important for ecosystem stability?

4) Line 189 – “to our knowledge, this is the first empirical evidence …” – can the authors confirm that they have done an appropriate search to support this statement?

5) Line 193-195 – add a reference for this statement?

6) Lines 259-268 – add appropriate references.

(Remarks on code availability)

Reviewer #2

(Remarks to the Author)
Thank you for the opportunity to review the revised manuscript. The authors have made a commendable effort in addressing most of the concerns raised in the first round of review, and several comments have been resolved satisfactorily. However, several serious concerns remain that must be addressed before the manuscript can be considered for publication. In particular, outstanding issues regarding pseudoreplication, model presentation, and reporting require attention. Below we outline the major issues that remain to be addressed. Please note that the merged pdf document and the word document do not have the same content. Here we refer to the word document.
Non-independence and hierarchical structure in models
Thank you for including site as a random effect; this is the appropriate approach for accounting for pseudoreplication. However, the plot-level random effects models continue to be presented as the main results throughout the manuscript. Doing so will lead to incorrectly estimating uncertainty around the parameters. This is a serious methodological concern, particularly for the SEMs. The plot-level models should not be presented as primary results. Furthermore, the methods section currently describes both the site-level and plot-level random effects models, which is confusing for the reader. We recommend removing the plot-level random effects model from the methods entirely; if the authors wish to retain this analysis, it should be moved to the supplementary material.
Additionally, the raw R syntax in the manuscript text is not appropriate for a scientific publication (e.g. Method section, lines 799-806, lines 809-810). The model structure is still insufficiently explicit, and certain models, particularly the STMs, continue to be presented without adequate justification. Where a model formula is included, please ensure it is written correctly and that all terms, including sigma, are explicitly defined (e.g. Method section, lines 809-810).
Finally, the term "robust" should be removed when describing the Bayesian models in the methods section, as this terminology is unnecessary and imprecise in this context.
Citations for packages
Please ensure that R packages are cited correctly throughout the manuscript. For example, the brms package should be cited as: Burkner, P.C. (2017). brms: An R package for Bayesian multilevel models using Stan. Journal of Statistical Software, 80(1), 1-28. https://doi.org/10.18637/jss.v080.i01
Code
We were unable to fully review the code; however, if the authors have ensured that the model structure is clearly described and that the code is reproducible for independent analysts, this is satisfactory.

(Remarks on code availability)
The code was reviewed on first submission.

Reviewer #3

(Remarks to the Author)
I co-reviewed this manuscript with one of the reviewers who provided the listed reports. This is part of the Nature Communications initiative to facilitate training in peer review and to provide appropriate recognition for Early Career Researchers who co-review manuscripts.

(Remarks on code availability)

Version 2:

Reviewer comments:

Reviewer #1

(Remarks to the Author)
The authors have made appropriate responses and revisions to the comments in the previous review. I have no further comments.

(Remarks on code availability)

Reviewer #2

(Remarks to the Author)

We appreciate the efforts of the authors to respond positively to our comments. The paper is an interesting and robust analysis of how traits may acti differentially to influence ecosystem stability in different environments. Well done and good luck.

(Remarks on code availability)

Reviewer #3

(Remarks to the Author)
I co-reviewed this manuscript with one of the reviewers who provided the listed reports. This is part of the Nature Communications initiative to facilitate training in peer review and to provide appropriate recognition for Early Career Researchers who co-review manuscripts.

(Remarks on code availability)

Open Access This Peer Review File is licensed under a Creative Commons Attribution 4.0 International License, which permits use, sharing, adaptation, distribution and reproduction in any medium or format, as long as you give appropriate credit to the original author(s) and the source, provide a link to the Creative Commons license, and indicate if changes were made.
In cases where reviewers are anonymous, credit should be given to 'Anonymous Referee' and the source.
The images or other third party material in this Peer Review File are included in the article's Creative Commons license, unless indicated otherwise in a credit line to the material. If material is not included in the article's Creative Commons license and your intended use is not permitted by statutory regulation or exceeds the permitted use, you will need to obtain permission directly from the copyright holder.
To view a copy of this license, visit https://creativecommons.org/licenses/by/4.0/

Response to the Reviewers:

(Original comment and query are in italics and key comments are in bold; responses are in blue font)

Reviewer 1:

Reviewer #1 (Remarks to the Author):

The authors present evidence that alternate ecological strategies can promote ecosystem stability. These results are potentially and novel. However, I have a number of comments on the current version of the manuscript.

1) The goals and predictions of this study are not clear. I recommend that the authors clearly state hypotheses at the end of the introduction.

Response: Thank you for this valuable suggestion. We agree that our goals and predictions should be stated more explicitly. We have therefore revised the final paragraph of the Introduction to clearly articulate our study objectives and a priori hypotheses. Specifically, we now state that we test whether community functional strategies (conservative vs. acquisitive) predict multiple stability dimensions (temporal stability, resistance, and resilience) and whether these trait–stability relationships differ between temperate and alpine grasslands. Based on the contrasting environmental constraints and life-history composition of the two systems, we predict that conservative strategies will be more strongly associated with temporal stability and resistance in alpine grasslands (where cold stress and shorter growing seasons favor stress tolerance), whereas acquisitive strategies will be more strongly associated with temporal stability and resistance in temperate grasslands (where longer growing seasons and higher annual prevalence favor fast growth and rapid turnover), and that acquisitive strategies will be positively related to resilience in both systems. These hypotheses are now explicitly stated at the end of the Introduction (see revised **lines 119–127**).

"We hypothesized that the relative importance of conservative versus acquisitive plant strategies for ecosystem stability would differ between temperate and alpine grasslands. Specifically, we predicted that in the cold, resource-limited alpine grasslands, traits associated with resource conservation would be more strongly linked to resistance and temporal stability, whereas in the warmer, more productive temperate grasslands, traits associated with resource acquisition would play a greater role in regulating these stability components. In contrast, we expected acquisitive strategies to be more closely associated with resilience in both systems by facilitating rapid regrowth following climatic extremes."

2) The authors included a large number of functional trait comparisons and should perhaps consider controlling for multiple comparisons (e.g. Holm-Bonferroni corrections).

Response: Thank you for this helpful suggestion. We agree that testing many traits

simultaneously can inflate Type I error. Because our primary inference is Bayesian (based on posterior distributions rather than P-value thresholds), classical P-value adjustments are not directly part of the main analysis. To address this concern, we conducted a parallel sensitivity analysis using frequentist univariate regressions and applied Holm correction within each region × stability-component family of trait tests. Only a small number of trait–stability associations lost statistical support after adjustment, and the key conclusion remains unchanged: temporal stability and resistance align with more acquisitive trait syndromes in temperate grasslands, but with more conservative syndromes in alpine grasslands. We now report nominal and Holm-adjusted P values in the Supplementary Information (Figure S25) and note this robustness check in the Methods (**lines 542-545**).

"Because we evaluated multiple traits, we additionally performed a sensitivity analysis in which we fitted univariate frequentist regressions and applied Holm correction to P values within each region × stability-component family of tests; the main results were unchanged (Figure S25)."

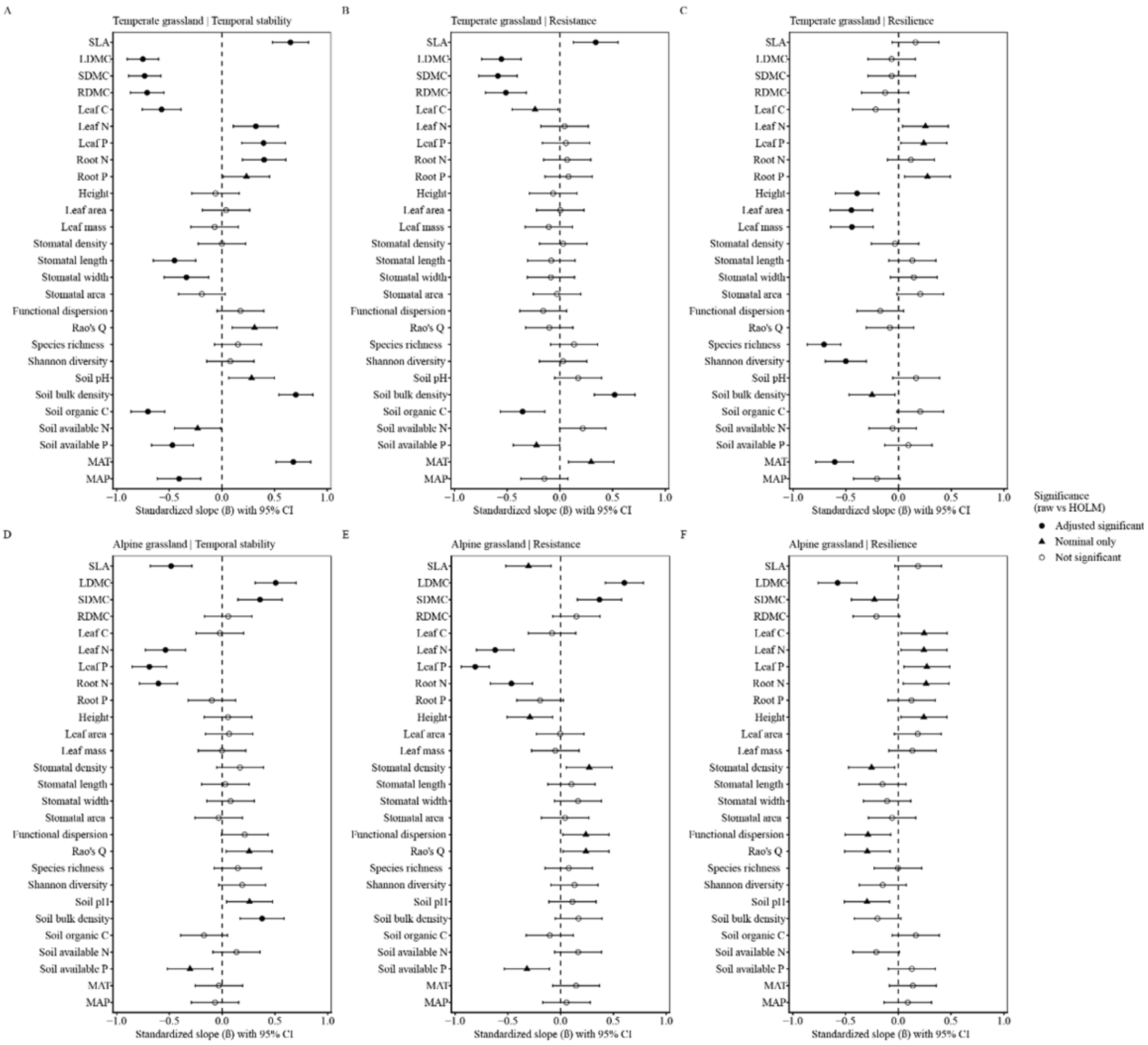

Figure S25. Sensitivity analysis of trait–stability relationships using multiple-testing corrections. Forest plots summarize standardized slopes (***β***) ± 95% confidence intervals derived from univariate linear regressions (frequentist

framework) of individual predictors—including functional traits, diversity, and abiotic variables—against three ecosystem stability dimensions: temporal stability (TS), resistance (Rs), and resilience (Rl). Results are partitioned by region (temperate vs. alpine grasslands) and stability dimension. All variables were z-score standardized; stability metrics were log-transformed prior to standardization. Points and horizontal bars represent estimated β and their 95% confidence intervals, respectively. To assess the robustness of these associations, significance is indicated by point symbols based on both nominal and adjusted ***P***-values: solid circles (●) denote effects remaining significant after multiple-testing correction (***P*** < 0.05); triangles (▲) denote nominal significance (***P*** < 0.05) that becomes non-significant after adjustment (***P*** ≥ 0.05); and open circles (○) denote non-significant effects (***P*** ≥ 0.05). Multiple-testing correction was performed using the Holm–Bonferroni procedure, applied independently within each region × stability dimension combination (panel-wise adjustment).

3) The authors argue that annuals are characterised by greater resource acquisition traits than perennials (e.g. line 187). I'm not sure I agree with this interpretation as resource acquisition traits can be associated with the capacity to acquire resources in the presence of competition. If annual plants grow and reproduce prior to the onset of intense competition, then they may not need to allocate resources to traits that may increase resource acquisition in the presence of competitors. Thus annual plants express some traits associated with high resource acquisition (e.g. high SLA) but these traits are also associated with fast life histories (since low SLA is also associated with long leaf life span, up to several years, annual plants would express high SLA as part of their life history strategy). Other traits associated with resource acquisition such as high allocation to roots tend to be expressed in perennial plants, or plants experiencing below ground competition.

Response: We thank the reviewer for this important conceptual clarification. We agree that "resource acquisition" can refer to different ecological dimensions. In the plant economics spectrum, acquisitive traits such as high SLA, high leaf N and rapid tissue turnover reflect a fast return on carbon investment and short tissue lifespan rather than competitive dominance in resource capture under crowding. Annual species typically occupy the fast end of this spectrum, investing in inexpensive, short-lived tissues that support rapid growth and reproduction early in the season. This strategy allows annuals to exploit favorable conditions and often avoid intense competition, rather than to outcompete neighbors via sustained investment in resource-capturing structures.

We also agree that traits more directly associated with competitive resource acquisition in the presence of neighbors, such as large and persistent root systems, storage organs, and long-lived tissues, are more characteristic of perennial species. We have therefore revised the text to clarify that annuals are "acquisitive" in the sense of fast tissue economics and rapid resource turnover, rather than in the sense of superior competitive ability for resource capture. (see revised **lines 207–218**).

"While the shorter growing season and lower temperatures in alpine grasslands favor stress-tolerant, resource-conservative perennial species [39], temperate grasslands, with longer growing season and milder climate, support a higher proportion of annual plants. Our data indicate that annuals tend to exhibit faster trait syndromes (e.g., large SLA; see Figure S14) [40,41], consistent with rapid growth and turnover as part of a fast life-history strategy, which is classified as acquisitive in the context of the plant economics spectrum. Such fast strategies may also reflect phenological "escape" from peak competition, whereas investments linked to resource capture under crowding can be more pronounced in perennials. Annuals therefore contributed significantly to increased community-level acquisitive issue economics in our study, particularly in temperate grasslands (see Figure S15 and S16)."

There are a large number of traits included in this study, the authors need to make clear how these traits are associated with resource acquisition (including in the presence of competitors) and life histories.
Response: Thank you for this comment. We agree that, given the large number of traits, it is essential to clarify their functional interpretation. We have revised the Methods to explicitly distinguish among (***i***) traits reflecting resource-acquisition potential and fast tissue turnover versus conservative investment and stress tolerance, (***ii***) traits primarily capturing competitive ability under crowding (e.g., height and leaf area as proxies for size- and light-competition, rather than direct measures of acquisition), and (***iii***) traits linked to life-history variation (e.g., rapid turnover and short reproductive cycles that often accompany acquisitive strategies). To avoid over-interpreting any single trait in isolation, we emphasize PCA-derived trait axes that summarize coordinated trait covariation (Figures 1). PCA loadings and trait–trait correlations are provided in the Supplementary Information (**Figure S24**).

Revised Methods text (**lines 344-354**):

"These traits include plant height (cm), leaf area (LA, cm^2), which primarily reflect plant size and competitive ability for light; leaf carbon concentration (Leaf C, %); leaf nitrogen concentration (Leaf N, %); leaf phosphorus concentration (Leaf P, mg g^{-1}); root nitrogen concentration (Root N, %); root phosphorus concentration (Root P, mg g^{-1}), together with specific leaf area (SLA, cm^2 g^{-1}), which are commonly associated with resource-acquisition potential and fast tissue turnover; leaf, stem and root dry matter content (LDMC, SDMC, RDMC, %), which reflect tissue investment and are often linked to conservative strategies and stress tolerance; and stomatal length, stomatal width, stomatal area, and stomatal density, which indicate leaf gas-exchange capacity and potential sensitivity to water and temperature conditions (Table S2)."

4) The sites used in this study are spatially related – all of the alpine sites are in one geographic area and the temperate sites are in another geographic area. Thus, other geographic specific effects (e.g. latitude, productivity, biotic interactions) could explain differences observed between these sites, perhaps in addition to the temperate

versus alpine comparison. This should be addressed by the authors.
Response: We agree that the alpine and temperate sites are geographically clustered, and that spatially structured factors (e.g., latitude, productivity, and broader ecological context) could contribute to the observed contrasts. To address this concern, we performed a sensitivity analysis refitting the trait–stability models with latitude and aboveground biomass (a field-based proxy for productivity) included as covariates. The estimated effects of the economic-trait axis on temporal stability, resistance, and resilience—and the contrast between grassland types—remained qualitatively unchanged (Figure S17), indicating that our main conclusions are unlikely to be explained by simple latitudinal or productivity gradients alone.

With respect to biotic interactions (e.g., herbivory, pathogen pressure, or competitive intensity), we do not have standardized, site-consistent measurements across all sites, and therefore cannot incorporate these processes quantitatively. We have added a brief discussion acknowledging this limitation and noting that such interactions could, in principle, affect trait–stability relationships; accordingly, we interpret grassland type as a broader ecosystem context and highlight coordinated measurements of biotic interactions as an important direction for future work (revised **lines 275–286**).

"Furthermore, because our temperate and alpine grasslands are located in different geographic regions, the grassland-type contrast may also reflect spatially structured context that covaries with location (e.g., latitude gradients and regional productivity regimes). However, models controlling for latitude and aboveground biomass (as a proxy of productivity) yielded qualitatively similar trait–stability relationships (Figure S17), suggesting that our conclusions are not driven solely by broad geographic gradients. We further acknowledge that biotic interactions (e.g., herbivory, pathogen pressure, competitive intensity), which were not measured in our study, may vary among sites and could influence trait–stability relationships. Here, we treat grassland type as an integrated ecosystem context and encourage future studies that incorporate coordinated measurements of these processes to more explicitly assess their roles in shaping trait-stability linkages."

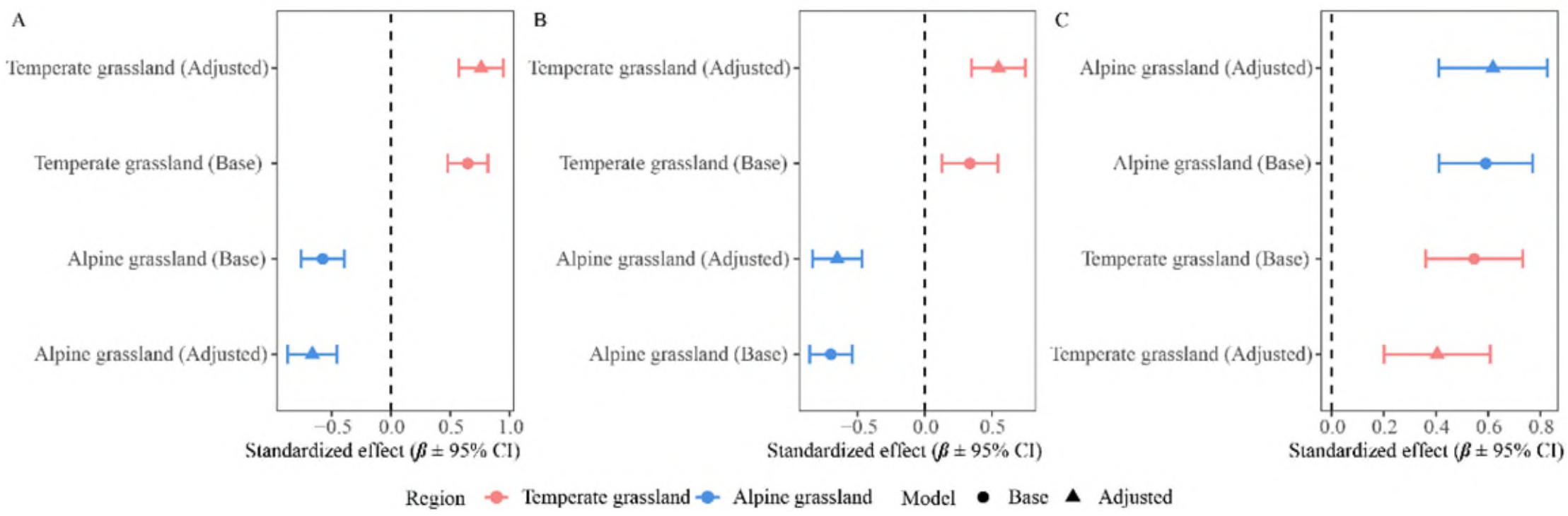

Figure S17. Economic-trait effects on ecosystem stability are robust to controlling for latitude and productivity across temperate and alpine grasslands. Panels A–C show standardized regression coefficients (β) and 95% confidence

intervals for the effects of economic traits on temporal stability (A), resistance (B), and resilience (C). Points indicate model estimates (***β***) and horizontal bars denote 95% CIs; the dashed vertical line marks no effect (***β*** = 0). Colors denote grassland type (red = temperate, blue = alpine). Point shapes distinguish model specifications: Base models include economic traits only, whereas Adjusted models additionally include latitude and aboveground biomass (as a productivity proxy). Higher values of economic traits indicate a more acquisitive strategy.

5) Line 48 – why have these aspects of ecosystems intrigued ecologists?
Response: We clarified that ecologists have long been intrigued by ecosystem stability because it determines how reliably ecosystems sustain key functions and services (e.g., productivity and carbon uptake) under environmental variability and disturbance (**see revised lines 44–51**).

"Understanding the determinants of ecosystem stability, which encompasses multiple dimensions such as temporal stability (the extent to which a system remains invariant over time), resistance (the capacity of a system to withstand perturbations), and resilience (the ability of a system to return to its original state following perturbations), has long intrigued ecologists [1-4]. These stability properties characterize the ability of ecosystems to maintain their functions and services in the face of environmental change and variability, are thus of fundamental importance to humanity."

6) Line 52 – Clarify how biotic and abiotic factors affect ecosystem stability.
Response: We revised the text to explicitly distinguish the roles of abiotic and biotic drivers of ecosystem stability. The revision clarifies that abiotic variation affects stability by directly influencing plant performance and temporal variability in ecosystem functioning, whereas biotic factors shape stability by altering community-level responses to environmental change(**see revised lines 53–58**).

"Substantial research has shown that ecosystem stability is affected by both abiotic (e.g., climate) and biotic (e.g., species diversity) factors [6-12]. Variation in abiotic conditions may alter plant physiology, phenology, and growth, thereby driving temporal changes in ecosystem functioning (e.g., productivity) [7,13]. Biotic factors may further influence stability by modifying how species respond to environmental change [14]."

7) Lines 54-56 – How do functional traits define niches? How are response to environmental changes crucial in shaping stability?
Response: We revised the text to more clearly articulate the link between functional traits, species niches, and ecosystem stability by emphasizing the role of traits in governing plant performance and responses to environmental change, and how these trait-based differences translate into patterns of ecosystem resistance, resilience, and temporal stability (**see revised lines 59–64**).

"Indeed, functional traits define species niches [15] by regulating how plants perform under various abiotic and biotic conditions, thereby determining where they can persist and how they interact with co-occurring species. Functional traits also regulate species' sensitivities to environmental change and subsequent recovery[14,16], which, in turn, determine resistance, resilience and temporal stability of ecosystems."

8) Line 59-60 – What part of this relationship is fragmented and incomplete?
Response: We clarified that "fragmented and incomplete" refers to the structure of the existing evidence base: studies often examine isolated traits or focus on a single dimension of stability, and are typically conducted within limited ecological contexts. As a result, trait–stability evidence is dispersed across traits, stability dimensions, and study systems, which makes it difficult to synthesize and compare results across ecosystems (see revised **lines 66–69**).

"However, despite much progress [6,10,17-21], our understanding of trait-stability relationships remains fragmented and incomplete, as studies often examine isolated traits or single dimensions of stability within limited ecological contexts, limiting synthesis across systems."

9) Lines 93-95 – While alpine and temperate grasslands are likely to differ in species composition, two temperate or alpine grasslands in different regions or continents would also differ in species composition.
Response: Thank you for this helpful comment. We agree that species composition can vary substantially among grasslands even within the same biome across different regions or continents. Our goal was not to suggest that "temperate" versus "alpine" universally implies a single, consistent compositional contrast everywhere, but rather to describe the specific, repeatable abiotic and biotic differences between the two grassland systems examined in this study (temperate grasslands on the Mongolian Plateau vs. alpine grasslands on the Tibetan Plateau). We have revised the Introduction to explicitly anchor the comparison to our study regions and to frame these contrasts as a context gradient for testing whether trait–stability relationships vary across ecosystems (revised **lines 107–112**). In addition, we added text in the Discussion to clarify that our inference concerns broad environmental and life-history contrasts rather than biome identity per se (revised **lines 269–275**).

Revised text (Introduction; revised **lines 107–112**):
"In our study regions, these climate contrasts are mirrored by differences in species compositions: temperate grasslands contain a higher proportion of fast-growing annual plants, while alpine grasslands are dominated by slow-growing perennial species adapted to harsh environments [34]. Together, these consistent climate and biotic contrasts between the two grasslands types provide a valuable opportunity for testing whether trait–stability relationships depend on ecosystem context."

Added clarification (Discussion; revised **lines 269–275**):
"In this study, we compared temperate and alpine grasslands to represent two broadly contrasting ecosystem types with distinct climatic and biological characteristics. We acknowledge that differences in species composition are not unique to this contrast, as grasslands within the same biome but in different regions can also differ substantially in floristic composition; thus, our comparison should be interpreted as capturing broad environmental and life-history differences rather than isolating biome identity per se."

10) Line 209 – The study to this point has largely focussed on abiotic stresses – what disturbances are the authors referring to here?
Response: Thank you for raising this point. In our analyses, "disturbances" refer specifically to climatic perturbations—i.e., SPEI-defined extreme dry and wet events (drought/wet anomalies) that drive strong departures in vegetation productivity (EVI) and underpin our resistance and resilience metrics. We have revised the text to explicitly state that we are referring to climate extremes (rather than disturbances such as grazing or fire, which were not investigated in this study) (**see revised lines 235–238**).

"These traits allow them to effectively exploit temporarily increased resources that often follow climatic perturbations (e.g., precipitation anomalies) [43], enabling ecosystems dominated by these plants to restore functions more quickly after climate extremes [44]."

11) Lines 284-287 – Related to some of my above comments, it is not clear how each of these traits is associated with tolerance or acquisition strategies. For example, leaf area is not consistently related to resource acquisition.
Response: Thank you for this helpful comment. We agree that several traits (e.g., leaf area) are not consistently or uniquely linked to a single acquisition–tolerance axis. To make our interpretation clearer, we revised the Methods text to explicitly (***i***) distinguish traits that primarily reflect plant size/competitive ability (e.g., height, leaf area) from those more directly associated with resource acquisition and fast tissue turnover (e.g., SLA and nutrient concentrations) versus conservative investment and stress tolerance (e.g., dry matter contents), and (***ii***) clarify that our inference is based on multivariate trait syndromes captured by PCA, rather than assigning each trait to a strategy in isolation (**see revised lines 344–354**).

"These traits include plant height (cm), leaf area (LA, cm^2), which primarily reflect plant size and competitive ability for light; leaf carbon concentration (Leaf C, %); leaf nitrogen concentration (Leaf N, %); leaf phosphorus concentration (Leaf P, $mg\ g^{-1}$); root nitrogen concentration (Root N, %); root phosphorus concentration (Root P, $mg\ g^{-1}$), together with specific leaf area (SLA, $cm^2\ g^{-1}$), which are commonly associated with resource-acquisition potential and fast tissue turnover; leaf, stem and root dry matter content (LDMC, SDMC, RDMC, %), which reflect tissue investment and are often linked to conservative strategies and stress tolerance; and stomatal length,

stomatal width, stomatal area, and stomatal density, which indicate leaf gas-exchange capacity and potential sensitivity to water and temperature conditions (Table S2)."

12) Line 325 – what are extreme years – extreme low or high productivity?
Response: Thank you for the clarification request. In our study, "extreme years" are defined by climatic extremes (dry or wet anomalies) rather than by extreme low or high productivity per se. Specifically, extreme years were identified using the SPEI classification (i.e., drought vs wet anomalies based on threshold values; see Methods/Text S2). Accordingly, $\boldsymbol{Productivity}_{extreme}$ refers to the observed ecosystem productivity (EVI) during those climate-extreme years, which can be lower in drought years and higher in unusually wet years, depending on the type of extreme (**see revised lines 436–437**).

"$\boldsymbol{Productivity}_{extreme}$ represents ecosystem productivity in climate-extreme years (drought or wet anomalies defined by extreme SPEI values)"

Reviewer #1 (Remarks on code availability):

I have not reviewed the code.

Reviewer 2:

Reviewer #2 (Remarks to the Author):

NCOMMS-25-78625-T
Two paths to ecosystem stability: opposing roles of plant functional traits in temperate and alpine grasslands
Thank you for the opportunity to review this manuscript 'Two paths to ecosystem stability: opposing roles of plant functional traits in temperate and alpine grasslands'. Yan and colleagues investigate trait-environment relationships and their influence on community stability across two grassland types. The study examined how plant conservative and resource-acquisitive traits mediate community responses to environmental variability (precipitation extremes) and potentially represents an important contribution to community ecology. The study uses 343 species across 20 sites (10 per grassland type) with 8 plots per site, employing ordinations and contrasts, linear models and Structural Equation Models to examine relationships between community-aggregated trait values and vegetation stability.
They calculated temporal stability, resistance and resilience using remotely sensed Enhanced Vegetation Index. EVI basically reflects greenness and standing crop. They find that trait strategies show opposing relationships in the two ecosystems. The comparison of two grassland systems (temperate and alpine) provides a valuable test of generalisability and sets up a clear contrast to examine whether functional traits influence community stability differentially across systems.
The work is both novel and significant, in that it explicitly addresses inconsistency of trait-function relationships between two biomes that share some similarities (major

growth forms), but differ in the climate. Such investigations are important for the field to move between context-dependent local studies and unstructured global metanalyses that blur important processes. However, methodological issues compromise the validity of the results. Most critically, ***the analyses fail to account for the hierarchical sampling design****, treating plots as independent observations when they are nested within sites. This inflates sample sizes and leads to underestimated uncertainties.* ***The analyses must be redone to account for this hierarchical sampling design****. Importantly, there are insufficient methodological details for plant traits collection and measurements and specification of the data analyses. Moreover, the relationships between the remotely sensed* ***EVI and biomass are not so good from Alpine grasslands****. We also note lack of consideration for grazing disturbances. With revision addressing these issues, this work has the potential to make a valuable contribution to community ecology.*

Major issues

- *Non-independence: The study has 20 sites with 8 plots each, but all analyses (apparently) treat plots as independent observations. Plots within sites are not independent. All analyses must be redone to account for study design.*

Response: This is an important point and we appreciate the opportunity to clarify our analytical treatment of non-independence. We agree that plots are nested within sites and therefore are not statistically independent. We have updated our analyses accordingly wherever the nested structure is informative and estimable—specifically, we now use Bayesian hierarchical (mixed-effects) models with a site-level random intercept when the response varies among plots within a site (e.g., **Table S2**). Importantly, these updates yield results that are essentially unchanged from the original analyses, and our main conclusions remain the same.

However, it is also important to note that our stability metrics—EVI-based temporal stability, resistance, and resilience—are derived from a common remote-sensing time series for each site. Consequently, these metrics primarily reflect site-level ecosystem dynamics and do not meaningfully differ among plots within the same site, making mixed-effects models with a site random intercept unnecessary for these analyses. To address the reviewer's concern under this data structure, **we re-ran the corresponding analyses at the site level,** using site-aggregated predictors where appropriate. The **site-level results were qualitatively consistent with the plot-level patterns**, although statistical power was reduced due to the smaller sample size (n = 10 per grassland type; **Figures S8; Table S5**). In other words, plot-level analyses may overstate nominal degrees of freedom, whereas the site-level reanalysis is conservative; the agreement between the two supports the robustness of our conclusions. In addition, we fitted **pooled Bayesian models** that combined temperate and alpine sites and explicitly tested a grassland-type × economic-trait interaction. This pooled interaction approach yielded the same qualitative inference for temporal stability and resistance, and the results were consistent whether implemented at the site scale or using the full dataset (**Figure S9; Table S7**).

For SEM, aggregating to the site level would yield too few observations to fit the full path model. Therefore, we did not rely on a site-only SEM. Instead, we fit SEMs on the pooled dataset combining both grassland types, and modeled ecosystem context (temperate vs. alpine) explicitly using interaction terms. We implemented this pooled SEM at the plot level and, as a sensitivity check, also at the site level using the same context-by-predictor interactions. In both cases, the pooled SEM indicated that the key paths differ by grassland type and supported conclusions consistent with the site-level re-analysis (**Fig. S10**).

Finally, to further mitigate concerns about reduced power in the site-level reanalysis, we conducted an additional robustness check **using two independent grassland survey datasets with larger numbers of sites** (temperate, n = 35; alpine, n = 33) spanning the same regions and vegetation types (**Figure S11**). We matched species composition from published sources to our standardized, field-measured trait database and reproduced community-weighted traits and stability metrics using the same workflow as in the main survey. These independent surveys confirmed the biome-dependent relationships between community economic traits and both temporal stability and resistance (**Figures S12–S13; Text S1**). These revisions are now described in the Methods and Results of the main text, with complete model details and additional robustness checks provided in the Supplementary Information.

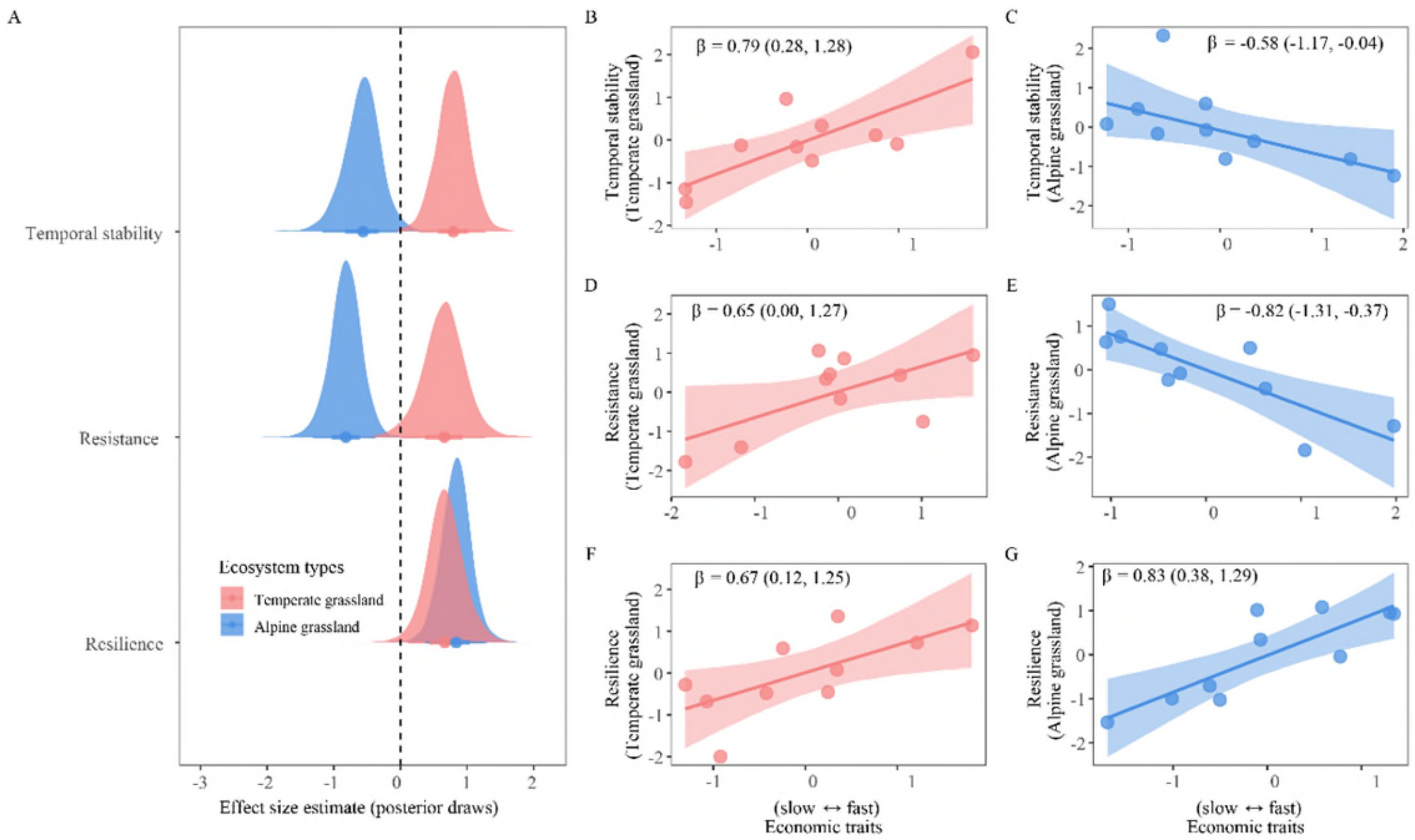

Figure S8. Effects of economic traits on ecosystem temporal stability, resistance, and resilience at the site level (n = 10 per grassland type). Panel A shows posterior distributions of standardized effect sizes; circles indicate posterior means, with 80% (thick lines) and 95% (thin lines) credible intervals from multivariate Bayesian models. Panels B–G show relationships between economic traits and temporal stability, resistance, and resilience. Each point represents a site, and each panel reports

the standardized slope (β) with its 95% credible interval from the corresponding Bayesian model. Solid lines and shaded bands indicate the posterior mean prediction and its 95% credible interval. Economic traits are summarized as PC1 from a principal component analysis of nine functional traits, where higher values indicate more acquisitive strategies and lower values indicate more conservative strategies.

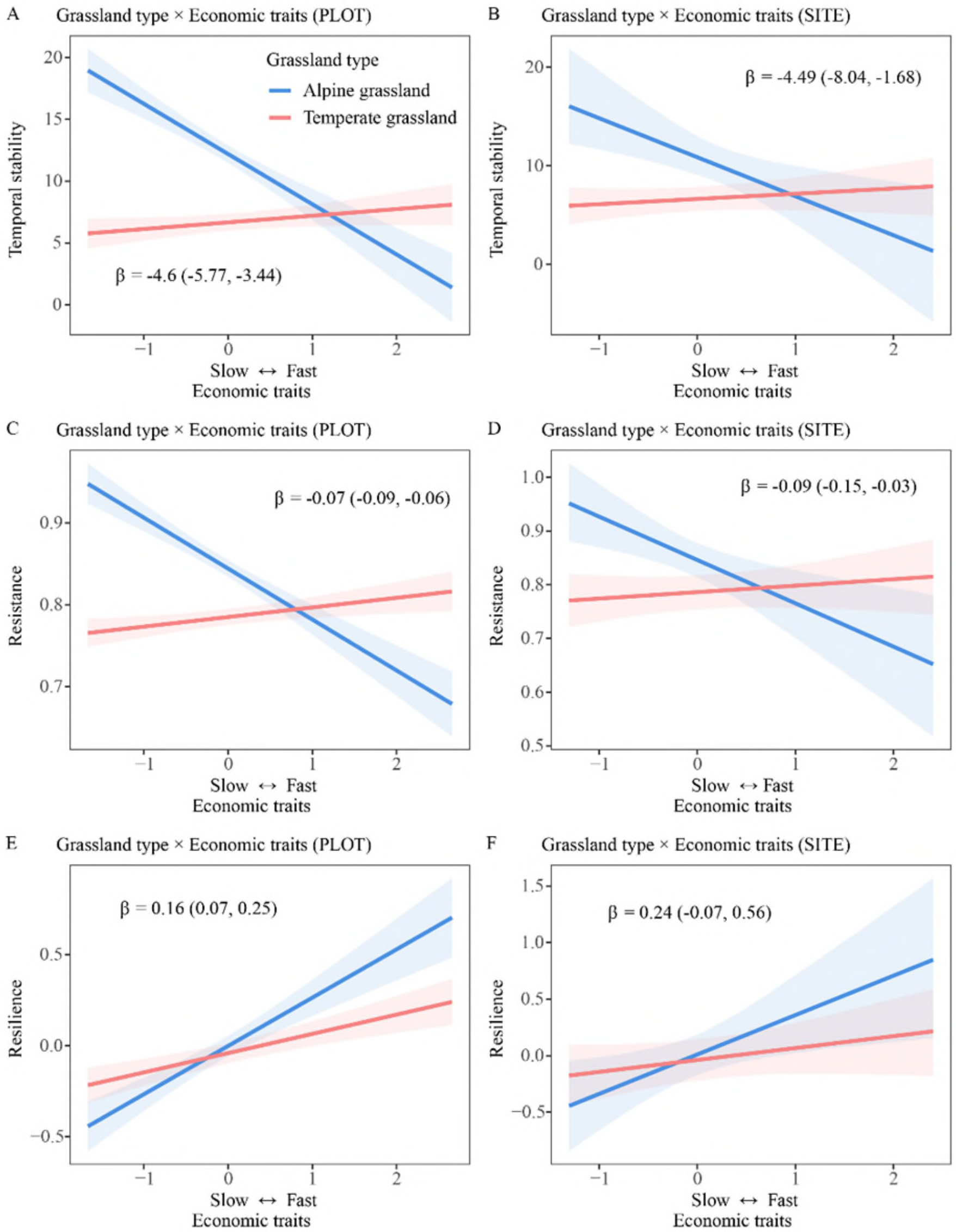

Figure S9. Main effects of economic traits, grassland type, and their interaction on components of ecosystem stability at the plot and site levels. Plot-level analyses (A, C, E) are based on 160 plots. Site-level analyses (B, D, F) are based on 20 sites. (A, C, E) Posterior estimates of standardized effect sizes for economic traits, grassland type, and their interaction (grassland type × economic traits) on temporal stability (A), resistance (C), and resilience (E), from a multivariate Bayesian model fitted to plot-level data. (B, D, F) Posterior estimates of standardized effect sizes for the same predictors on temporal stability (B), resistance (D), and resilience (F), from the corresponding multivariate Bayesian model fitted to site-level data. Line colors indicate grassland type: red = temperate grassland, blue = alpine grassland. **Across**

both levels, trait–stability relationships are context dependent, with clear interaction effects for temporal stability and resistance, whereas the interaction for resilience is weaker.

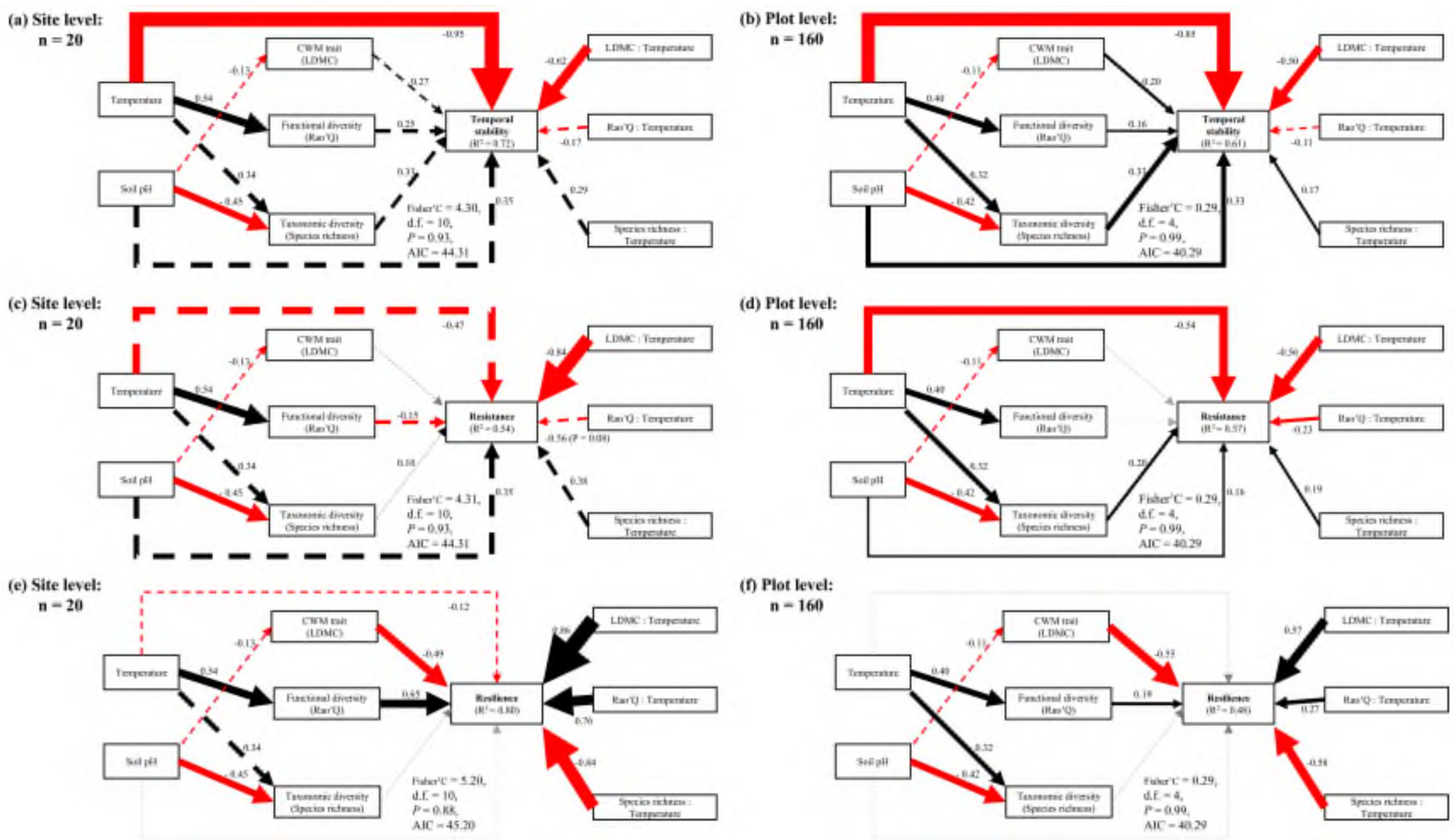

Figure S10. Bayesian structural equation models (pooled across temperate and alpine grasslands) quantifying the direct and indirect effects of climate (mean annual temperature), soil (soil pH), and an economic trait (LDMC)—including their interactions with temperature—on (A, B) temporal stability, (C, D) resistance, and (E, F) resilience. To streamline the models, we used a priori variable selection: **LDMC as the focal trait because it is widely used in trait–stability research (Májeková *et al.* 2014; Craven *et al.* 2018; Conti *et al.* 2023; Yan *et al.* 2025), and temperature as the focal climatic driver because it most strongly differentiates the two regions.** Arrow widths scale with standardized path coefficients (**β_{std}**). Black and red arrows denote positive and negative effects, respectively; solid lines indicate statistically significant paths, and dotted lines denote non-significant paths.

Supplementary Table 2. Summary table comparing abiotic and biotic factors between temperate and alpine grasslands using robust Bayesian estimation. A positive estimate indicates that the value is higher in temperate grasslands compared to alpine grasslands, whereas a negative estimate indicates higher value in alpine grasslands. Significant differences are indicated when the 95% credible interval (CI) does not include zero; otherwise, the difference is not considered significant.

Trait	Model type	N	Estimate	CI_{lower}	CI_{upper}	Significant
Species richness	Hierarchical	160	-0.2	-1.23	0.81	Not Significant
**Temperature**	**Site-level**	**20**	**-8.15**	**-9.74**	**-6.56**	**Significant**
**Precipitation**	**Site-level**	**20**	**2.14**	**-151.03**	**155.31**	**Not Significant**
**Soil properties**	**Hierarchical**	**160**	**0.33**	**-1.12**	**1.78**	**Not Significant**
**TS**	**Site-level**	**20**	**5.07**	**1.59**	**8.59**	**Significant**
**Resistance**	**Site-level**	**20**	**0.06**	**0**	**0.12**	**Significant**
**Resilience**	**Site-level**	**20**	**0.02**	**-0.3**	**0.33**	**Not Significant**
Height	Hierarchical	160	-11.21	-19.43	-2.04	Significant
LA	Hierarchical	160	-1.73	-3.67	0.21	Not Significant
SLA	Hierarchical	160	-0.88	-19.41	18.52	Not Significant
LDMC	Hierarchical	160	-3.77	-14.79	6.73	Not Significant
SDMC	Hierarchical	160	1.69	-6.56	9.92	Not Significant
RDMC	Hierarchical	160	-5.48	-14.46	3.66	Not Significant
Leaf C	Hierarchical	160	-1.12	-3.81	1.58	Not Significant
Leaf N	Hierarchical	160	-0.27	-0.94	0.44	Not Significant
Leaf P	Hierarchical	160	-0.03	-18.93	18.21	Not Significant
Root N	Hierarchical	160	-0.11	-0.37	0.16	Not Significant
Root P	Hierarchical	160	0.26	-20.07	20.19	Not Significant
SDdensity	Hierarchical	160	0.83	-17.97	19.8	Not Significant
SDlen	Hierarchical	160	-10.26	-14.71	-5.86	Significant
SDw	Hierarchical	160	-7.72	-10.12	-5.37	Significant
SDarea	Hierarchical	160	-1.93	-21.4	17.51	Not Significant
APratio	Hierarchical	160	-0.17	-0.3	-0.03	Significant
Economic traits	Hierarchical	160	-0.39	-2.5	1.81	Not Significant
Size traits pc1	Hierarchical	160	-2.49	-3.5	-1.52	Significant
Size traits pc2	Hierarchical	160	-0.53	-1.48	0.4	Not Significant

All models used a Student's t likelihood and allowed unequal variances between regions ($\sigma \sim$ Region).

Supplementary Table 5. Convergence and sampling diagnostics for plot- and site-level multivariate Bayesian models quantifying relationships between three stability components (temporal stability, resistance, and resilience) and economic traits. We report standardized posterior mean effects of the economic trait predictor (β; mean [95% credible interval]) for each Scale (Plot vs Site) × Region × stability component combination, together with Markov chain convergence and sampling efficiency metrics (Rhat, Bulk_ESS, Tail_ESS). Effect estimates were quantitatively consistent between plot- and site-level analyses; however, credible intervals were generally wider at the site level due to the smaller sample size (n = 10 per grassland type).

Level	Region	Response	Est_ci	Rhat	Bulk_ESS	Tail_ESS
Plot	Temperate grassland	Temporal stability	0.65 [0.50, 0.79]	1.00	4313	3455
Plot	Temperate grassland	Resistance	0.33 [0.16, 0.51]	1.00	4004	4015
Plot	Temperate grassland	Resilience	0.55 [0.39, 0.70]	1.00	5393	4462
Plot	Alpine grassland	Temporal stability	-0.57 [-0.73, -0.43]	1.00	5157	4724
Plot	Alpine grassland	Resistance	-0.70 [-0.83, -0.56]	1.00	4583	4133
Plot	Alpine grassland	Resilience	0.59 [0.44, 0.74]	1.00	5367	4490
Site	Temperate grassland	Temporal stability	0.79 [0.28, 1.28]	1.00	4483	3564
Site	Temperate grassland	Resistance	0.65 [0, 1.27]	1.00	4957	3635
Site	Temperate grassland	Resilience	0.67 [0.12, 1.25]	1.00	4838	3562
Site	Alpine grassland	Temporal stability	-0.58 [-1.17, -0.04]	1.00	3001	2321
Site	Alpine grassland	Resistance	-0.82 [-1.31, -0.37]	1.00	4656	3344
Site	Alpine grassland	Resilience	0.83 [0.38, 1.29]	1.00	3887	3355

Note: ***β*** values are standardized posterior means with 95% credible intervals (CrI). All rows report the effect of the economic trait axis. Rhat values close to 1.00 and large effective sample sizes (Bulk_ESS and Tail_ESS) indicate satisfactory convergence and mixing. Plot-level models used a Gaussian likelihood, whereas site-level models used a Student's t likelihood to improve robustness under small sample size. Asterisks denote effects whose 95% credible intervals exclude zero. Higher values of the economic trait axis indicate more acquisitive (fast) strategies (lower values indicate more conservative strategies).

Supplemental Table 7. Posterior summaries for the grassland type × economic traits interaction term from multivariate Bayesian models at plot and site levels. The table reports the estimated interaction effects on temporal stability, resistance, and resilience, including posterior mean (Estimate), posterior SD (Est.Error), 95% credible intervals (l-95% CI, u-95% CI), and convergence/ESS diagnostics (Rhat, Bulk_ESS, Tail_ESS).

Model	Term	Estimate	l-95% CI	u-95% CI	Rhat	Bulk_ESS	Tail_ESS
Plot	Temporal stability	-4.6	-5.77	-3.44	1.00	3451	3968
Plot	Resistance	-0.07	-0.09	-0.06	1.00	3020	4250
Plot	Resilience	0.16	0.07	0.25	1.00	3486	4190
Site	Temporal stability	-4.49	-8.04	-1.68	1.00	4917	3821
Site	Resistance	-0.09	-0.15	-0.03	1.00	6210	3848
Site	Resilience	0.24	-0.07	0.56	1.00	6040	4346

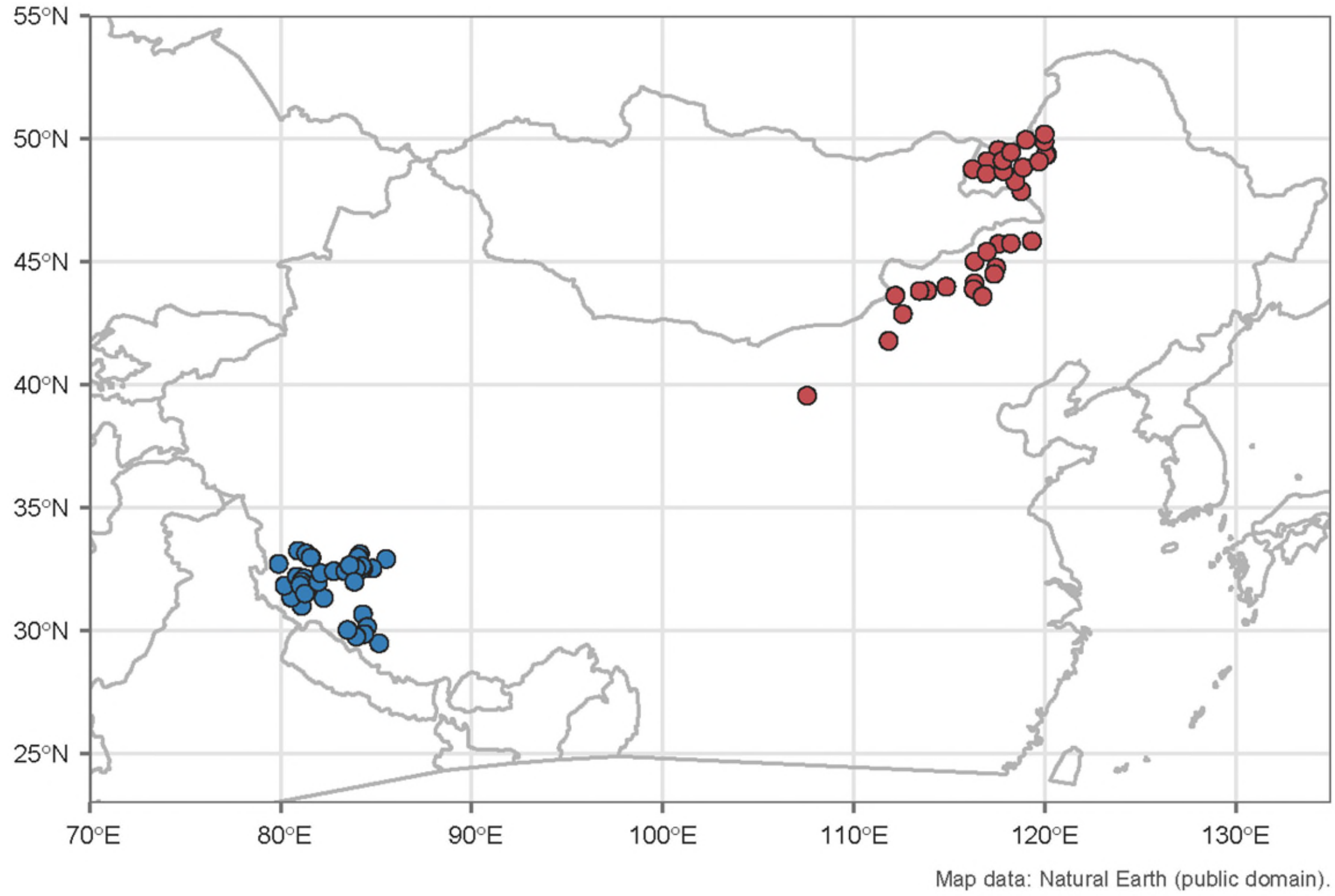

Figure S11 Geographic distribution of independent transect sites used for external validation. Blue circles indicate alpine grassland sites on the Tibetan Plateau (n = 33), and red circles indicate temperate grassland sites on the Mongolian Plateau (n = 35). The map was created by the authors in R using the sf and ggplot2 packages. Country boundaries and coastlines were obtained from Natural Earth (public domain; https://www.naturalearthdata.com/about/terms-of-use/) through the rnaturalearth package.

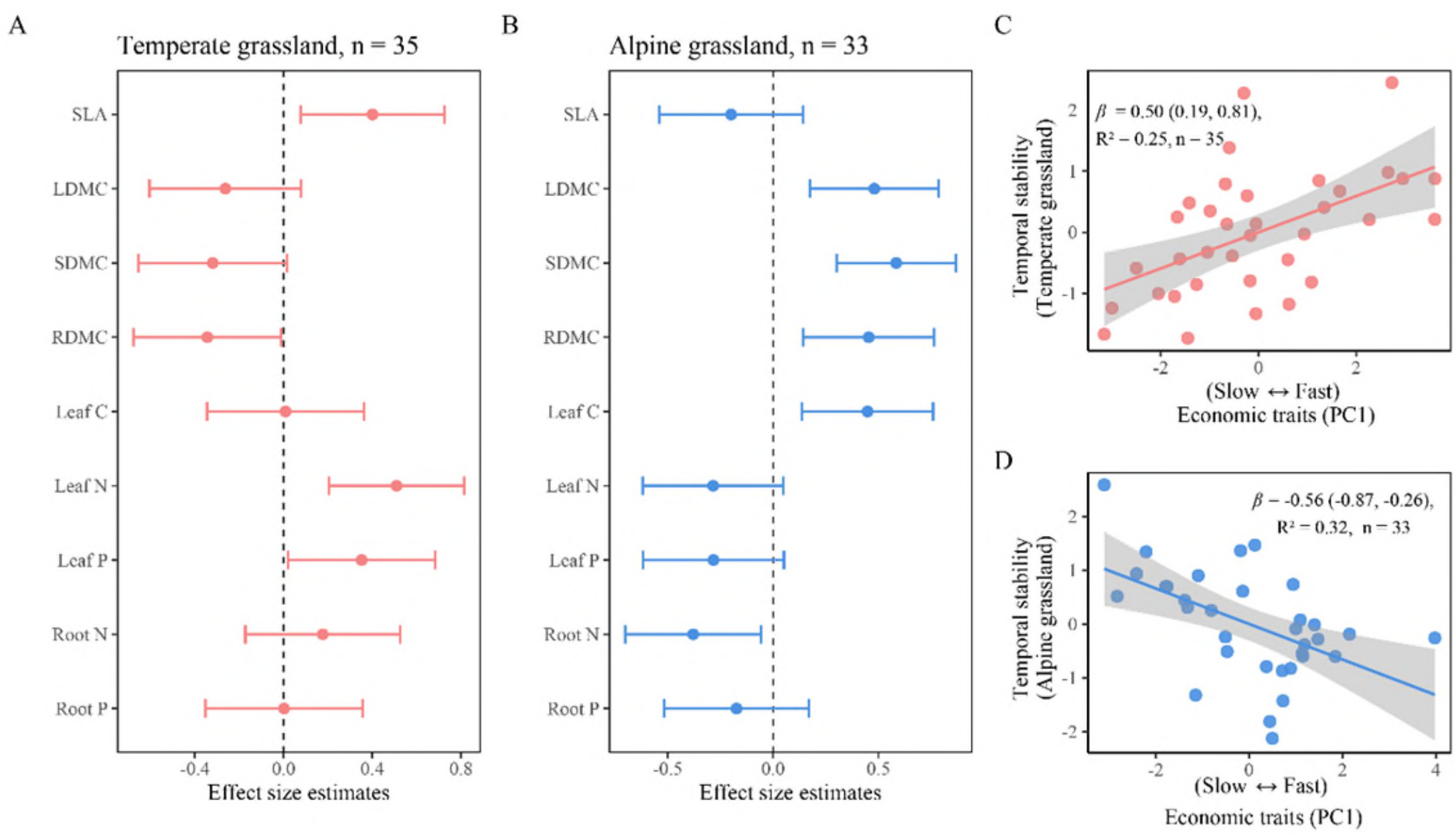

Figure S12 Independent-dataset validation: effects of plant economic traits on ecosystem temporal stability (temperate transect: n = 35; alpine transect: n = 33). Panels A–B display standardized effect sizes (posterior means ± 95% credible intervals) from biome-specific models. Panels C–D show the relationships between economic traits and temporal stability; solid lines indicate mean model predictions and shaded bands are 95% credible intervals. Economic traits are summarized as the first principal component (PC1) derived from a principal component analysis of the economic-trait subset identified in the main analysis.

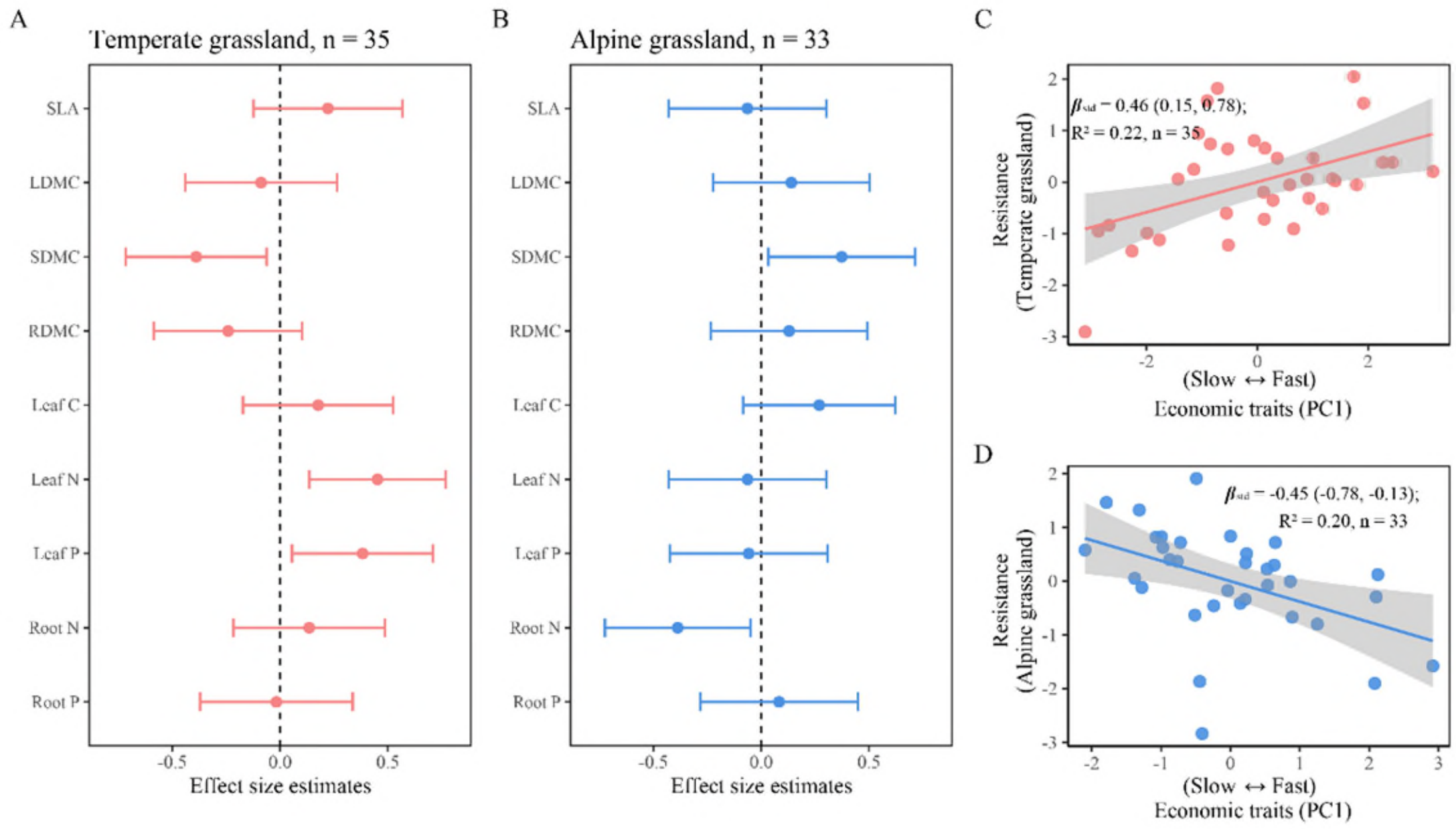

Figure S13 Independent-dataset validation: effects of plant economic traits on ecosystem resistance (temperate transect: n = 35; alpine transect: n = 33). Panels A–B display standardized effect sizes (posterior means ± 95% credible intervals) from biome-specific models. Panels C–D show the relationships between economic traits and resistance; solid lines indicate mean model predictions and shaded bands are 95% credible intervals. Economic traits are summarized as the first principal component (PC1) derived from a principal component analysis of the economic-trait subset identified in the main analysis.

- *Line 339-344: Is the PCA performed on plots or species? If on plots, did the authors account for site-level dependence? Clarify and justify and/or rectify.*

Response: The PCA was conducted at the plot level (n = 160 plant communities) to summarize multivariate trait variation among communities. Because multiple plots were sampled within each site, plots are not fully independent. To address this potential site-level dependence, we conducted two complementary robustness checks. First, we repeated the PCA after aggregating trait values to the site level (i.e., averaging across plots within each site) and observed the same ordination pattern (**Fig. S15**). Second, we tested for regional differences in functional composition using PERMANOVA on standardized trait distances at the site level, which confirmed a

significant separation between temperate and alpine grasslands ($R^2 = 0.19$, F = 4.22, $P = 0.0035$; **Table S5**). In addition, at the plot level we verified that the inference is unchanged when using a permutation scheme that accounts for site clustering ($R^2 = 0.156$, F = 29.20, $P = 0.003$; **Table S5**). Together, these analyses indicate that our conclusions regarding regional differences in trait composition are robust to non-independence among plots. We have revised the Methods and Results accordingly and added the site-level PCA and PERMANOVA results to the Supplementary Information.

For example, see the revised **Lines 470–478**:

"Because multiple plots were sampled within sites, we assessed the robustness of regional differences in functional composition to potential site-level non-independence in two complementary ways. First, we repeated the PCA after aggregating trait values to the site level (i.e., averaging across plots within each site), which yielded the same ordination pattern (Figure S3). Second, we tested for regional differences in functional composition using PERMANOVA (vegan, v2.7.2) on standardized trait distances, implementing a plot-level test with permutations constrained by site (site-clustered permutations) and a site-level test based on site-mean trait values (Table S3)."

Table S5 Regional differences in multivariate trait composition between temperate and alpine grasslands assessed by PERMANOVA under alternative permutation schemes.

**Method**	**n_plots**	**n_sites**	**n_perm**	**F_obs**	**R^2**	**P-value**
PERMANOVA (plot level; free permutations)	160	20	9999	29.18	0.160	0.0001
PERMANOVA (plot level; clustered permutations by site)	160	20	9999	29.20	0.156	0.003
PERMANOVA (site level; free permutations)	NA	20	9999	4.22	0.189	0.0035

PERMANOVA was performed on Euclidean distances calculated from z-standardized trait matrices. To account for potential non-independence among plots sampled within the same site, we report a plot-level test using site-clustered (restricted) permutations as well as a site-level test based on site-mean trait values (see Methods; Fig. SX). Values shown are the observed pseudo-F statistic (F_obs), variance explained (R^2), and permutation ***P***-values (***P***_value; 9,999 permutations). method (PERMANOVA scheme), n_plots (number of plots), n_sites (number of sites), n_perm (number of permutations), F_obs (observed pseudo-F), R^2_obs (variance explained), ***P***_value (permutation ***P***-value).

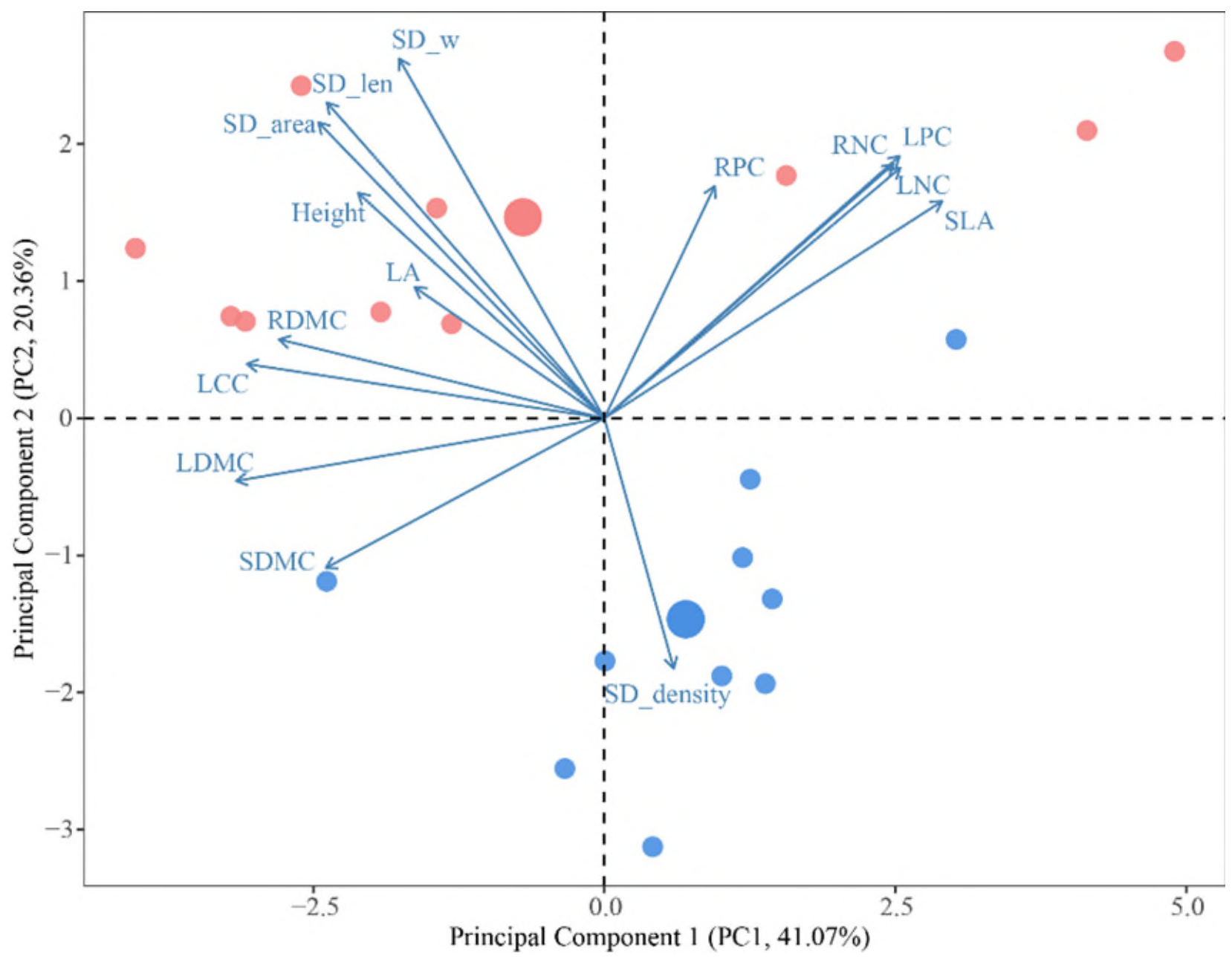

Figure S3 Site-level trait space captured by PCA (n = 20 sites). Principal component analysis (PCA) was performed on community-weighted mean values of 16 plant functional traits, aggregated to the site level by averaging across plots within each site (temperate and alpine grasslands combined). Traits included leaf carbon (Leaf C, %), leaf nitrogen (Leaf N, %), leaf phosphorus (Leaf P, mg g^{-1}), leaf area (LA, cm^2), leaf dry matter content (LDMC, %), plant height (Height, cm), specific leaf area (SLA, cm^2 g^{-1}), stem dry matter content (SDMC, %), root nitrogen (Root N, %), root phosphorus (Root P, mg g^{-1}), root dry matter content (RDMC, %), and stomatal traits (density, length, width, and area). All traits were standardized (z-score; mean = 0, SD = 1) prior to PCA. Points represent sites and are colored by region; arrows indicate trait loadings. **PC1 captures the plant economic spectrum (higher values indicate more acquisitive strategies, with SLA loading positively), whereas PC2 reflects an orthogonal axis of trait variation primarily associated with plant size-related attributes**.

- *Lines 345-392:* ***Bayesian linear and structural equation models apparently lack hierarchical structure.*** *The models do not appear to account for the sampling design or plot dependence within sites. Models must include random effects for sites. The manuscript should provide: (1) complete model formula in R syntax or mathematical notation showing hierarchical structure, (2) explicit prior specifications for all parameters with justification, (3) model family with reasoning, (4) convergence diagnostics, and (5) brms package version.*

Response: We fully agree with the reviewer that our Bayesian linear models and SEMs should explicitly reflect the nested sampling design (plots within sites). We have therefore re-run the analyses, revised the Methods and Results, and added additional robustness checks (including validation using an independent grassland survey dataset) to ensure that our conclusions are not artifacts of model structure. These revisions are now described in the main text, with complete model syntax and

supporting outputs provided in the Supplementary Information.

(1) Hierarchical structure and sampling design (plots nested within sites)
Where a plot-level response exhibits within-site variation, our updated models now include a site-level random intercept to account for non-independence among plots from the same site (e.g., (1 | Site_id)).
Importantly, our EVI-based stability metrics (temporal stability, resistance, and resilience) are derived at the site level from a common remote-sensing time series, such that all plots within a site share the same underlying stability signal. We therefore re-analyzed these trait–stability relationships at the site level. Results from the site-level analyses were consistent with plot-level patterns (albeit with reduced power due to smaller n), supporting the robustness of our inference (e.g., Figures S8–S10; Table S5). We further corroborated these conclusions using two independent grassland survey datasets spanning the same regions and vegetation types (Figures S11–S13).

(2) Complete model formulas (including multivariate models and SEM)
We now provide complete model specifications in R syntax in the Methods/Supplement. For example, the multivariate Bayesian model for the three stability dimensions is reported as:

Plot-level models (when responses vary within site): we used two complementary Bayesian modeling frameworks depending on the inferential goal. ***(i)*** Regional contrasts. To quantify mean differences between regions while accommodating unequal residual variances, we fitted robust distributional models with a site-level random intercept and region-specific residual scale, e.g., **bf(y ~ Region + (1|Site_id)**, **sigma ~ Region**) (and analogously for other responses). Where posterior predictive checks suggested heavy tails or outlier sensitivity, we adopted a robust likelihood (Student’s t) under the same hierarchical and heteroscedastic specification. ***(ii)*** Regression-type hierarchical models. To evaluate continuous trait–environment relationships at the plot level while accounting for the sampling design, we fitted Bayesian mixed-effects (nested/hierarchical) regression models using a Gaussian likelihood, with site as a random intercept, e.g., **bf(y ~ x + covariates + (1|Site_id)) (with family = gaussian())**, and included additional predictors/interactions as needed.

Site-level models (when responses are site-derived): we fitted Bayesian Gaussian regression models of the form **bf(y_site ~ predictors_site, ...)** without a site random effect because each site contributes a single observation. Given the smaller site-level sample size, we used parsimonious model specifications (and weakly informative priors where needed) to improve computational stability and ensure reliable convergence.

(3) Priors (all parameters) and justification
We now state priors for all parameter classes. Fixed effects use weakly informative

Normal(0, 10) priors (consistent with recent trait-based Bayesian analyses (Umaña *et al.* 2023)), with predictors and responses standardized to improve interpretability and prior calibration. Residual and random-effect standard deviations use Exponential(1) priors, and correlation structures (when estimated) use LKJ(2) priors. For Student's t models, we additionally specify a weakly informative prior on the degrees of freedom ν. These priors provide mild regularization without unduly constraining effect sizes.

(4) Likelihood family and rationale
We now report and justify likelihood choices explicitly. Plot-level models were fit with a Gaussian likelihood when appropriate. For site-level models with smaller sample size (n = 10 per grassland type), we used a Student's t likelihood to improve robustness to potential outliers and heavy-tailed residuals; when heavy tails are weak, the Student's t model approaches a Gaussian error structure.

(5) Convergence diagnostics, model checking, and software version
All models were fitted in **brms** v2.23.0 (full session information is now provided). We assessed convergence and sampling adequacy using $\hat{R}$ (≤ 1.01), **effective sample sizes** (Bulk_ESS and Tail_ESS), and visual inspection of **trace plots and density overlays** (e.g., **Figs. R1–R8**). When occasional divergent transitions occurred, we increased adapt_delta and, when necessary, max_treedepth to improve sampler behavior. Model fit was evaluated using posterior predictive checks (e.g., pp_check). These procedures and key diagnostic summaries are now explicitly described in the Methods and Supplement; for example, the relevant modeling details are provided in the Methods (**lines 505–530**).

"Each Bayesian model included four chains, each with 3,000 iterations, including 1, 500 warm-up iterations. We used weakly informative priors for all parameters. Specifically, we assigned Normal(0,10) priors to population-level effects (intercepts and slopes) after standardizing predictors and responses, consistent with recommended practice for weakly informative priors in ecological Bayesian analyses and recent trait-based Bayesian studies [29,62]. Standard-deviation parameters (residual SD and, where applicable, random-effect SDs) were assigned Exponential(1) priors. When correlation structures were estimated (e.g., residual or group-level correlations), we assigned LKJ(2) priors. For Student's t models, we additionally specified a weakly informative prior on the degrees of freedom (ν). When diagnostic warnings indicated sampling problems (e.g., divergent transitions), we refitted the affected models using a higher target acceptance rate and allowing deeper NUTS trajectories (i.e., control = list(adapt_delta = 0.95, max_treedepth = 12)). This adjustment improves numerical stability of Hamiltonian Monte Carlo in the presence of challenging posterior geometries (e.g., strong parameter correlations or funnel-shaped regions), thereby reducing divergent transitions and yielding more reliable posterior inference. Convergence and chain mixing were assessed visually using the bayesplot package (v1.15.0) [63] and numerically with the Gelman-Rubin statistic ($\hat{R}$ < 1.01) [64]. Effective sample sizes exceeded 10% of post-warm-up iterations. Posterior means, and 80%

and 95% Bayesian CIs, were displayed using raincloud plots (implemented with tidybayes v3.0.7 and ggplot2 v4.0.1) for all fixed model parameters. Conditional effects plots, generated via the conditional_effects function in the brms package (v2.23.0), visualized the relationships between explanatory and response variables. Model fit was evaluated using posterior predictive checks and R^2 from the bayes_R^2 function in brms (v2.23.0) [65]. Parameters were considered significant if their 95% CIs did not overlap with zero."

In summary, we revised the manuscript to (***i***) explicitly model the nested design with site random effects whenever plot-level dependence exists, and (***ii***) conduct site-level analyses when responses are site-derived. All revisions are described in the main text, with complete model syntax and additional results provided in the Supplementary Information.

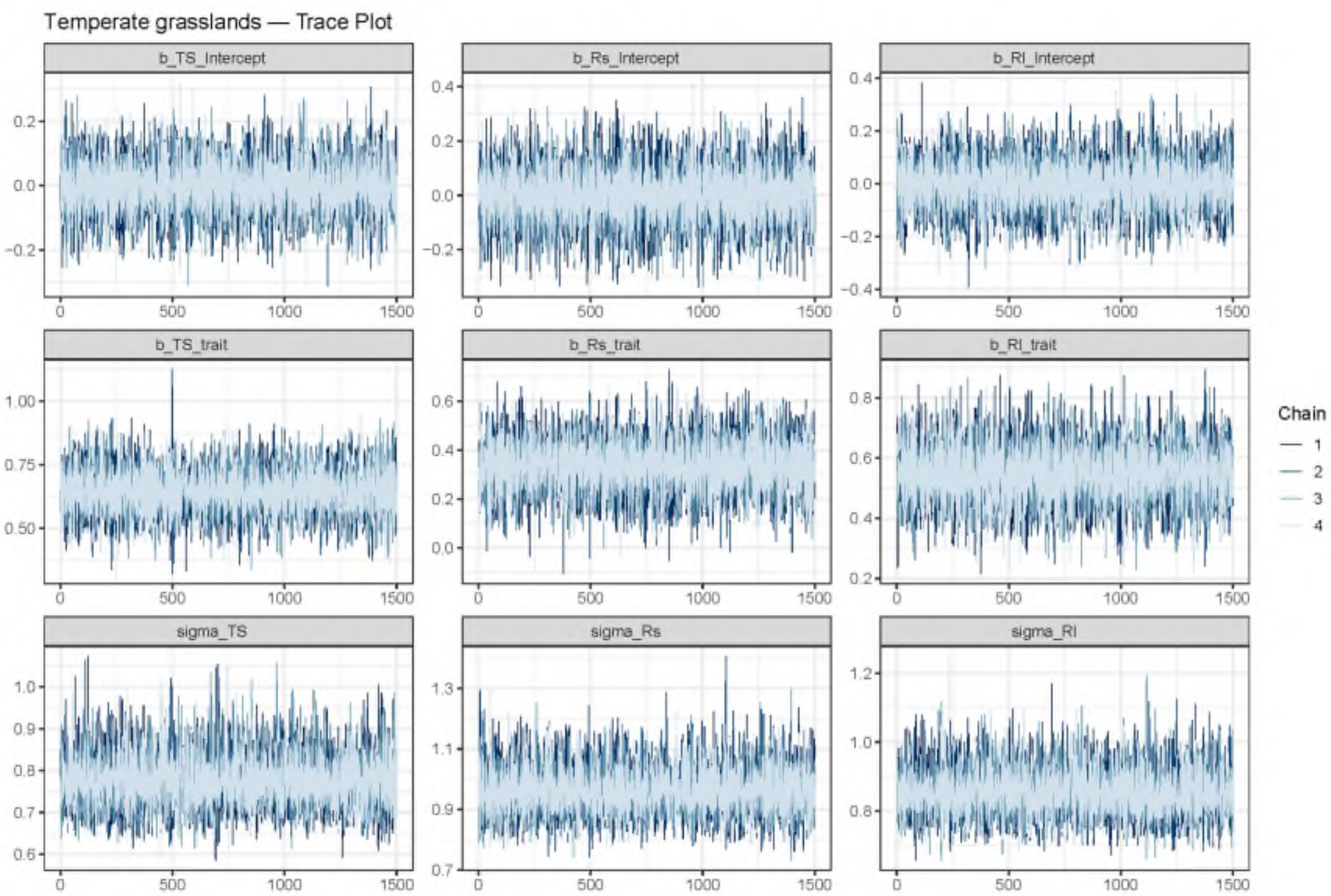

Figure R1. Trace plots for the plot-level Bayesian models relating the economic trait axis to stability components in temperate grasslands (n = 80). Trace plots show post-warmup MCMC draws from four chains for key parameters in the plot-level multivariate Gaussian model fitted to the temperate grassland dataset (n = 80 plots). The model jointly analyzes three standardized stability responses—temporal stability (TS), resistance (Rs), and resilience (Rl)—each regressed on the economic trait axis. Parameters shown include response-specific intercepts and slopes, as well as residual standard deviations (sigma). **Good chain mixing and stable stationarity indicate satisfactory convergence.**

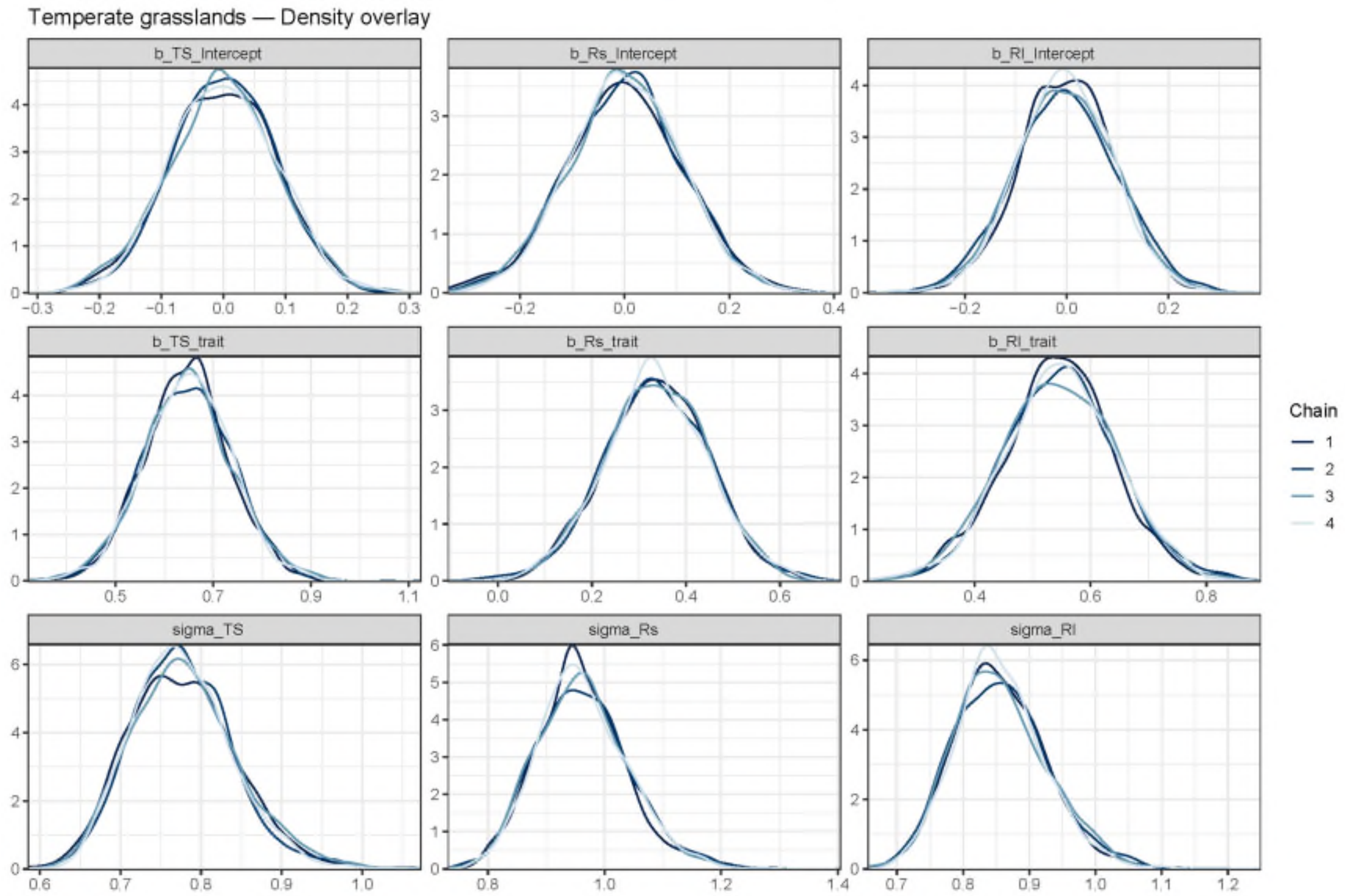

Figure R2. Density overlay plots for the plot-level Bayesian models relating the economic trait axis to stability components in temperate grasslands (n = 80). Density overlays show the marginal posterior distributions for key parameters from the plot-level multivariate Gaussian model fitted to the temperate grassland dataset (n = 80 plots), with curves colored by chain (four chains). Parameters shown include response-specific intercepts and slopes for temporal stability (TS), resistance (Rs), and resilience (Rl), as well as residual standard deviations (sigma). **Strong overlap among chains indicates consistent exploration of the posterior and supports satisfactory convergence.**

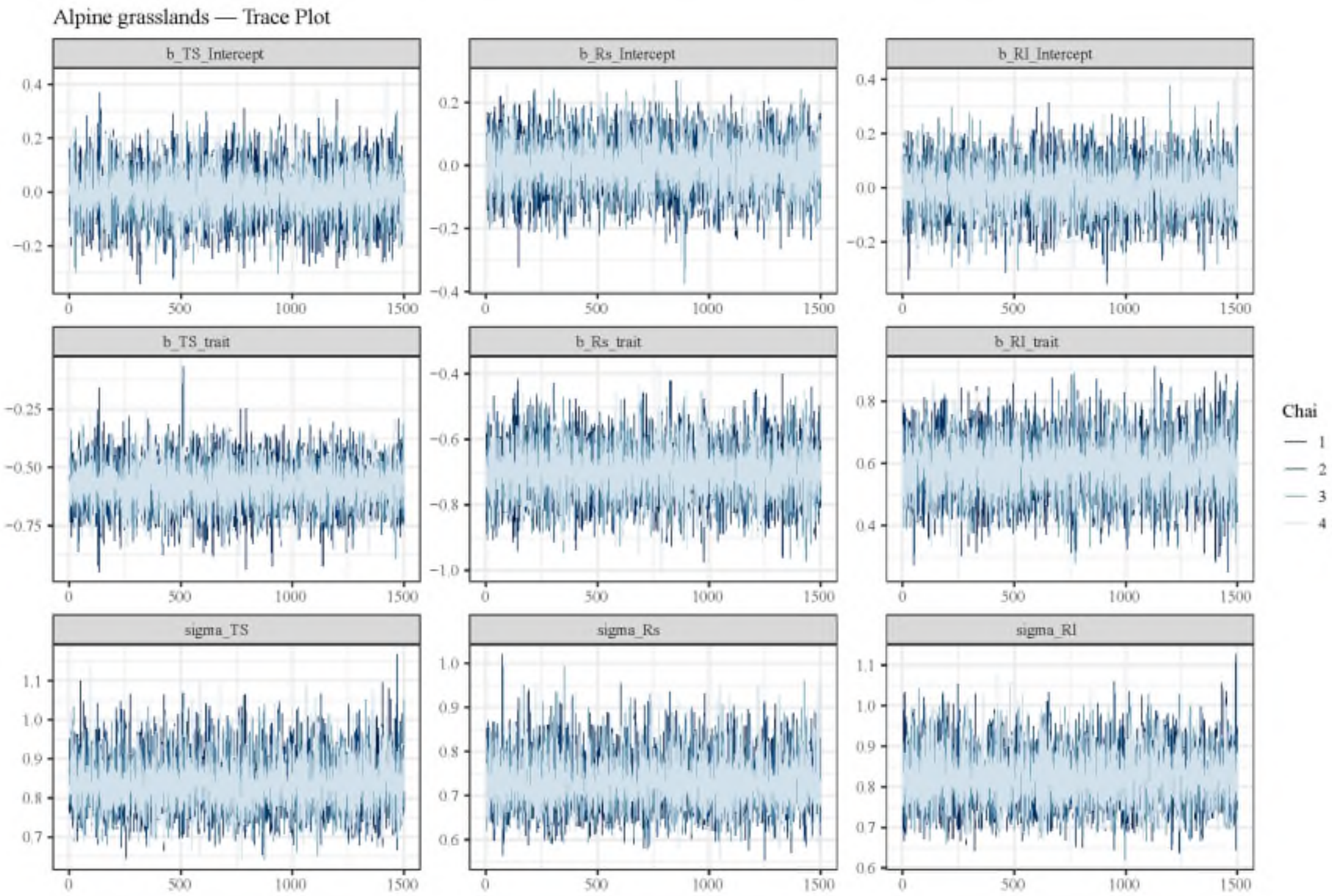

Figure R3. Trace plots for the plot-level Bayesian models relating the economic trait axis to stability components in alpine grasslands (n = 80). Trace plots show post-warmup MCMC draws from four chains for key parameters in the plot-level multivariate Gaussian model fitted to the alpine grassland dataset (n = 80 plots). The model jointly analyzes three standardized stability responses—temporal stability (TS), resistance (Rs), and resilience (Rl)—each regressed on the economic trait axis. Parameters shown include response-specific intercepts and slopes, as well as residual standard deviations (sigma). **Good chain mixing and stable stationarity indicate satisfactory convergence.**

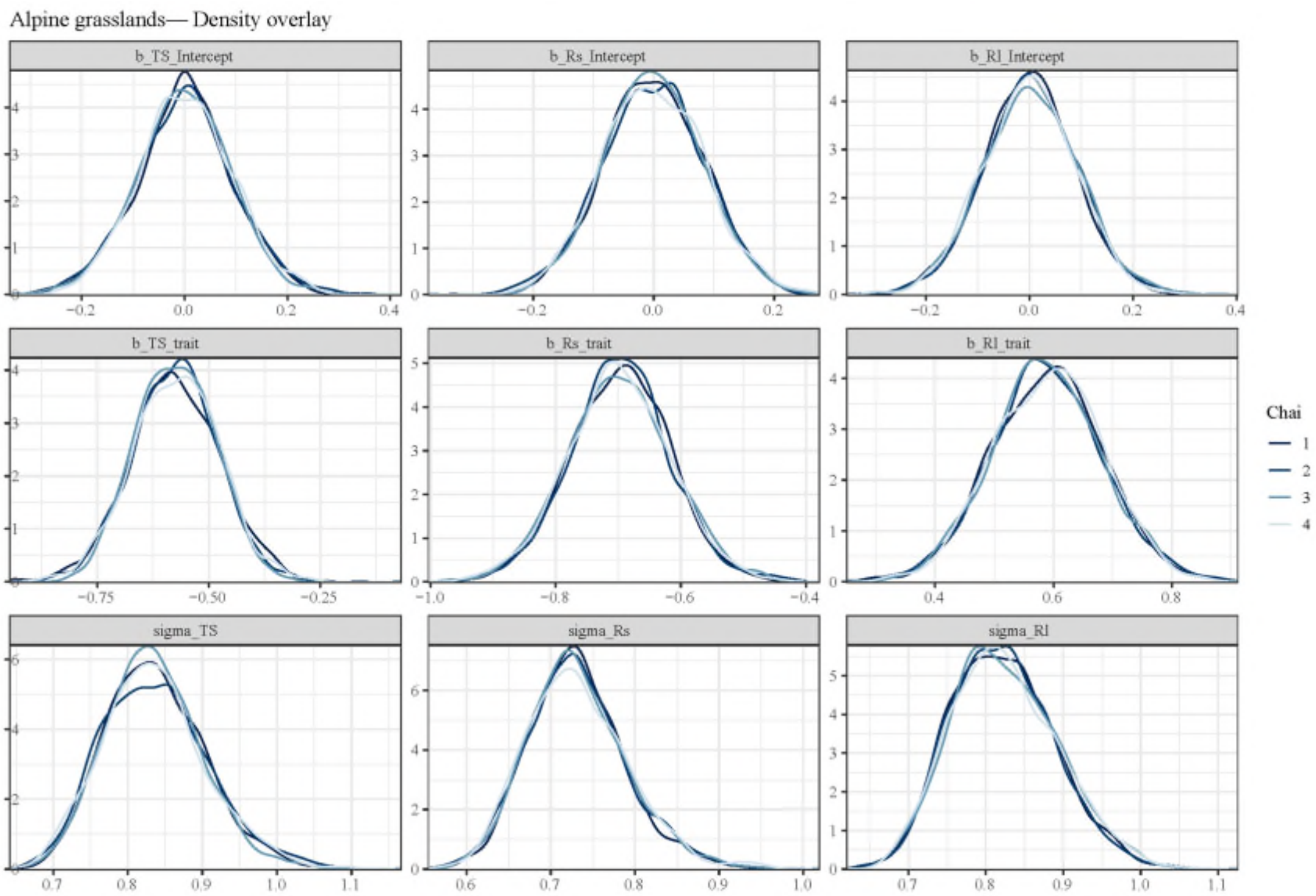

Figure R4. Density overlay plots for the plot-level Bayesian models relating the economic trait axis to stability components in alpine grasslands (n = 80). Density overlays show the marginal posterior distributions for key parameters from the plot-level multivariate Gaussian model fitted to the alpine grassland dataset (n = 80 plots), with curves colored by chain (four chains). Parameters shown include response-specific intercepts and slopes for TS, Rs, and Rl, as well as residual standard deviations (sigma). **Strong overlap among chains indicates consistent posterior sampling and supports satisfactory convergence.**

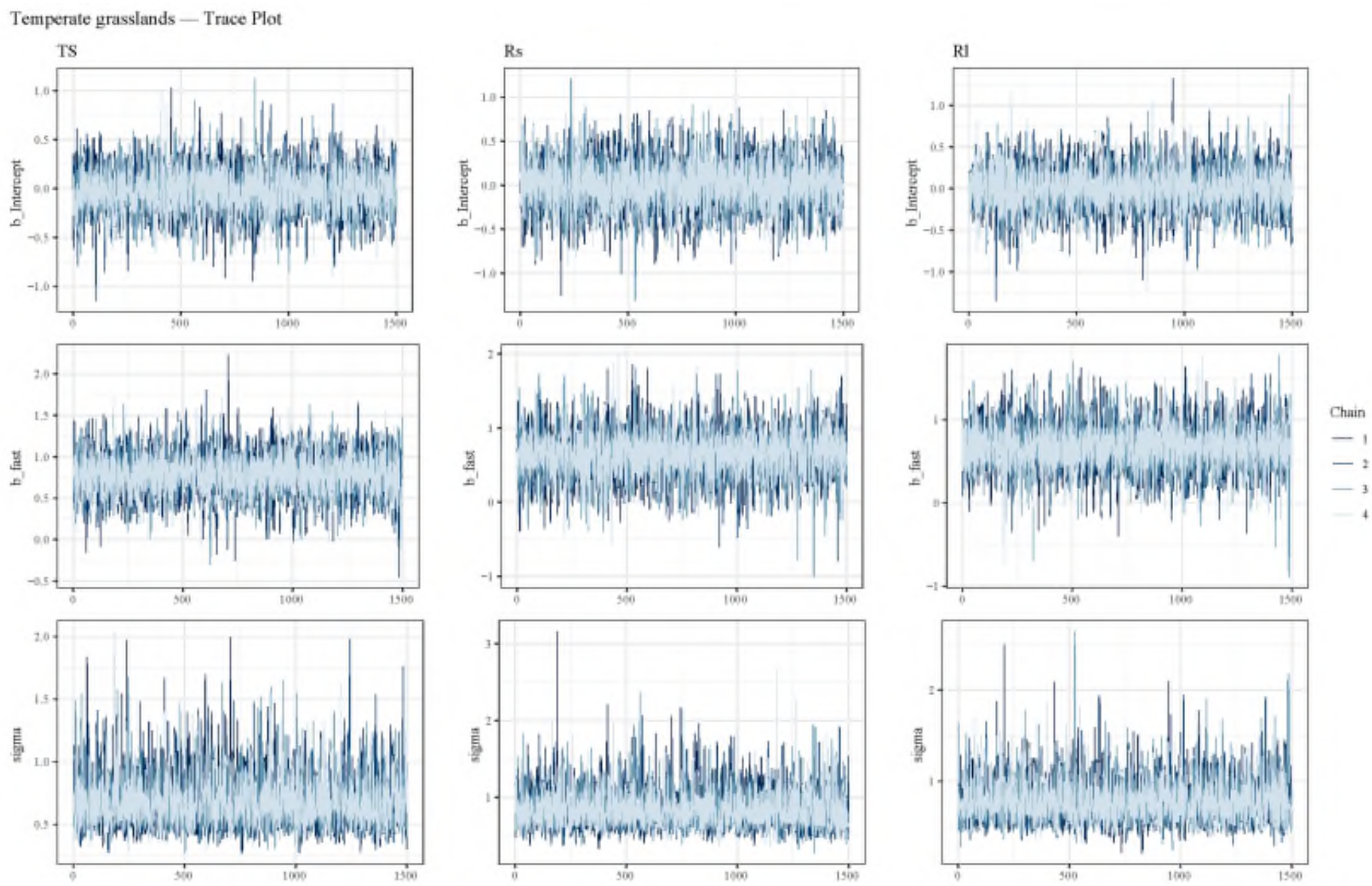

Figure R5. Trace plots for the site-level Bayesian models relating the economic trait axis to stability components in temperate grasslands (n = 10). Trace plots show post-warmup MCMC draws from four chains for key parameters in the site-level Student's t regression fitted to the temperate grassland dataset (n = 10 sites). The standardized stability response (TS, Rs, or Rl; shown in separate panels) is regressed on the economic trait axis. Parameters shown include the intercept, the trait slope, and distributional parameters (sigma). **Good chain mixing and stable stationarity indicate satisfactory convergence.**

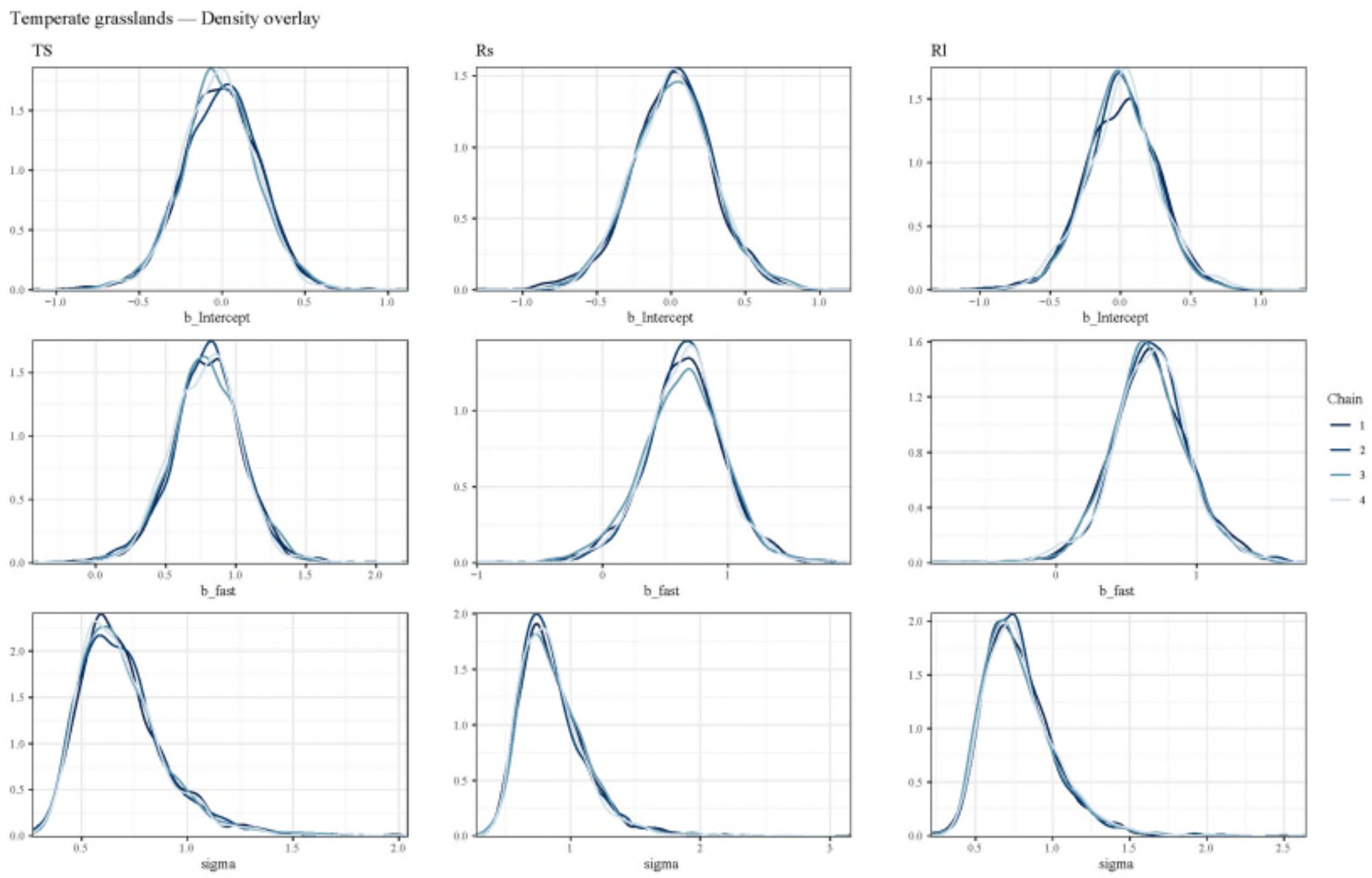

Figure R6. Density overlay plots for the site-level Bayesian models relating the economic trait axis to stability components in temperate grasslands (n = 10). Density overlays show the marginal posterior distributions for key parameters from the site-level Student's t regressions fitted to the temperate grassland dataset (n = 10 sites), with curves colored by chain (four chains). Separate panels correspond to TS, Rs, and Rl models. Parameters shown include the intercept, the trait slope, and distributional parameters (sigma). **Strong overlap among chains indicates good mixing and supports satisfactory convergence**.

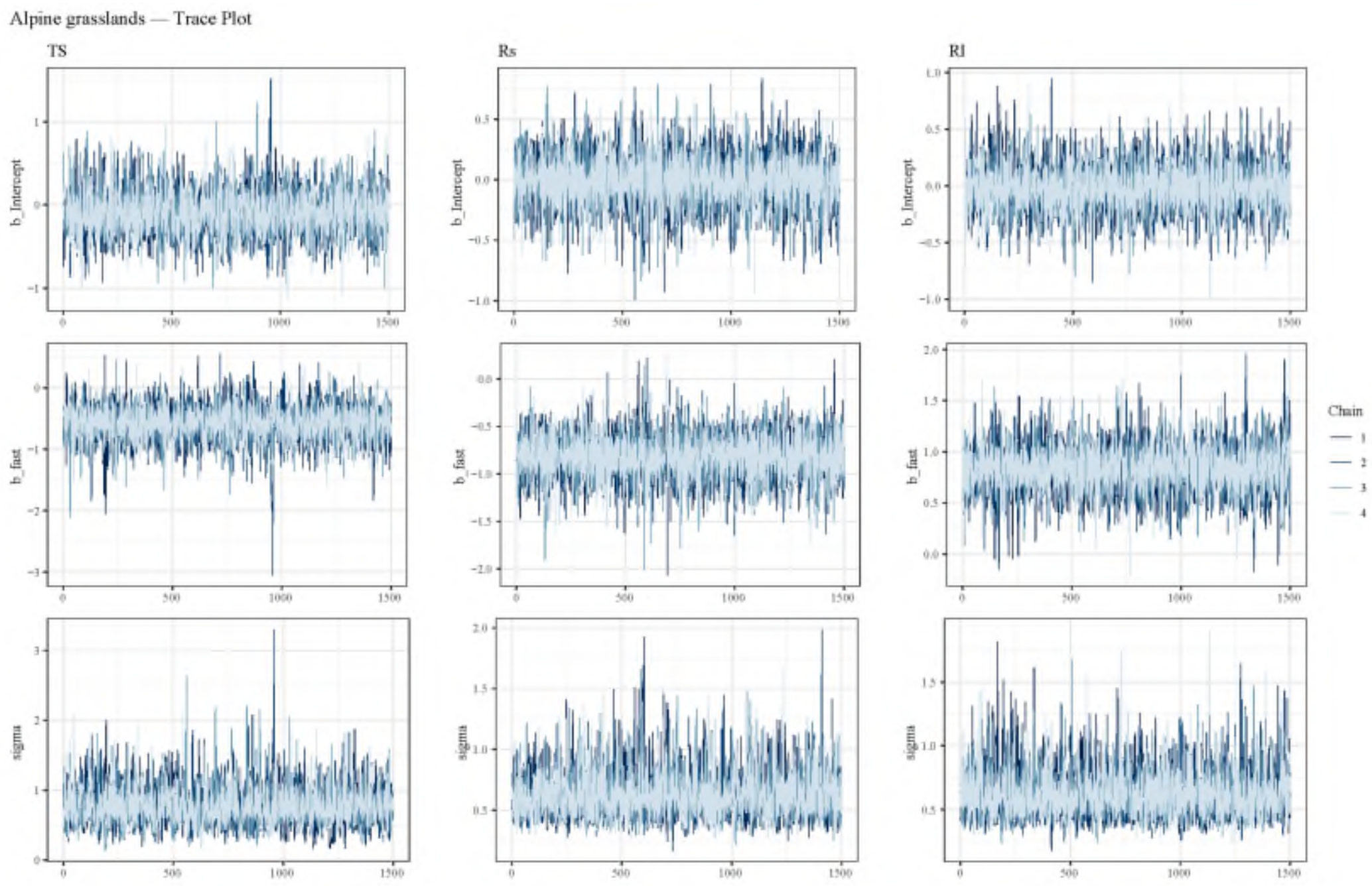

Figure R7. Trace plots for the site-level Bayesian models relating the economic trait axis to stability components in alpine grasslands (n = 10). Trace plots show post-warmup MCMC draws from four chains for key parameters in the site-level Student's t regression fitted to the alpine grassland dataset (n = 10 sites). The standardized stability response (TS, Rs, or Rl; shown in separate panels) is regressed on the economic trait axis. Parameters shown include the intercept, the trait slope, and distributional parameters (sigma). **Good chain mixing and stable stationarity indicate satisfactory convergence**.

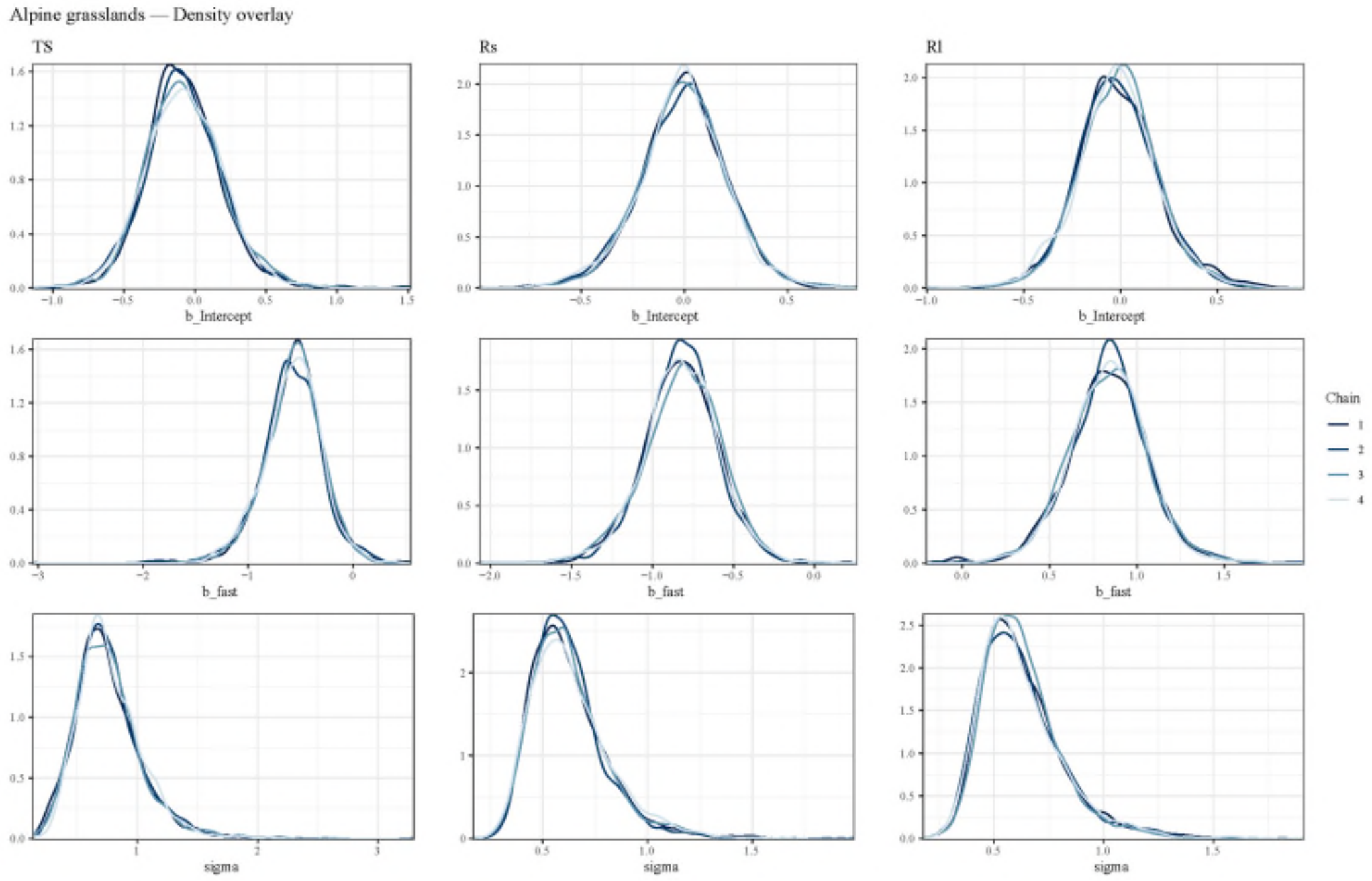

Figure R8. Density overlay plots for the site-level Bayesian models relating the economic trait axis to stability components in alpine grasslands (n = 10). Density overlays show the marginal posterior distributions for key parameters from the site-level Student's t regressions fitted to the alpine grassland dataset (n = 10 sites), with curves colored by chain (four chains). Separate panels correspond to TS, Rs, and Rl models. Parameters shown include the intercept, the trait slope, and distributional parameters (sigma). **Strong overlap among chains indicates good mixing and supports satisfactory convergence**.

- *Lines 303-321: Insufficient justification for wet/dry thresholds. Why were SPI wet/dry thresholds specifically chosen? Why not temperature extremes? Provide clear ecological justification for threshold selection appropriate to these grassland ecosystems. Furthermore, citations for resistance and resilience indices are from soil biota papers (line 321).* ***Explain why these methods are appropriate for plant communities or cite relevant plant ecology references****.*

Response: We appreciate the reviewer's request for clearer justification of (i) the wet/dry thresholds used to classify hydroclimatic extremes and (ii) the resistance/resilience indices applied to vegetation productivity. We have revised the Methods and Supplementary Text S5 to clarify the ecological rationale, the interpretation of the thresholds, and the applicability of the metrics to plant communities and EVI-based productivity.

(1) Why SPEI (rather than temperature extremes alone): We used SPEI because it characterizes **water-balance anomalies** by integrating precipitation supply with **temperature-driven atmospheric evaporative demand**, making it well suited for identifying drought/wetness conditions relevant to grassland productivity. In these

(often water-limited) grassland ecosystems, interannual variability in greenness and productivity is primarily constrained by moisture balance; therefore, SPEI provides a more mechanistically appropriate index of climatic stress than temperature extremes alone. We now state this rationale explicitly in the Methods (revised **lines 385–388**).

"We used the SPEI to classify growing-season hydroclimatic extremes because it integrates precipitation anomalies with temperature-driven evaporative demand, thereby capturing water-balance stress that is mechanistically linked to grassland productivity."

(2) Why ±0.67 as wet/dry thresholds: We classified growing seasons as dry/wet using thresholds of ±0.67 (normal: −0.67 < SPEI < 0.67; dry: SPEI ⩽ −0.67; wet: SPEI ⩾ 0.67). Because SPEI is standardized relative to each site's local baseline, these cutoffs have a clear probabilistic interpretation (moderate anomalies that occur with sufficient frequency) and provide a pragmatic balance between ecological relevance and adequate event counts for estimating site-level resistance and resilience. We clarify this interpretation and rationale in the Methods and Text S5 (**revised lines 388–392**).

"We adopted ±0.67 because it represents moderate moisture anomalies (approximately the lower/upper quartiles under a normal approximation), a threshold commonly used in grassland and dryland studies to balance ecological relevance with sufficient sample size of extreme events for robust estimation [9,36,52]."

(3) Why the resistance/resilience indices are appropriate for plant communities: We adopted the bounded resistance/resilience indices proposed by Orwin & Wardle (2004) because they are dimensionless, bounded (−1 to +1), and avoid unbounded ratios, enabling consistent comparisons across sites that differ in mean productivity. Although first introduced in the context of soil biota, the formulation is mathematically general and has been widely applied to ecosystem and plant-related response variables (e.g., productivity and carbon flux metrics). We have therefore updated the citations to include representative plant/ecosystem applications in addition to the original methodological reference, and we added a brief explanation of why these indices are appropriate for EVI-based productivity (**revised lines 439–446**).

"These indices are dimensionless, bounded (−1 to 1), and scale-invariant, enabling direct comparisons of stability components across sites that differ in mean productivity; they represent a general "impact-normalized" formulation of resistance and resilience that has been widely adopted in ecological stability studies [52,55-59]. Although initially applied in studies of soil communities [54], the underlying formulation is mathematically general and has been widely used to quantify resistance and resilience of ecological functions across ecosystems [55-59]; here we apply it to remotely sensed productivity to ensure cross-site comparability."

• *Lines 292-293, 302, 317:* ***<u>Climate and imagery data span 2000-2022, but plant surveys and trait measurements were in 2018.</u>*** *Why extend climate/imagery data to 2022, four years after trait sampling? Using data from years after trait measurements could lead to spurious correlations. Justify this temporal scope or revise analysis to use 2000-2018 data.*

Response: Thank you for raising this important concern. We agree that including years after the 2018 plant survey and trait measurements could, in principle, raise questions about temporal mismatch. We therefore **clarified our rationale** and **added a temporally matched robustness analysis**.

Our use of the 2000–2022 EVI/climate series is methodological rather than an attempt to link post-2018 climate to 2018 trait measurements. Specifically, the stability components resistance and resilience are derived from an event-based framework that requires (***i***) classifying each site–year into dry/normal/wet conditions using SPEI and (***ii***) pairing extreme years with appropriate preceding and subsequent years. As shown in **Fig. S19**, each site is classified year-by-year based on SPEI, and we then compute resistance and resilience relative to site-specific normal baselines while applying explicit rules for consecutive extremes to avoid ambiguous attribution. For example, if two consecutive extreme years are of different types (e.g., dry followed by wet), we exclude the earlier year from resilience calculations and the subsequent one from both resistance and resilience; when two or more consecutive extremes are of the same type, we calculate resilience only for the later year and only if it is followed by a normal year (Methods revised).

Critically, **a longer time series is necessary to yield a sufficient number of "valid" extreme–normal sequences under these criteria**. We then summarize resistance and resilience at the site level as the mean across all valid extreme events, so that site-level stability is not driven by one or two idiosyncratic years. This avoids cases where a site with only a single qualifying extreme year would have its resistance/resilience dominated by that year, which could either under-estimate or over-estimate the site's typical stability response. **Extending the window to 2000–2022 increases the number of qualifying events per site and therefore provides more robust and comparable site-level estimates—especially for resistance and resilience**.

To directly address the reviewer's concern, we also recomputed all stability components using the 2000–2018 window as a validation analysis. The stability components estimated from 2000–2018 are highly correlated with those estimated from 2000–2022 (**Fig. S21**), and the **trait–stability relationships remain qualitatively unchanged across regions, consistent with our main conclusions** (**Fig. S22 and S23;** $\boldsymbol{\beta}$ is similar or larger, but moving from plot to site reduces sample size and widens uncertainty, weakening statistical support). Likewise, mean annual temperature and precipitation calculated over 2000–2018 versus 2000–2022 are highly consistent (**Fig. S18**; $r > 0.9$, $\boldsymbol{P} < 0.001$), indicating that extending the window

does not materially change the climate covariates.

Accordingly, we retain 2000–2022 in the main analyses to improve estimation robustness—particularly for resistance and resilience—while presenting the 2000–2018 results as a dedicated robustness test in the Supplementary Information and clarifying these points in the revised Methods.

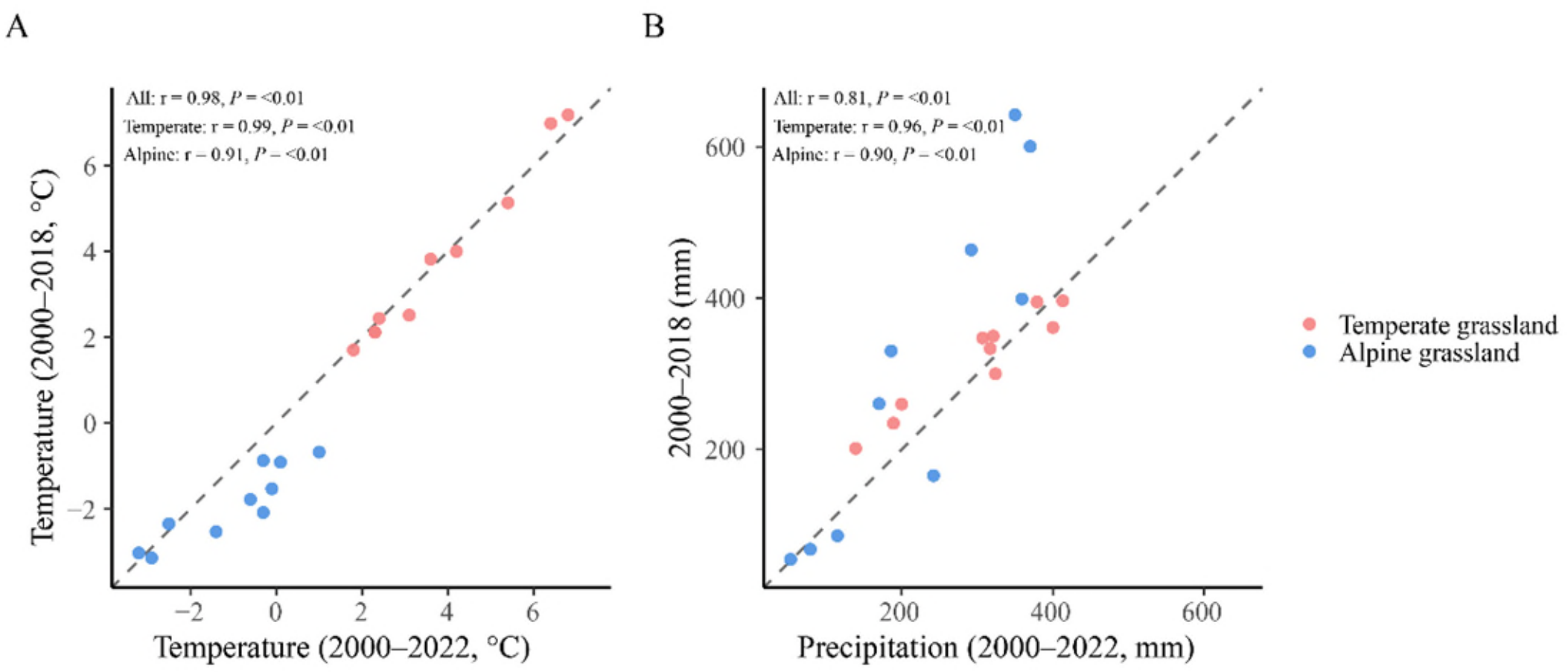

Figure S18. Comparison of site-level climate means between the full record (2000–2022) and the 2000–2018 window. Each point represents a site, coloured by region (temperate grassland vs. alpine grassland). Panels show (A) mean annual temperature and (B) mean annual precipitation. The dashed 1:1 line indicates perfect agreement between the two periods. Text in the upper-left of each panel reports Pearson correlations (r) and corresponding P values for all sites combined and for each region separately.

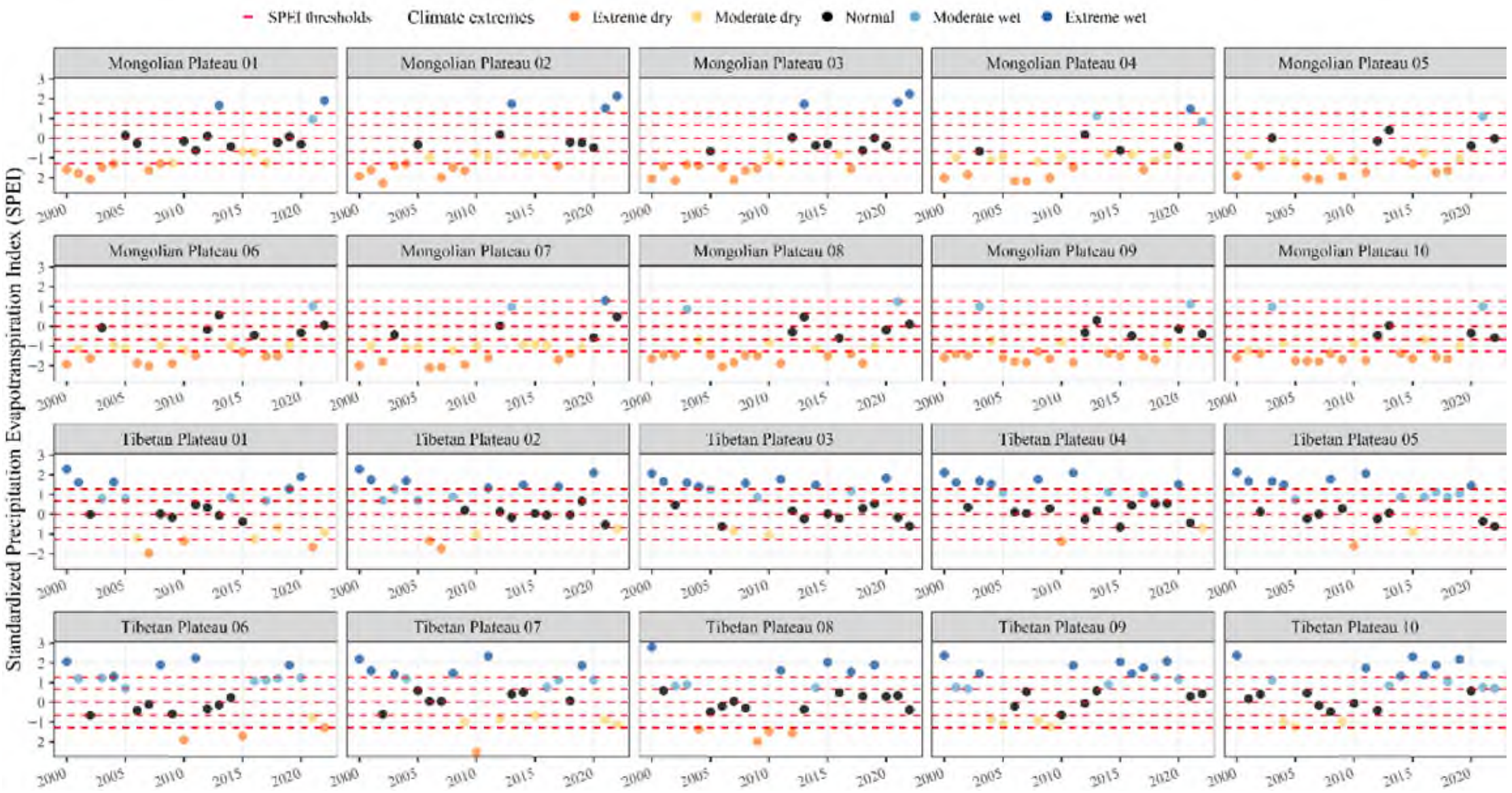

Figure S19. Temporal dynamics of climate extremes in temperate and alpine grasslands. Points show annual Standardized Precipitation Evapotranspiration Index

(SPEI; yearly mean) for each site (facets) across the temperate grassland (Mongolian Plateau) and alpine grassland (Tibetan Plateau) regions. Point colours indicate the climate categories (e.g., extreme dry, moderate dry, normal, moderate wet, extreme wet) defined in this study (legend). Red dashed horizontal lines mark the SPEI thresholds used to classify climate extremes (−1.28, −0.67, 0, 0.67, 1.28); values below/above these cut-offs represent progressively drier/wetter conditions.

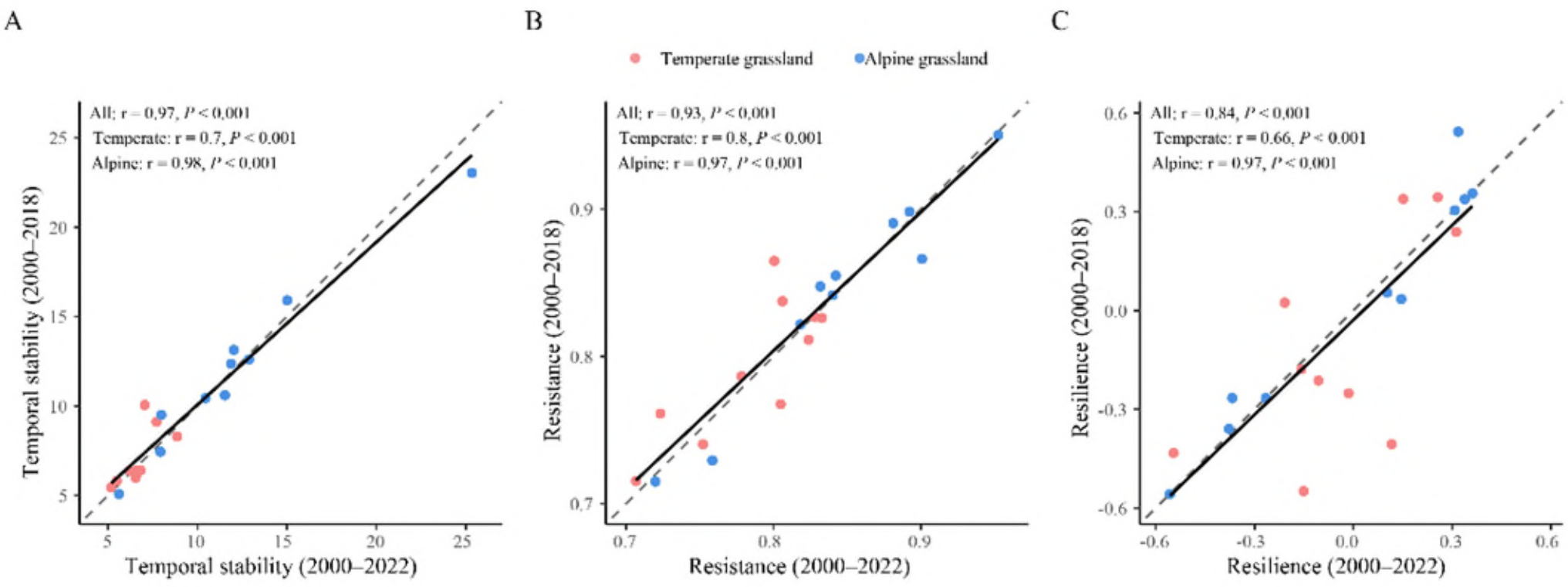

Figure S21. Consistency of EVI-based stability metrics between the full record (2000–2022) and the 2000–2018 subset. Panels show site-level comparisons of (A) temporal stability, (B) resistance, and (C) resilience calculated from MODIS EVI. The x-axis represents values computed using the full time series (2000–2022), and the y-axis represents values computed using the 2000–2018 subset. Points are colored by grassland type (temperate grassland, red; alpine grassland, blue). The dashed line indicates the 1:1 relationship, and the solid black line shows the fitted linear trend across all observations. Text in the upper-left reports correlation coefficients and ***P*** values for all sites combined and for each grassland type separately.

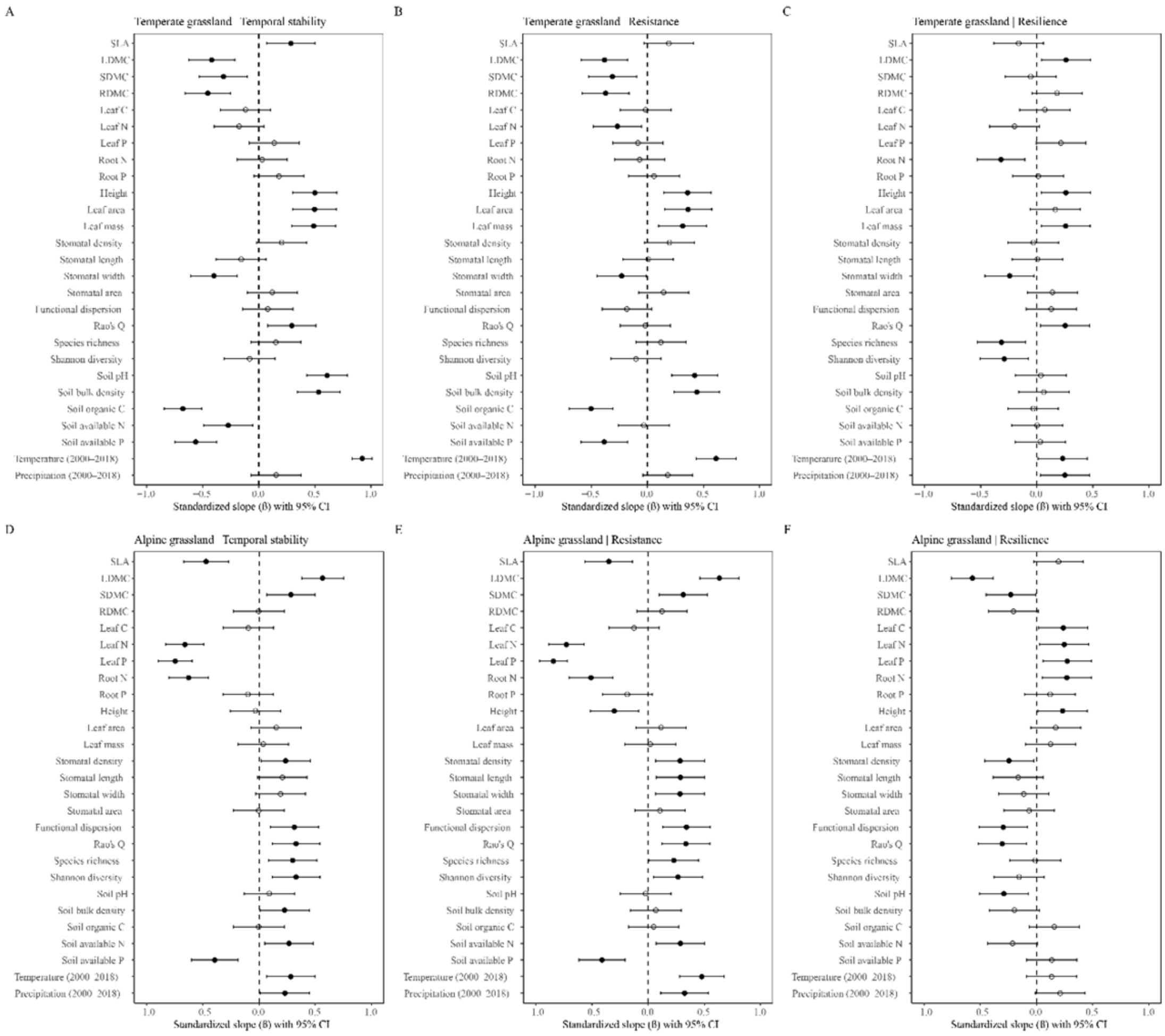

Figure S22. Robustness check using the corrected 2000–2018 window: standardized single-trait effects on three facets of ecosystem stability in temperate and alpine grasslands. Effects of abiotic factors (mean annual temperature and mean annual precipitation) and biotic factors—community-weighted mean (CWM) traits, functional diversity (functional dispersion, Rao's Q), and taxonomic diversity (species richness, Shannon diversity)—on three facets of ecosystem stability: temporal stability, resistance, and resilience. Each panel summarizes frequentist effect sizes from single-predictor linear models (ordinary least squares). Circles indicate mean standardized slope estimates (β); horizontal bars show 95% confidence intervals. All predictors were standardized (z-score; mean = 0, SD = 1) prior to analysis, and stability components were standardized after an optional log-transformation (controlled by an analysis switch; see Methods). Importantly, all three stability facets were calculated from the corrected 2000–2018 time series as a robustness analysis to test whether the trait–stability relationships are sensitive to the length of the observation window. Panels are arranged with temperate grasslands (Mongolian Plateau) in the top three panels and alpine grasslands (Tibetan Plateau) in the bottom three panels.

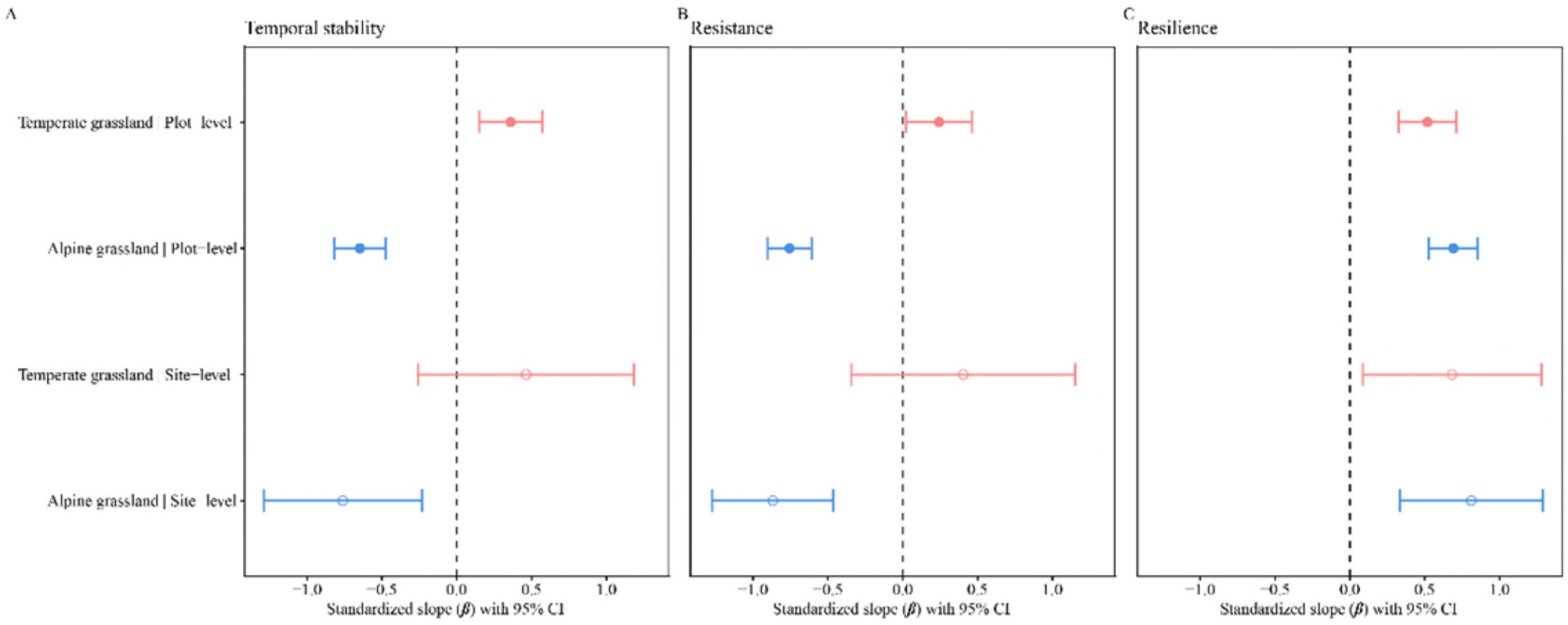

Figure S23. Robustness check using the corrected 2000–2018 window: economic strategy–stability relationships in temperate and alpine grasslands. Forest plots summarize standardized regression slopes (***β***) with 95% confidence intervals for relationships between an economic-strategy axis and three stability components derived from MODIS EVI over 2000–2018: temporal stability (TS), resistance (Rs), and resilience (Rl). Positive ***β*** indicates that higher values on the economic-strategy axis are associated with higher stability (or higher resistance/resilience), whereas negative ***β*** indicates the opposite. Colors denote habitat type (temperate vs alpine), and symbols distinguish analysis level (plot-level models vs site-level reanalyses using site-aggregated responses). Panels A–C correspond to TS, Rs, and Rl, respectively.

- *Supplementary Materials Figure S13: The relationship between biomass and EVI is not compelling, particularly for alpine grassland. At high biomass levels, there is very little predictive capacity. This raises questions about using EVI as a proxy for biomass in stability calculations. Either justify why EVI is appropriate despite weak relationships or acknowledge as a limitation.*

Response: We agree that Fig. S13 shows a weaker biomass–EVI relationship in the alpine grassland, particularly at high biomass, and we acknowledge this as a limitation. Nonetheless, EVI remains a practical and widely used proxy for interannual variability in vegetation productivity/greenness in large-scale, multi-site studies where repeated annual field biomass measurements are unavailable. In our dataset, long-term, spatially continuous EVI time series provide the only consistent measure for quantifying temporal stability across all sites, whereas biomass was measured only during the field campaign and thus cannot support stability calculations over multiple years.

To address this concern, we have expanded the Method to explicitly acknowledge the uncertainty introduced by imperfect biomass–EVI coupling, especially in alpine systems, and to clarify the scope of inference. We added the following text (**revised Method lines 399-409**):

“Long-term satellite-derived EVI provides a practical and standardized tool for

capturing interannual vegetation dynamics across multiple sites, given the lack of temporally replicated biomass measurements in the field. However, the biomass–EVI relationship is weaker in the alpine grassland, particularly at high biomass levels (Fig. S13). This limitation may introduce additional uncertainty and reduce predictive power in high-biomass alpine communities, and we cannot exclude the possibility that it influences estimated stability metrics. However, any such effects are more likely to increase noise rather than systematically bias stability patterns. Future work should directly quantify ecosystem stability using temporally repeated community and productivity surveys, which would allow more rigorous validation of remote-sensing proxies and strengthen inference on stability mechanisms."

- *Grazing disturbances not addressed: Worldwide, grasslands are historically heavily grazed and grazing intensity may vary with elevation and through time. The manuscript states sites have "no human disturbance," but provides no evidence of other disturbance types. Either quantify grazing at both sites and control for it in analyses, provide evidence that disturbance was minimal and equivalent across sites, or acknowledge as a limitation.*

Response: We acknowledge that grazing is a common and potentially important disturbance in grasslands, and that grazing intensity can vary across space (including with elevation) and through time. Because our transect survey spans a large geographic extent, we selected sites using prior local knowledge, especially information provided by local herders and regional experts, to target areas with minimal recent human disturbance. However, we agree that we cannot completely exclude the possibility that some sites experienced grazing impacts historically, or that low-level grazing occurred without being detected during site selection.

We have therefore revised the text to (***i***) clarify how site selection was conducted, (ii) avoid overstatement (replacing "no human disturbance" with "minimal/weak disturbance"), and (***iii***) explicitly acknowledge grazing history as a limitation. We also note that future work should quantify grazing intensity directly (e.g., stocking rates, dung density, grazing exclosures, or remote-sensing/land-use products) and incorporate it as a covariate.

Revised text (Methods; **lines 326-329**):

"Sites were selected to minimize recent human disturbance based on prior reconnaissance and local knowledge (including consultation with local herders and regional experts). Nevertheless, we cannot rule out historical grazing impacts at our study sites."

Added limitation (Discussion; **lines 298-305**):

"Finally, although our study sites were selected to minimize human disturbance, historical or low-level grazing may have occurred and could contribute to unexplained

variation in vegetation dynamics. Because spatially explicit, time-resolved grazing data were not available for all sites, we could not directly account for grazing effect. Future studies combining long-term vegetation monitoring with direct measures of grazing intensity (e.g., stocking rates, dung surveys, exclosure experiments, or land-use datasets) will be essential to disentangle grazing effects from climatic drivers and plant functional strategies."

- *Lines 280-290: Insufficient trait collection detail. Simply citing Pérez-Harguindeguy et al. is inadequate. Provide explicit details on collection and measurement protocols, including number of plants sampled per species, number of species per plot/site/grassland type, number of replicates per trait measurement and method of trait measurement. Include complete details in supplementary materials if space is limited.*

Response: We agree that citing Pérez-Harguindeguy et al. alone is insufficient for reproducibility. We have therefore expanded the Methods and added a dedicated section (Text S2) in the Supplementary Materials that provides trait-specific sampling and measurement protocols, including (***i***) the number of individuals and subsamples measured per species within each site (thereby capturing intraspecific variation across sites), (***ii***) the instruments and laboratory procedures used for C, N, and P analyses, and (***iii***) the formulas used to derive key traits (e.g., SLA, LDMC, and stomatal area). In addition, we restored and expanded the previously missing Supplementary Materials text and now explicitly summarize trait coverage by reporting the number of species with trait measurements per plot, per site, and by grassland type (Text S2).

"Supplemental text 4 Plant trait sampling and measurement protocols
We quantified plant functional strategies using a trait dataset collected during the 2018 field campaign, following trait-specific standardized protocols, and we provide full procedural details here to ensure reproducibility. In total, we measured 15 morphological, chemical, and stomatal traits for 343 vascular plant species (Table S2), including plant height (cm), leaf area (LA, cm^2), specific leaf area (SLA, $cm^2\ g^{-1}$), leaf dry matter content (LDMC, %), stem dry matter content (SDMC, %), root dry matter content (RDMC, %), leaf carbon concentration (Leaf C, %), leaf nitrogen concentration (Leaf N, %), leaf phosphorus concentration (Leaf P, $mg\ g^{-1}$), root nitrogen concentration (Root N, %), root phosphorus concentration (Root P, $mg\ g^{-1}$), stomatal length (μm), stomatal width (μm), stomatal density (no. mm^{-2}), and stomatal area (μm^2; derived). Plant height was measured in situ prior to harvest as the distance from the soil surface to the highest photosynthetic tissue (excluding inflorescences when applicable); for each species within a plot we measured 3–5 healthy individuals (depending on local abundance) and used the mean as the plot-level species value. For leaf morphological traits (LA, SLA, LDMC), we collected 6–10 fully expanded, healthy leaves per species (pooled across individuals when necessary), scanned them using a portable flatbed scanner (CanoScan LiDE 110, Japan), and quantified LA with ImageJ; leaves were then rehydrated to saturated mass by immersion in deionized water for 24 h at room temperature, gently blotted, weighed to obtain saturated fresh

mass, oven-dried at 60 °C for 48 h, and reweighed to obtain dry mass. LDMC (%) was calculated as 100 × (dry mass / saturated fresh mass), and SLA ($cm^2\ g^{-1}$) as LA / dry mass. For SDMC and RDMC, stem segments (main stem/culm tissue) and fine roots were sampled from 3–5 individuals per species when possible, cleaned of debris, brought to saturated fresh mass by soaking in deionized water until fully hydrated, blotted, weighed for saturated fresh mass, and then oven-dried at 60 °C for 48 h to determine dry mass; SDMC (%) and RDMC (%) were calculated as 100 × (dry mass / saturated fresh mass) for stem and root tissues, respectively. For elemental traits, dried leaf and root samples were ground to a fine powder using an agate mortar grinder; Leaf C (%) and Leaf N (%) and Root N (%) were measured using an elemental analyzer (Vario MAX CN, Elementar, Germany). For phosphorus, powdered samples were digested with concentrated nitric acid (68% HNO_3) using a microwave digestion system, and Leaf P and Root P ($mg\ g^{-1}$) were quantified by inductively coupled plasma optical emission spectrometry (ICP-OES). For stomatal traits, three leaf subsamples (replicates) per species were prepared; each replicate was imaged on both adaxial and abaxial surfaces when distinguishable, using a scanning electron microscope (S-3400N, Hitachi, Japan). Stomatal length and width were measured from calibrated micrographs, stomatal density was computed as the number of stomata per unit leaf area (mm^2) across multiple fields of view per replicate, and stomatal area was derived assuming an elliptical geometry: stomatal area = stomatal length × stomatal width × ($\pi/4$). When both leaf surfaces were measured, stomatal metrics were averaged across surfaces to yield a single species value. Species-level trait values were summarized as means of replicate measurements and then used in downstream community calculations (e.g., community-weighted means and functional diversity metrics; see Statistical Analyses). Because trait sampling was conducted in a single year (2018) to maintain consistent field and laboratory processing across the broad transect, these measurements primarily capture spatial (among-site) variation in trait expression and may not fully reflect interannual trait plasticity; we therefore acknowledge this as a limitation and discuss its implications for interpreting trait–stability relationships in the revised Discussion."

- *Lines 292-294, 303: Climate data spatial resolution unclear. For example, climate data from China Meteorological Data Service Centre appears very broad-scale. How were site-specific values derived? Specify exact data source, spatial resolution, how site-specific values were obtained/interpolated, justification for site-level climate being applied to plot-level analyses.*

Response: We agree that the spatial resolution and the derivation of site-specific climate covariates should be stated explicitly. We have revised the Methods to clarify that we used the CRU TS v4.09 gridded climate product (0.5° × 0.5°). For each site, we extracted monthly temperature and precipitation at the site coordinates (and then aggregated the monthly series to growing-season metrics; Text S4).

Because all eight plots within a site are located within the same ~1,000 × 1,000 m homogeneous area, climate properties are effectively constant within sites and thus do

not vary among plots. This does not preclude using climate as an explanatory variable in plot-level analyses: climate effects are identified from among-site contrasts, while non-independence of plot-level responses is addressed by explicitly accounting for the nested sampling design (e.g., by including a site-level random intercept, (1 | Site_id)). Under this formulation, site-level predictors are related to plot-level responses in a way that reflects the underlying structure of the data.

In addition, to provide a conservative robustness check, we repeated the key analyses at the site level using site-aggregated predictors where appropriate. The site-level results were consistent with the plot-level inferences, indicating that our conclusions are not sensitive to applying site-level climate covariates in plot-level models (see **Figure S10**). These clarifications and the corresponding text revisions are now reported in the main Methods and Supplementary Information.

Minor Issues
Introduction:
• *Difficult to understand results without reading methods and supplementary materials. It is suggested to expand explanation of community stability concepts and improve logical flow, particularly for transition from traits to ecosystem stability to grasslands is disjunct at Line 90.*
Response: Thank you for this helpful suggestion. To make the Introduction more self-contained and easier to follow without relying on the Methods/Supplementary, we revised the text in several places to clarify the core concepts of community/ecosystem stability and to improve the logical flow.

Specifically, we (***i***) expanded the stability definitions at the outset (temporal stability, resistance, and resilience; e.g., “Understanding the determinants of ecosystem stability, which encompasses multiple dimensions such as temporal stability (**the extent to which a system remains invariant over time),** resistance **(the capacity of a system to withstand perturbations**), and resilience (**the ability of a system to return to its original state following perturbations**)...”, **lines 44-48**) and reiterated these concepts where needed; (***ii***) strengthened the transition from traits to our study systems by adding a clear context-dependence statement that motivates our temperate vs. alpine comparison (e.g., “To address this gap, we examined how conservative and acquisitive plant strategies relate to multiple dimensions of ecosystem stability (temporal stability, resistance, and resilience) under contrasting environmental conditions represented by China’s temperate and alpine grasslands (Fig. S1). **lines 99-102**”). Together, these revisions improve clarity and ensure a smoother traits → stability → grassland-context narrative in the Introduction.

• *Line 98: Introduction mentions 15 traits; methods state 16. Correct and ensure consistency throughout.*
Response: Thank you for catching this inconsistency. We confirm that 15 functional traits were used in the analyses. We have carefully checked and revised the Introduction, Methods, and all related text/tables/figures to ensure the trait number is

consistently reported as 15 throughout the manuscript.

Methods:

• *Lines 281-282: Wording implies authors used existing dataset rather than collecting new data. Reword for clarity.*

Response: We revised the sentence to clarify that the trait data were collected/measured by our team during the 2018 field survey, following standardized protocols, and we direct readers to the Supplementary Methods for the full sampling and measurement details.

See lines 340-341:

"We measured plant functional traits in situ following standardized protocols (see Supplemental Text S2 for detailed sampling and measurement procedures) [50]."

• *Lines 284-286: Define all acronyms before first use (write out full terms first, then acronym in parentheses).*

Response: We have revised the manuscript to ensure that all abbreviations are defined at first mention in the main text. In particular, we now spell out each trait name when it first appears in the Methods and provide its abbreviation in parentheses (e.g., specific leaf area (SLA), leaf dry matter content (LDMC), stem dry matter content (SDMC), and root dry matter content (RDMC)), and we also ensure that abbreviations are defined where relevant in figure legends and tables.

• *Line 315: Missing citations for rationale for EVI metric.*

• *Missing R package citations and versions. Cite all R packages used with version numbers (brms, vegan, etc). Additionally, add R version session at the end of R code. Essential for reproducibility.*

Response: Thank you for these helpful suggestions. We have added appropriate citations supporting the rationale for using the EVI-based metric (see **line 409**) and provided citations with version numbers for all R packages used (e.g., brms [v2.23.0], vegan [v2.7.2]). We also added the full R session information (R version and package versions) at the end of the analysis code to ensure full reproducibility. The session information is:

R version 4.5.2 (2025-10-31 ucrt); Platform: x86_64-w64-mingw32/x64; Running under: Windows 11 x64 (build 26100). Key package versions include: brms 2.23.0, bayesplot 1.15.0, tidybayes 3.0.7, ggplot2 4.0.1, dplyr 1.1.4, and tidyr 1.3.2 (full sessionInfo() output provided in the revised Code).

Supplementary Materials:

• *Lines 268, 272 (and throughout): Incorrect supplementary references. Lines 268 and 272 refer to Table S1 and Figure S1 when they should be Table S2 and Figure S2. Systematically check and correct all supplementary citations.*

Response: Thank you for pointing out these errors. We have now systematically checked all Supplementary cross-references throughout the manuscript (tables and figures) and corrected the incorrect citations.

- *Supplementary materials labelling: Figure, table, and text labels are mixed up (e.g., Text 2 should be Text 3). Supplementary Text 3 lacks references for growing season calculations. Several supplementary figures are not described in main manuscript, either describe or remove them.*

Response: Thank you for these careful checks. We have thoroughly revised the Supplementary Materials to correct and standardize all labelling and cross-referencing. Specifically, we (***i***) fixed the mixed and inconsistent labels across Supplementary Figures, Tables, and Text sections (e.g., renumbering "Text 2" to the correct "Text 3" and updating all corresponding in-text citations); (***ii***) added the missing references in Supplementary Text 3 supporting the growing-season calculations; and (***iii***) systematically checked that each Supplementary Figure and Table is explicitly cited in the main manuscript (or, where appropriate, in the Supplementary text) and removed/merged any items that were not referenced. Collectively, these edits ensure that the Supplementary Materials are consistently labelled, properly referenced, and fully integrated with the main text.

Figures and references:

- *Figure quality: Many figures have poor resolution, overlapping text, and cut-off elements (e.g., bottom arrow in Figure 1). Ensure all figures meet journal quality standards.*

Response: Thank you for this suggestion. We have updated all figures to meet journal quality standards by: (***i***) Reformatting layouts to eliminate overlapping text and ensuring all graphical elements are fully visible (e.g., the bottom arrow in Figure 1 has been corrected); (***ii***) Standardizing font sizes and line weights to ensure consistency and readability across all panels; (***iii***) Re-exporting every main figure as high-resolution, vector-based PDF files. This ensures that all graphical elements and text remain sharp and scalable without any loss of resolution at any magnification level.

These changes substantially improve figure clarity and meet the rigorous presentation requirements of Nature Communications.

- *Reference formatting: Inconsistent formatting, some references missing DOIs. Ensure consistent style per journal guidelines and include DOIs where available.*

Response: Thank you for noting this. We have carefully checked and revised the reference list to ensure consistent formatting throughout in accordance with Nature Communications guidelines. We also added DOIs wherever available and verified that all citations are complete and consistently styled across the manuscript.

- *Permits: Confirm appropriate permits were obtained for plant biomass removal*

from both sites and state permit number.
Response: We confirm that all necessary permits and approvals for plant biomass sampling were obtained prior to fieldwork. The following statement has been added to the Methods section (**lines 336-338**):

"Field sampling was authorized by the Institute of Geographic Sciences and Natural Resources Research, Chinese Academy of Sciences (CAS), and conformed to local governmental regulations."

This institutional authorization covered the entire transect survey to ensure all activities complied with national and local ecological research standards.

Reviewer #2 (Remarks on code availability):

In a partial review, two issues were noted.

1) *it appears that the sampling design of plots nested within sites is not reflected in the analyses.*

Response: Where appropriate, we have revised the analysis code to explicitly reflect the hierarchical sampling design of plots nested within sites. Specifically, wherever plot-level responses were analyzed, we incorporated site as a grouping factor (e.g., random intercepts for Site) to account for within-site non-independence. In addition, we conducted complementary site-level analyses based on site-aggregated values to verify that our main inferences are robust to the sampling structure. These updates are described in the revised Methods/Results, and the corresponding updated scripts will be included in the public code repository in the next upload.

2) analyses produced interim data objects that the researchers apparently saved on their local drives. these were subsequently called and read by the code. For an independent analyst, these scripts could be run, but required significant interaction to reset interim data object names.
Response: We agree. We have refactored the code to remove dependencies on locally saved interim objects and hard-coded file paths. All intermediate datasets are now generated reproducibly within a single workflow and saved using a standardized project directory structure with relative paths. We also added clear input/output specifications, consistent object naming, and a "clean run" option that rebuilds all intermediate files from the raw data, so an independent analyst can execute the full pipeline end-to-end without manual renaming or interactive intervention. **We will update the public repository with the finalized code and complete source datasets once the temporary Figshare access issue is resolved (currently returning a "403 Forbidden" error from our side), replacing the interim version; if the issue persists, we will deposit the materials in an alternative open repository and provide the corresponding persistent link in the manuscript.**

Reviewer #3 (Remarks to the Author):

I co-reviewed this manuscript with one of the reviewers who provided the listed reports. This is part of the Nature Communications initiative to facilitate training in peer review and to provide appropriate recognition for Early Career Researchers who co-review manuscripts.

Response: We thank Reviewer #3 for co-reviewing this manuscript. In particular, we truly appreciate your time and effort devoted to providing the constructive comments and suggestions that help significantly improve our manuscript.

References:

1.
Conti, L., Valencia, E., Galland, T., Götzenberger, L., Lepš, J., E-Vojtkó, A. *et al.* (2023). Functional trait trade-offs define plant population stability across different biomes. *Proceedings of the Royal Society B*, 290, 20230344.

2.
Craven, D., Eisenhauer, N., Pearse, W.D., Hautier, Y., Isbell, F., Roscher, C. *et al.* (2018). Multiple facets of biodiversity drive the diversity–stability relationship. *Nature Ecology & Evolution*, 2, 1579-1587.

3.
Májeková, M., de Bello, F., Doležal, J. & Lepš, J. (2014). Plant functional traits as determinants of population stability. *Ecology*, 95, 2369-2374.

4.
Umaña, M.N., Salgado-Negret, B., Norden, N., Salinas, V., Garzón, F., Medina, S.P. *et al.* (2023). Upscaling the effect of traits in response to drought: The relative importance of safety–efficiency and acquisitive–conservation functional axes. *Ecology Letters*, 26, 2098-2109.

5.
Yan, P., He, N., Fernández-Martínez, M., Yang, X., Zuo, Y., Zhang, H. *et al.* (2025). Plant acquisitive strategies promote resistance and temporal stability of semiarid grasslands. *Ecology Letters*, 28, e70110.

Response to the Reviewers:

(Original comment and query are in italics and key comments are in bold; responses are in blue font)

Reviewer 1:

Reviewer #1 (Remarks to the Author):

The authors generally made appropriate responses to my previous review. I have several comments on the revised manuscript.

1) I remain uncertain about how the authors (and others in this field) link functional traits to ecological strategies such as stress tolerance. While traits associated with resource conservation (e.g. low SLA) can be associated with stress tolerance, they are also observed in low stress environments such as rainforests. While these environments are low stress, they tend to favour long-life spans and functional traits that are often associated with resource conservation. Perhaps this could be addressed in the manuscript <u>by limiting the discussion to grasslands on environmental gradients, as these ecosystems are functionally similar and functional differences are likely due to differences in abiotic stress</u>.

Response: Thank you for raising this important point. We agree that functional traits should not be interpreted as universal indicators of ecological strategies across all ecosystem types. As the reviewer points out, conservative traits such as low SLA can also occur in relatively low-stress environments where long tissue lifespan and slow turnover are favored. We have therefore revised the Discussion to avoid overgeneralizing the link between functional traits and stress tolerance, and now explicitly frame our interpretation within the context of grasslands along environmental gradients. The revised text reads:

"More broadly, we recognize that the same functional traits may reflect different ecological strategies across ecosystem types, such that traits associated with stress tolerance in grasslands may arise for different reasons in other systems. For example, conservative traits, such as low SLA, may also occur in relatively low-stress environments, including rainforests, where long tissue lifespan and slow turnover are favored. We therefore interpret conservative and acquisitive trait syndromes here within the more restricted context of grasslands along environmental gradients, where ecosystems are broadly comparable in growth form and where trait differences are more likely to reflect contrasts in abiotic stress, resource limitation, and life-history composition." (**lines 291-300**)

2) The key finding of this study is that conservative and acquisitive strategies can promote ecosystem stability. Is there any way we can predict which of these strategies is more likely to promote stability in a given ecosystem?

Response: Thank you for raising this important point. We agree that a clearer framework is needed for predicting when conservative versus acquisitive strategies

are more likely to promote ecosystem stability. We have revised the Discussion to clarify that the stabilizing role of a given trait strategy depends on the dominant environmental constraint and on whether stability is maintained primarily through resistance or resilience. The revised text reads:

"More broadly, our analyses suggest that the strategy most likely to promote stability depends on the dominant environmental constraint. Where stress is persistent and resource supply is chronically constrained, conservative traits may enhance resistance and temporal stability by maximizing stress tolerance. Where resources are less chronically limiting but fluctuate through time, acquisitive traits may enhance resistance and temporal stability by enabling strategies for stress escape and avoidance. The generality of these findings, however, remains to be tested across a broader range of ecological systems and environmental contexts" (**lines 226-234**)

3) In the alpine ecosystem traits associated with resilience are opposite to those that are associated with resistance. Is resilience or resistance more important for ecosystem stability?

Response: Thank you for raising this important point. We agree that the contrasting trait associations with resistance and resilience in alpine grasslands require clearer interpretation. To address this, we conducted an additional site-level analysis comparing the relationships of resistance and resilience with overall temporal stability. This analysis showed that resistance was more strongly associated with temporal stability than resilience in both temperate and alpine grasslands (**Figures S17 and S18**). Thus, although economic traits showed opposite associations with resistance and resilience in alpine grasslands, resistance appears to represent the dominant pathway linking traits to overall ecosystem stability.

We have added a dedicated statement in the Discussion to clarify this point:

"Note that although resistance and resilience reflect different components of stability, our supplementary site-level analysis suggests that resistance was more closely associated with temporal stability than resilience (Figure S18 and S19). Thus, the opposing trait associations with resistance and resilience in alpine grasslands likely translate into overall stability mainly through the resistance pathway. This pattern is consistent with emerging theoretical and empirical evidence that temporal stability can often be predicted more strongly from resistance than from post-disturbance resilience [37,50]." (**lines 245–252**).

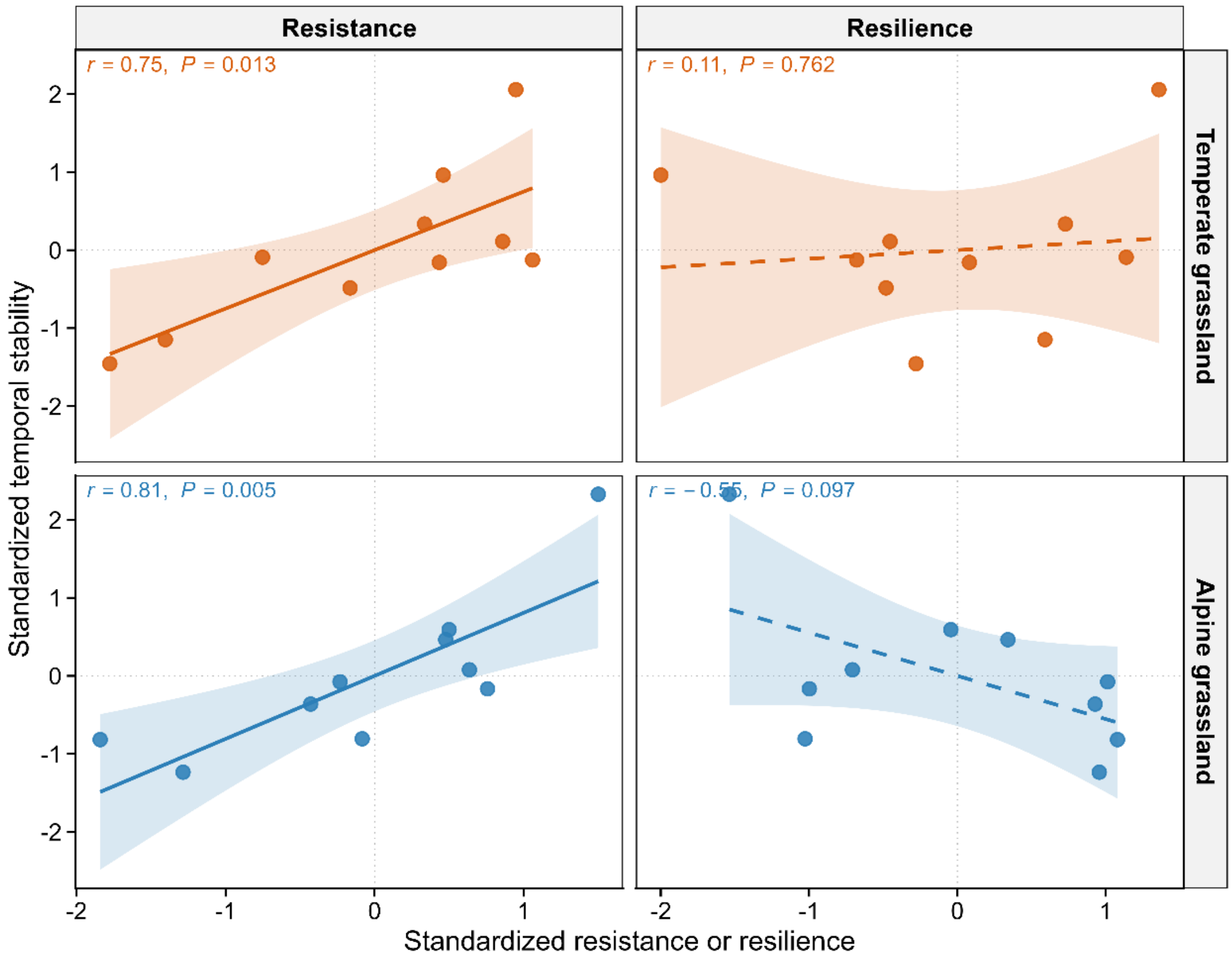

Figure S17. Site-level relationships between temporal stability and resistance or resilience across two grassland regions. Relationships between standardized temporal stability and standardized resistance or resilience were analyzed at the site level in temperate and alpine grasslands. Each point represents one site, with plot-level values first averaged within each site to avoid non-independence among plots from the same site. Temporal stability was quantified using the inverse coefficient of variation of EVI, while resistance and resilience were quantified using bounded stability metrics derived from EVI responses to climate extremes. All variables were standardized within each region before analysis to allow direct comparison of effect sizes. Solid and dashed fitted lines indicate significant and non-significant effects, respectively, based on region-specific multiple regression models including both resistance and resilience as predictors of temporal stability. Shaded bands represent 95% confidence intervals.

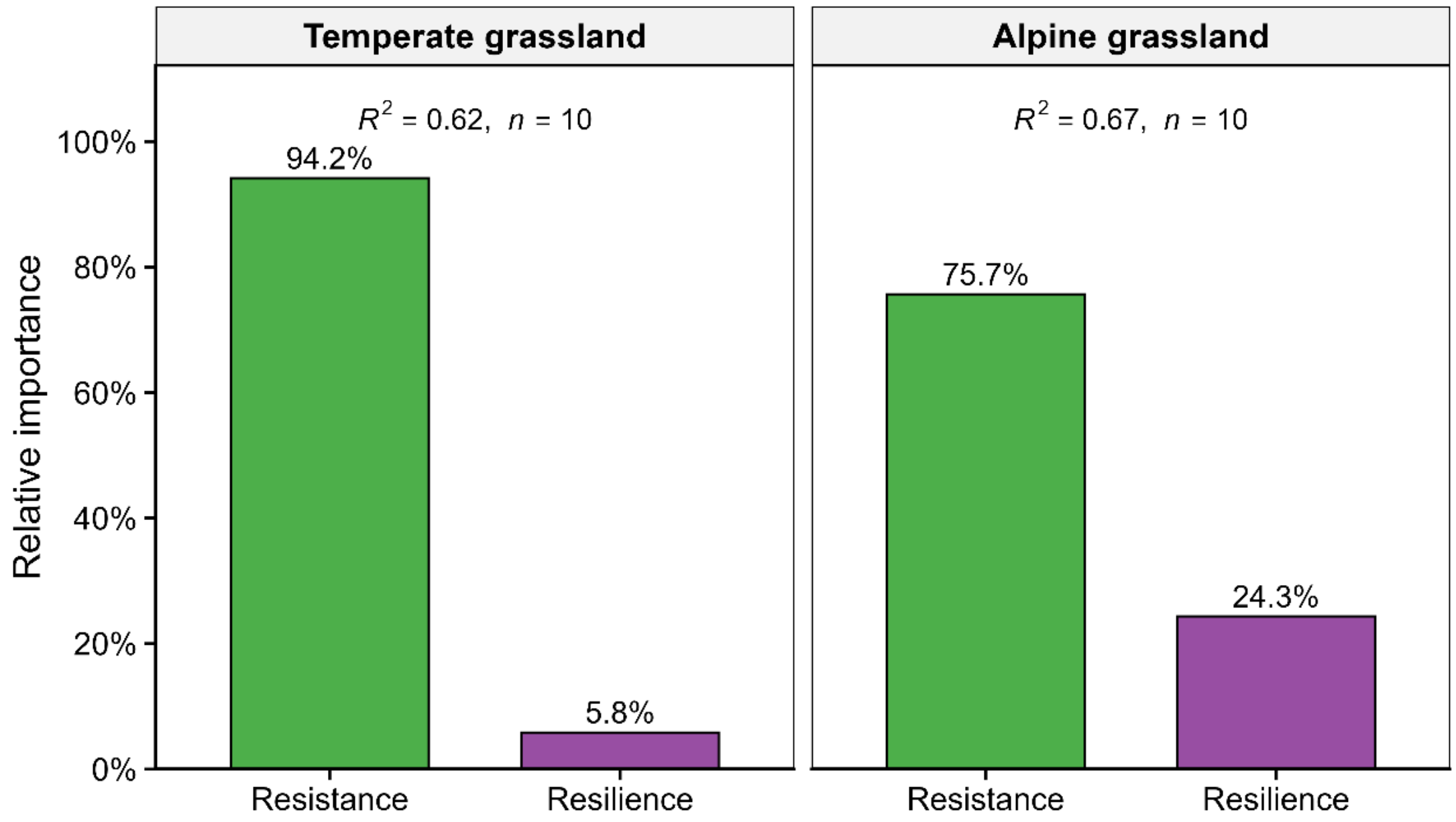

Figure S18. Relative contributions of resistance and resilience to temporal stability in temperate and alpine grasslands. Relative importance of resistance and resilience in explaining site-level temporal stability was quantified separately for temperate and alpine grasslands using region-specific multiple regression models. Temporal stability, resistance, and resilience were first averaged at the site level and then standardized within each region. Bars show the percentage contribution of each stability component to the explained variance in temporal stability, estimated using Lindeman–Merenda–Gold relative importance decomposition. Model R^2 and the number of sites are shown for each region. This analysis was used to evaluate whether resistance or resilience made the stronger contribution to ecosystem temporal stability in each grassland region.

4) Line 189 – "to our knowledge, this is the first empirical evidence ..." – can the authors confirm that they have done an appropriate search to support this statement?
Response: Thank you for raising this concern. We revisited the relevant literature and confirmed that, to our knowledge, previous empirical studies have not explicitly demonstrated opposing trait–stability relationships across different ecosystem types. We therefore retain the novelty of this finding, while adopting a more cautious formulation of our contribution. Specifically, we replaced "the first empirical evidence" with "unique empirical evidence." The revised sentence now reads:

"... our study provided unique empirical evidence of opposing trait-stability relationships across ecosystems." (**lines 184-186**)

5) Line 193-195 – add a reference for this statement?
Response: Thank you for pointing this out. We have added several relevant references to support this statement. Please see the revised manuscript, **lines 189–192**:

“Most importantly, our findings challenge the conventional paradigm that conservative, but not acquisitive, strategies foster ecosystem stability [4,6,22,40] and underscore the need for future studies to examine trait-stability relationships across various ecosystems.”

6) Lines 259-268 – add appropriate references.

Response: Thank you for this helpful suggestion. We have added references supporting the discussion of pH-mediated nutrient availability, nutrient limitation, and fertilization effects in temperate and alpine grasslands. The revised paragraph now reads (**lines 268–278**):

“... these contrasts suggest that different nutrient limitations may underlie stability in each ecosystem [53,54]. In temperate grasslands, elevated pH may alleviate phosphorus limitation, enabling plant communities to respond more dynamically to fluctuations in other limiting resources (e.g., water), thereby reducing ecosystem temporal stability [55]. In contrast, in alpine grasslands—where micronutrient availability is already low—higher pH may further restrict micronutrient access, potentially constraining plant responsiveness to other environmental variability and thus enhancing stability [54,56,57]. Long-term pH manipulation or nutrient addition experiments, coupled with repeated monitoring of plant traits and community composition, will be essential to clarify the causal links between soil chemistry and grassland stability [53].”

Reviewer 2:

Reviewer #2 (Remarks to the Author):

Thank you for the opportunity to review the revised manuscript. The authors have made a commendable effort in addressing most of the concerns raised in the first round of review, and several comments have been resolved satisfactorily. However, several serious concerns remain that must be addressed before the manuscript can be considered for publication. ***In particular, outstanding issues regarding pseudoreplication, model presentation, and reporting require attention.*** *Below we outline the major issues that remain to be addressed. Please note that the merged pdf document and the word document do not have the same content. Here we refer to the word document.*

Non-independence and hierarchical structure in models

Thank you for including site as a random effect; this is the appropriate approach for accounting for pseudoreplication. However, the plot-level random effects models continue to be presented as the main results throughout the manuscript. Doing so will lead to incorrectly estimating uncertainty around the parameters. This is a serious methodological concern, particularly for the SEMs. The plot-level models should not be presented as primary results. Furthermore, the methods section currently describes both the site-level and plot-level random effects models, which is confusing for the reader. We recommend removing the plot-level random effects model from the methods entirely; if the authors wish to retain this analysis, it should be moved to the supplementary material.

Response: Thank you for this important and constructive comment. We agree that plots within the same site are not independent observational units for analyses of ecosystem temporal stability, resistance, and resilience, and that presenting plot-level models as primary evidence could underestimate parameter uncertainty. We have therefore revised the manuscript so that all primary analyses involving temporal stability, resistance, and resilience are now conducted at the site level, with each site treated as the independent unit of replication.

Specifically, plot-level predictors, including community-weighted traits, soil properties, species composition, taxonomic diversity, and functional diversity, were aggregated to the site level before fitting the main regression models and Bayesian structural equation models. **The corresponding main-text figures, including Figures 2 and 3, have been replaced with site-level results.** Plot-level analyses involving temporal stability, resistance, and resilience are no longer presented as primary results; where retained, they have been moved to the Supplementary Information as suggested.

These changes have been implemented throughout the **Results**. For example, the revised text now states:

“At the site level, community-level economic traits were strongly associated with ecosystem temporal stability, resistance, and resilience, but the direction of these relationships differed between temperate and alpine grasslands. In temperate grasslands, a shift toward more acquisitive strategies was associated with greater temporal stability and resistance, whereas in alpine grasslands the same trait shift was associated with reduced temporal stability and resistance (Figure 2; Figure S6; Table S5 and S6). By contrast, resilience showed consistently positive relationships with economic traits in both grassland types (Figure 2; Figure S6; Table S5 and S6). Across the three stability components, community-weighted economic traits, including LDMC, leaf P, and SLA, had effects comparable to or exceeding those of abiotic factors, and substantially stronger than those of taxonomic and functional diversity (Figure S7 and S8). Bayesian structural equation models, which accounted for direct and indirect effects of climate and soil properties, further supported these site-level patterns (Figure 3), with model-predicted LDMC × temperature relationships shown in Figure S9. Supplementary plot-level analyses yielded qualitatively similar results (Figures S10 and S11; Table S7).” (**lines 150-165**)

We also revised the Methods throughout to clearly distinguish among observational levels. Plot-level analyses are retained only for variables intrinsically measured or summarized at the plot level, such as plant functional composition, species composition, soil properties, and diversity patterns. For these cross-sectional variables, site-level non-independence was addressed using site-clustered permutations or site random intercepts. In contrast, all analyses in which temporal stability, resistance, or resilience is the response variable are now described and interpreted as site-level analyses. For example, the revised Methods now state:

“... For variables quantified at the site level (e.g., temporal stability, resistance, and resilience, as well as site-level temperature and precipitation), we analyzed the site-level dataset with one record per site. These site-level models included grassland type as the predictor and allowed residual standard deviations to differ between grassland types, without including a site random effect because each site contributed only one observation...” (**lines 519-524**)

For the Bayesian structural equation models, we removed the plot-level SEMs from the main analysis and replaced them with pooled site-level BSEMs across temperate and alpine grasslands. Because the number of independent sites within each grassland type was limited, we did not fit separate grassland-specific site-level SEMs as the primary analysis. Instead, we included trait × temperature interaction terms to test whether trait–stability pathways varied along the main climatic gradient separating the two grassland types. Temperature was selected as the focal climatic moderator because it was the measured environmental variable that most clearly differentiated temperate and alpine grasslands, whereas precipitation and soil properties did not differ significantly between them (Figure S2; Table S2). We therefore did not include grassland type as an additional categorical moderator, or fit grassland type × trait

interactions, in the main BSEMs. **This modeling choice kept the site-level SEMs parsimonious, avoided over-parameterization under the limited number of independent sites, and reduced potential collinearity between a categorical ecosystem-type term and the continuous temperature gradient.** The resulting framework treats sites as the independent units of replication while directly evaluating whether trait effects on stability change along the dominant environmental gradient distinguishing the two grassland types. **The significant trait × temperature interactions further support our central conclusion that plant economic traits have ecosystem-type-specific effects on temporal stability, resistance, and resilience (Figure 3; Figure S9)**. As suggested, the plot-level BSEMs and BSEMs including the broader predictor set have been moved to the Supplementary Information.

Together, these revisions ensure that the main evidence for temporal stability, resistance, and resilience is based on the appropriate site-level unit of replication, while plot-level analyses are clearly framed as supplementary sensitivity analyses.

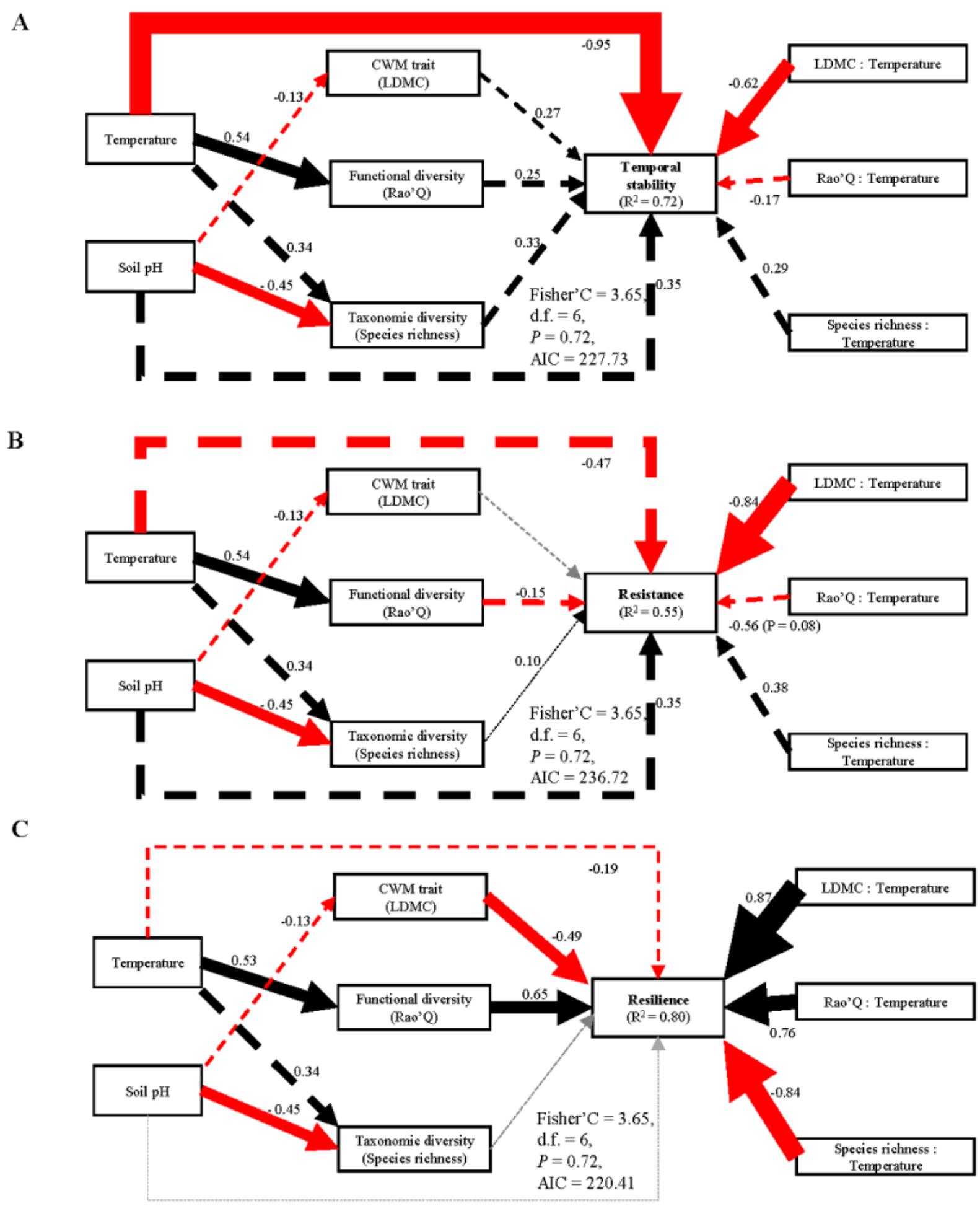

Figure 3. Results of Bayesian piecewise structural equation models fitted at the site level using pooled data from temperate and alpine grasslands, showing the direct and indirect effects of mean annual temperature, soil pH, taxonomic

diversity (species richness), functional diversity (Rao's Q), and leaf dry matter content (LDMC), including the LDMC × temperature interaction, on temporal stability (A), resistance (B), and resilience (C). LDMC was used as a parsimonious proxy for conservative–acquisitive plant strategies in these site-level models, and temperature was included as the focal climatic driver because it was the measured environmental variable that most clearly differentiated the two grassland types. Arrow widths correspond to the magnitude of standardized path coefficients. Black and red arrows indicate positive and negative relationships, respectively; solid lines represent statistically significant effects, while dotted lines denote non-significant paths. Fisher's C and d-separation statistics are shown as complementary diagnostics of model fit.

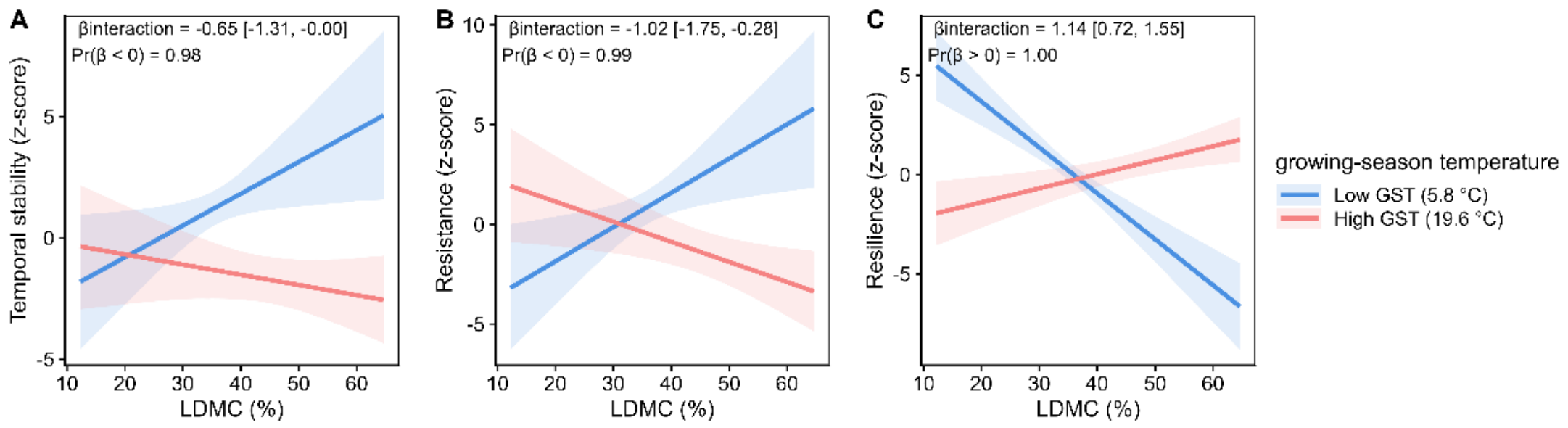

Figure S9. Bayesian estimates of the interactive effects of leaf dry matter content and growing-season temperature on ecosystem stability components. Model-predicted relationships between leaf dry matter content (LDMC, %) and standardized temporal stability (A), resistance (B), and resilience (C) are shown under low and high growing-season temperature (GST), corresponding to the temperature regimes of the alpine and temperate grasslands, respectively. **This temperature-based contrast is consistent with, and complementary to, the grassland-type moderation shown in Figure S6. Lines represent posterior mean predictions, and shaded bands indicate 95% credible intervals.** The annotation in each panel reports the posterior mean of the LDMC × GST interaction coefficient (β), with values below zero indicating that the LDMC effect becomes weaker or more negative at higher GST and values above zero indicating that the LDMC effect becomes stronger or more positive at higher GST. Values in brackets are 95% credible intervals. Pr(β > 0) or Pr(β < 0) denotes the posterior probability that the interaction coefficient is positive or negative, respectively; values ≥ 0.95 were interpreted as strong directional support for the sign of the interaction effect. Models included Rao's quadratic entropy, species richness, and soil pH as covariates. Response variables were standardized before model fitting.

Additionally, the raw R syntax in the manuscript text is not appropriate for a scientific publication (e.g. Method section, lines 799-806, lines 809-810). The model structure is still insufficiently explicit, and certain models, particularly the STMs, continue to be presented without adequate justification. Where a model formula is included, please ensure it is written correctly and that all terms, including sigma, are explicitly

defined (e.g. Method section, lines 809-810).

Response: Thank you for pointing out these issues. We agree that raw R syntax is not appropriate for the manuscript text and that the model structure should be described more explicitly. We have revised the Methods section to remove raw R syntax and replaced it with formal model descriptions and equations. These revisions now define the response variables, predictors, interaction terms, likelihoods, random effects where applicable, and model parameters, including the residual standard deviation parameter, σ, and the degrees-of-freedom parameter, ν.

The revised Methods now first describe the hierarchical models used for plot-level comparisons of cross-sectional variables, such as soil properties, species diversity, species composition, and functional composition. Throughout the model descriptions, we use *i* to index plots, *j* to index sites, *g* to index grassland type, and *p* to index predictors. Plot-level responses are denoted as y_{ij}, whereas site-level responses are denoted as y_j. Grassland type was consistently coded as temperate versus alpine grassland. These models are presented as:

$$y_{ij} \sim t_v(\mu_{ij}, \sigma_g),$$

$$\mu_{ij} = \alpha + \beta Grassland_j + b_j,$$

$$b_j \sim Normal(0, \sigma_{site}),$$

where y_{ij} is the response variable for plot *i* within site *j*, $Grassland_j$ denotes grassland type of site *j* (temperate or alpine), α is the intercept, β is the effect of grassland type, b_j is the site-level random intercept, σ_{site} is the standard deviation of site-level random effects, σ_g is the residual standard deviation allowed to differ between grassland types, and *v* is the degrees-of-freedom parameter of the Student's *t* distribution.

For site-level comparisons of temporal stability, resistance, resilience, and site-level climate variables, we now describe the model as:

$$y_j \sim t_v(\mu_j, \sigma_g),$$

$$\mu_j = \alpha + \beta Grassland_j,$$

where y_j is the site-level response variable for site *j*, $Grassland_j$ denotes grassland type, α is the intercept, β is the effect of grassland type, σ_g is the grassland-type-specific residual standard deviation, and *v* is the degrees-of-freedom parameter. Regional differences were considered significant if the 95% Bayesian credible intervals (CIs) excluded zero.

For the primary site-level regression models relating economic traits and other predictors to temporal stability, resistance, and resilience, we now provide the following general model structure:

$$y_j \sim t_v(\mu_j, \sigma),$$

$$\mu_j = \alpha + \sum_{p=1}^{P} \beta_p X_{jp},$$

where y_j represents temporal stability, resistance, or resilience at site j; X_{jp} represents standardized site-level predictors p for site j, including climate, CWM traits, taxonomic diversity, and functional diversity; P is the number of predictors, α is the intercept; β_p are standardized regression coefficients; σ is the residual standard deviation; and v is the degrees-of-freedom parameter of the Student's t distribution.

We also clarified the supplementary pooled interaction models (**Figure S6**), which test whether the effects of economic traits on temporal stability, resistance, and resilience differed between temperate and alpine grasslands:

$$\mu_j = \alpha + \beta_1 Trait_j + \beta_2 Grassland_j + \beta_3 Trait_j \times Grassland_j + \sum_{p=1}^{P} \gamma_p X_{jp},$$

where $Trait_j$ represents the focal economic trait at site j, $Grassland_j$ represents grassland type, β_3 quantifies whether the effect of economic traits on temporal stability, resistance, or resilience differs between temperate and alpine grasslands, and X_{jp} denotes additional standardized covariates at site j. These supplementary analyses yielded qualitatively similar conclusions as our site-level analyses and are presented in the Supplementary Information (**Figure S6**).

For the BSEMs, we now explicitly state that each endogenous variable was modeled using a Bayesian regression submodel of the general form:

$$z_j \sim t_v(\mu_j, \sigma),$$

$$\mu_j = \alpha + \textstyle\sum_{p=1}^{P} \beta_p X_{jp},$$

where z_j represents an endogenous variable in the site-level path model for site j; X_{jp} represents direct predictor p specified by the hypothesized causal structure, including the LDMC × temperature interaction where relevant, α is the intercept, β_p are standardized path coefficients, σ is the residual standard deviation, and v is the degrees-of-freedom parameter.

These equations have been added to the Methods section where the corresponding model classes are introduced (**lines 500-650**). We have also clarified the rationale for the streamlined site-level BSEMs. To avoid overparameterization under the limited site-level sample size, LDMC was selected as a parsimonious proxy for conservative–acquisitive plant strategies, and temperature was used as the focal climatic gradient differentiating the two grassland types. These revisions make the model structure and biological rationale more transparent.

Finally, the term "robust" should be removed when describing the Bayesian models in the methods section, as this terminology is unnecessary and imprecise in this context.

Citations for packages
Response: Thank you for this suggestion. We have removed the term “robust” when describing the Bayesian models in the Methods section. The models are now referred to as Bayesian models, Bayesian regression models, or Bayesian structural equation models, as appropriate. We also revised the description of the Student’s *t* likelihood to state more precisely that it was used to reduce sensitivity to potential outliers and heavy-tailed residuals under the relatively small site-level sample size.

Please ensure that R packages are cited correctly throughout the manuscript. For example, the brms package should be cited as: Burkner, P.C. (2017). brms: An R package for Bayesian multilevel models using Stan. Journal of Statistical Software, 80(1), 1-28. https://doi.org/10.18637/jss.v080.i01
Response: Thank you for noting this. We have checked the citations for all R packages and revised them where necessary to follow the *Nature Communications* reference style. In particular, we now cite the brms package in the required format as:

Bürkner, P.-C. brms: An R package for Bayesian multilevel models using Stan. J. Stat. Softw. 80, 1–28 (2017). https://doi.org/10.18637/jss.v080.i01.

We have also reviewed and updated the citations for other R packages used in the analyses where appropriate.

Code
We were unable to fully review the code; however, if the authors have ensured that the model structure is clearly described and that the code is reproducible for independent analysts, this is satisfactory.
Response: Thank you for this comment. We appreciate the reviewer’s acknowledgement. We have revised the Methods to make the model structure clearer and more transparent, including explicit descriptions of the response variables, predictors, interaction terms, error structures, and the distinction between the site-level main analyses and the plot-level sensitivity analyses. We have also checked the accompanying code to ensure consistency with the revised Methods and to improve reproducibility for independent analysts. The updated data files and scripts are organized to correspond directly to the revised analyses presented in the main text and Supplementary Information.

References cited:
1.
Conti, L., Valencia, E., Galland, T., Götzenberger, L., Lepš, J., E-Vojtkó, A. *et al.* (2023). Functional trait trade-offs define plant population stability across different biomes. *Proceedings of the Royal Society B*, 290, 20230344.
2.
Craven, D., Eisenhauer, N., Pearse, W.D., Hautier, Y., Isbell, F., Roscher, C. *et al.* (2018). Multiple facets of biodiversity drive the diversity–stability relationship.

Nature Ecology & Evolution, 2, 1579-1587.
3.
Májeková, M., de Bello, F., Doležal, J. & Lepš, J. (2014). Plant functional traits as determinants of population stability. *Ecology*, 95, 2369-2374.
4.
Umaña, M.N., Salgado-Negret, B., Norden, N., Salinas, V., Garzón, F., Medina, S.P. *et al.* (2023). Upscaling the effect of traits in response to drought: The relative importance of safety–efficiency and acquisitive–conservation functional axes. *Ecology Letters*, 26, 2098-2109.
5.
Yan, P., He, N., Fernández-Martínez, M., Yang, X., Zuo, Y., Zhang, H. *et al.* (2025). Plant acquisitive strategies promote resistance and temporal stability of semiarid grasslands. *Ecology Letters*, 28, e70110.
